# Rapid evolution can select for fitness tradeoffs in fluctuating environments

**DOI:** 10.64898/2026.09.01.748641

**Published:** 2026-09-04

**Authors:** John McEnany, Benjamin H. Good

**Affiliations:** 1 Biophysics Program, Stanford University, Stanford, CA 94305, USA; 2 Department of Applied Physics, Stanford University, Stanford, CA 94305, USA; 3 Department of Biology, Stanford University, Stanford, CA 94305, USA; 4 Biohub – San Francisco, San Francisco, CA 94158, USA

## Abstract

In fluctuating environments, the fates of new mutations depend on the fitness tradeoffs they experience across multiple environmental conditions. Selection on these tradeoffs is well-understood when mutations compete in isolation, but much less is known in the empirically relevant case where multiple mutations compete at the same time. Here, we develop a theory to predict how rapidly adapting populations select on fitness tradeoffs at many linked genetic loci. We derive analytical expressions showing how the fixation probabilities of these mutations depend on their underlying fitness tradeoffs, the timescales of environmental variation, and the future mutations they produce over time. We find that in large populations, competition between linked mutations can strongly favor mutations that carry larger fitness tradeoffs, even when they have a lower geometric mean fitness. We show that this “specialist advantage” arises because transient benefits enhance the rate of producing future mutations – an effect that continues to compound over time even when local conditions shift. We also demonstrate that successful lineages acquire well-timed mutations that appear to anticipate future environments, which can boost the rate of adaptation. These results show how large populations balance competing demands in temporally fluctuating environments, and provide a baseline for interpreting fitness tradeoffs in many natural and experimental settings.

## Introduction

Time-varying environments create a dilemma for natural selection: mutations that are beneficial in one environment often carry costs or tradeoffs in other settings (1). These tradeoffs can arise in diverse contexts (1–7), from seasonal variations in temperature (2) and resources (3), to the evolution of antibiotic resistance (4, 5). They can also arise from more subtle environmental shifts that preserve the overall direction of selection (7). Experimental advances have made it possible to map these fitness tradeoffs with increasing scale and precision, yielding joint fitness measurements for thousands of genetic variants across dozens of environmental conditions (4, 7–15). Yet despite this progress, it remains difficult to predict how evolution chooses between mutations with different tradeoffs when the environment varies over time.

There are two limiting cases where the effects of fluctuating environments are particularly well understood. When the environment changes more rapidly than the timescale of natural selection, the environmental fluctuations self-average, and the long-term dynamics are primarily determined by the time-averaged geometric mean fitness (16–21). Conversely, when the environment changes more slowly than the time it takes for new mutations to reach fixation, their fates will be determined by a constant local environment (20, 21), and any tradeoffs will accumulate nearly neutrally (5). However, there is also a third regime that lies between these two extreme limits, where the effects of time-varying environments become significantly more complex (21). This intermediate regime emerges whenever local environmental perturbations can generate large shifts in mutation frequencies, but the time to fixation remains long enough that mutational tradeoffs are eventually exposed. Empirical estimates suggest that this “strong tradeoff” regime is particularly relevant for microbes and other unicellular organisms, due to their fast generation times and large population sizes. In these rapidly evolving populations, even subtle shifts in the environment can generate local large fitness differences that act over days and weeks (7, 10, 11, 22), yet the size of the population is large enough that mutations take a long time to fix (23).

Previous work has explored these dynamics when mutations compete in isolation (21). However, in many empirical contexts, from laboratory evolution experiments (24–27) to natural populations of viruses (28–30), bacteria (11, 31–34) and certain cancers (35, 36), the supply of new mutations is large enough that multiple mutations will often arise and compete with each other at the same time. The competition between these linked mutations (“clonal interference;” (37, 38)) ensures that the fates of new mutations will not only depend on their own fitness tradeoffs, but also on the tradeoffs of the mutations that they happen to be linked to. This allows for qualitatively new phenomena that are absent from existing models of selection in time-varying environments. For example, mutant lineages can be rescued from unfavorable environments by acquiring further mutations that alter their net tradeoff profiles. Conversely, large populations can harbor “pre-adapted” lineages that are able to expand when the right conditions arise. These arguments suggest that the effects of linked selection may play a critical role in shaping which combinations of tradeoffs will eventually fix.

Here, we address this scenario by developing a mathematical model of rapid adaptation in a temporally varying environment. We focus on a simple and well-studied case, where the environment periodically fluctuates between a pair of environmental states. Within this setting, we develop an analytical approach to predict how the fixation probabilities of new mutations depend on the mutational landscape of their competitors, as well as the statistics of environmental change. We show that adaptive mutations with larger fitness tradeoffs have an advantage in founding further mutations, which can dramatically enhance their fixation probability even when they have a lower geometric mean fitness. We also show that when fitness tradeoffs are common, successful lineages tend to acquire mutations that (by chance) “anticipate” upcoming environmental changes, accelerating the adaptation of the population as a whole. These results suggest that large populations can exhibit unique signatures of adaptation in temporally fluctuating environments, where average fitness alone does not necessarily predict evolutionary success.

## Results

### Rapid evolution favors mutations with environmental tradeoffs

To study how environmental tradeoffs influence adaptation in time-varying environments, we focus on a simple setting where the environment periodically fluctuates between a pair of environmental states (Fig. 1A). Within this context, we consider a large, well-mixed population of N haploid individuals, which can acquire new mutations at a large number of linked genetic loci. The effect of each mutation can then be described by its incremental (log) fitness effect in each of the two conditions ($s_{1}$ and $s_{2}$). The collection of all such mutations defines a corresponding joint distribution of fitness effects (JDFE, Fig. 1B), which plays an analogous role to the classical DFE in a multi-environment setting. Generalist mutations that are independent of the environment will fall on the diagonal of this distribution $\left(s_{1}=s_{2}\right)$, while environment-sensitive mutations will generally have an additional off-diagonal component $\Delta s\equiv \left(s_{1}-s_{2}\right)/2$.

Previous work has shown that when the environment fluctuates sufficiently rapidly, the fates of each mutation will be determined by their geometric mean fitness effects,

(1) $$\overset{‾}{s}=\frac{\tau_{1}s_{1}+\tau_{2}s_{2}}{\tau_{1}+\tau_{2}},$$

where $\tau_{1}$ and $\tau_{2}$ denote the average time spent in each of the two conditions (Fig. 1B). This suggests a useful reparameterization of the JDFE for a given evolution environment, in which each mutation is characterized by its mean fitness $\overset{‾}{s}$ and its corresponding tradeoff Δs. Generalist mutations will lie on the x-axis of this distribution (Δs=0), while environment-sensitive mutations will fall either above or below this line (Δs≠0). In the main text, we will generally focus on the symmetric case where $\tau \equiv \tau_{1}=\tau_{2}$, but this simplification is not critical for our results (SI 2.1.5).

Following previous work (21), we will quantify the emergent selection pressures on these mutations by considering their long-term fixation probabilities $p_{\text{fix}}(\overset{‾}{s},\Delta s)$; this provides a natural measure of the strength of selection when many different mutations are available (39, 40). A central goal of this study will be to determine how these fixation probabilities depend on the underlying fitness tradeoffs and the timescales of environmental variation.

To address this question, we begin by considering a coarse-grained version of the two-dimensional JDFE in Fig. 1B, which contains three different types of mutations (Fig. 1C). Generalist mutations occur at a total rate $U_{g}$, and provide an average fitness benefit that is independent of the environment $\left(s_{g}\equiv s_{1}=s_{2}\right)$. We also consider a second class of environment-dependent “specialist” mutations; these occur at a total rate $U_{s}$, and confer an average benefit $\overset{‾}{s}$ as well as a corresponding tradeoff ±Δs (Fig. 1C). For simplicity, we work in the infinite sites limit and neglect the effects of epistasis, so that the JDFE remains constant as the population adapts. These assumptions define a minimal model for studying the effects of fitness tradeoffs on a fluctuating fitness “seascape.”

Figure 1C shows the measured fixation probabilities in simulations of this model (SI 4.1) for several different population sizes and switching conditions. In previous single-locus models (21), the fixation probabilities of beneficial generalists often exceed their specialist counterparts, until the latter start to have an appreciable chance of fixing in a single environment. In contrast, the simulations in Fig. 1C show that the opposite behavior occurs in large populations where multiple mutations compete at the same time ($NU_{\text{tot}}\gg 1$, teal curves). In these settings, the fixation probability of an environment-sensitive mutation can be ~10–100x larger than a corresponding generalist mutation with the same geometric mean fitness. These advantages are largest for strong fitness tradeoffs (Δs⋅τ≫1) and vanish when Δs⋅τ≪1. When environmental switching is stochastic, the benefits of specialism grow even larger (Fig. 1C).

This behavior is counterintuitive under existing theories that emphasize the (realized) time-averaged fitness. As the population size becomes larger, new mutations take longer to reach fixation, and the examples in Fig. 1C experience multiple environmental cycles before they are able to fix. During this time, successful mutations spend a roughly equal time in both favorable and unfavorable environments, and their trajectories fluctuate strongly due to environmental change and further evolution in the population (Fig. 1D). But rather than averaging out (as in the single-locus case), these transient fluctuations seem to provide a strong systematic advantage to mutations with strong tradeoffs. How does this “specialist advantage” emerge when multiple mutations compete for fixation?

### Modeling rare mutations with tradeoffs in a perfectly periodic environment

To understand the origin of the specialist advantage in Fig. 1, it is useful to begin by considering a simpler case, where specialist mutations are rare $\left(U_{s}\to 0\right)$ but generalist mutations are still common $\left(NU_{g}\gg 1\right)$. In this case, we can consider the fates of individual specialist mutations as they compete against a diverse generalist background (Fig. 2A). Crucially, both the specialist strain and the background population will continue to acquire generalist mutations, but their environmental tradeoffs will remain fixed as the environment-sensitive mutant competes for fixation.

Simulations confirm that as before, rare environment-sensitive mutations have a marked fixation advantage over generalists. Intriguingly, this advantage is strongly dependent on when the specialist mutation arises: the fixation probability is maximized for mutations which establish near the start of a beneficial epoch, and decreases exponentially for later times (Fig. 2B). The time-averaged fixation probability is dominated by mutations that establish near the optimal time, at the peak of the two-sided exponential. In order to account for this time dependence, we will need a method to analyze competition between strains.

A useful approach is to construct a fitness distribution by sorting individuals into fitness classes, based on their beneficial mutation count. In the absence of the specialist strain, past work (41) has shown this fitness distribution forms a “traveling wave” which advances at the rate of adaptation $v\ll \sqrt{s_{g}}$ (Fig. 2A). Evolution in this regime is driven by mutations in the high-fitness individuals at the “nose” of the wave, whose fitness is $x_{c}\gg s_{g}$ higher than the population mean. These results apply for a broad range of parameters applicable to large microbial communities, $1\ll NU_{g}\ll Ns_{g}$ (SI 1.1). Importantly, these traveling wave results will still hold in the presence of a low-frequency specialist strain, allowing us to express v and $x_{c}$ in terms of other evolutionary parameters (SI 1.1). Henceforth, we will make the additional assumption that $\Delta s\ll x_{c}$, so the tradeoff strength is not so strong that it overpowers all other fitness variation in the population. This assumption does not limit the relative size of $\overset{‾}{s}$ and Δs, so specialist mutations can still have strong tradeoffs relative to their average benefit.

If a specialist mutation arises on a high-fitness background close to the nose of the traveling wave, it will begin rising in frequency deterministically as long as it escapes initial genetic drift. The instantaneous growth rate of this genotype depends on its relative fitness, which changes whenever the environmental state does. In addition, this relative fitness will experience an overall decline at rate v, caused by adaptation in the rest of the population. When the focal genotype’s fitness is surpassed by the bulk population’s, it will begin falling in frequency, forming a parabolic log frequency trajectory punctuated by environmental change (Fig. 2C). Thus, the specialist (or indeed, any) mutation can only survive if its offspring produce their own beneficial mutations, keeping up with adaptation in the rest of the population even as each individual genotype in the lineage goes extinct. The specialist advantage, and its dependence on establishment time, can be explained through this process of founding future mutations.

#### Rolling fitness of a lineage.

To study the dynamics of how an environment-sensitive lineage founds new beneficial mutations, we will extend the successive establishment picture analyzed in Ref. (42) to the case of fluctuating environments. We consider a new specialist mutation which establishes at time t on a high-fitness genetic background, such that its relative geometric mean fitness is initially $x\sim x_{c}$. The specialist genotype will rise in frequency deterministically, eventually producing a new generalist mutation that survives initial genetic drift. Crucially, the descendants of this mutation will effectively act like a new specialist lineage which establishes at shifted relative average fitness $x^{\prime }$ and time $t^{\prime }$, given by

(2a) $$x^{\prime }=x+s_{g}-vT,$$

(2b) $$t^{\prime }=t+T,$$

where T represents the number of generations between the establishment of the initial lineage (at average fitness x) and its offspring (at $x^{\prime }$). Previous work (42) demonstrates that the fixation probability of the initial lineage can be approximated by solving the recursive “rolling fitness” relations in Eq. (2) over a large number of founding steps – predicting whether the descendants of a lineage will tend to increase their fitness until they reach fixation, or be outcompeted by the bulk population and go extinct.

All the complexities of growth in a changing environment have been absorbed into T, the waiting time for the next mutation. While T is a random variable, it is sufficient for our purposes to consider the *typical* time it takes the lineage to establish its first mutation, which we will also denote as T for convenience. It is most convenient to write the solution for T under the additional assumption that $\tau \gg s_{g}/v$, in which case t and $t^{\prime }$ will usually be in the same environmental state given by the indicator function I(t)=±1. In this case, T takes the form

(3) $$T(x,t)\approx \frac{s_{g}}{v}\left(1-\frac{x+\Delta s\cdot I(t)-x_{c}}{x_{c}}\right)$$

to leading order in $\left(x-x_{c}\right)/x_{c}$ and $\Delta s/x_{c}$ (SI 2.1.1). Eq. (3) indicates that mutations in a beneficial environment with I(t)=+1 rise in frequency faster and found beneficial mutations sooner (Fig. 2C). A similar effect is achieved if the mutation starts on a higher-fitness background with larger x. Meanwhile, Eq. (2) shows that a mutation which arises sooner (smaller T) lands at a higher rolling fitness $x^{\prime }$ because the rest of the population has had less time to adapt. Crucially, these effects compound over successive foundings, such that small differences in initial fitness increase over time:

(4) $$x^{\prime }\approx x+\frac{s_{g}}{x_{c}}\left(x-x_{c}\right)+\frac{s_{g}\cdot \Delta s}{x_{c}}I(t).$$

The recursive solution to Eq. (4) is particularly simple for generalist lineages with Δs=0, where mutations that start above the “break-even fitness” $x_{c}$ will tend to fix deterministically, while those below $x_{c}$ will be likely to go extinct (42). Specialist lineages experience a similar compounding effect, but are also sensitive to the environmental state I(t) during each founding step. Thus, their rolling fitness after n successive foundings can be written as a weighted sum of the environmental states they experienced during each step:

(5) $$x(n)\approx x_{c}+e^{ns_{g}/x_{c}}\left[x(0)-x_{c}+\frac{s_{g}\Delta s}{x_{c}}\sum_{i=0}^{n-1}e^{-(i+1)s_{g}/x_{c}}I\;(t(i))\right].$$

The influence of the environment in early steps soon after the specialist arises (smaller i) has more time to compound over future foundings, so those terms have the most weight in the sum (SI 2.1.1). Many cycles after the specialist mutation was founded $\left(n\gg x_{c}/s_{g}\right)$, we can exploit the periodic structure of I(t) to simplify the sum in Eq. (5) further:

(6a) $$x(n)\approx x_{c}+e^{ns_{g}/x_{c}}\left[x(0)-x_{c}+s_{\text{eff}}\left(t_{0}\right)\right],$$

(6b) $$s_{\text{eff}}\left(t_{0}\right)\equiv \frac{v\Delta s}{x_{c}}\left(\frac{\tau }{2}-\text{min}\left(t_{0},2\tau -t_{0}\right)\right),$$

where $t_{0}$ is defined such that the environment is beneficial between $t_{0}=0$ and $t_{0}=\tau$, and deleterious from $t_{0}=\tau$ to 2τ. This relation can also be shown to hold in the faster-cycling $\tau \ll s_{g}/v$ regime, where individual foundings span multiple environmental epochs (SI 2.1.1).

Eq. (6a) indicates that the long-term fixation probability of a specialist mutation $p_{\text{fix}}(\overset{‾}{s},\Delta s)$ coincides with that of a generalist mutation with shifted fitness effect $p_{\text{fix}}\left(\overset{‾}{s}+s_{\text{eff}}\left(t_{0}\right),0\right)$. The shift $s_{\text{eff}}\left(t_{0}\right)$ can therefore be interpreted as an effective fitness boost experienced by lineages with environmental tradeoffs, depending on the time $t_{0}$ they arise. This shift is positive if $t_{0}$ is near the start of a beneficial epoch, and negative if $t_{0}$ is near the start of a deleterious epoch, switching sign halfway through each environmental state. This behavior is consistent with the observed time dependence in Fig. 2A and the weighted sum in Eq. (5), favoring mutations which experience a full beneficial epoch immediately after they arise. Thus, even after many cycles, the time the lineage initially arose strongly influences its fate.

A specialist mutation which arises at the optimal time will have $s_{\text{eff}}=\Delta s\cdot v\tau /2x_{c}$, much smaller than the direct benefit Δs it would experience in a permanent favorable environment. However, this boost will still have a large impact on the mutation’s fixation probability, which we discuss next.

#### Fixation probability of a general mutation.

The overall fixation probability can be calculated by marginalizing over the fitness of the possible genetic backgrounds the mutation could arise on:

(7) $$p_{\text{fix}}\left(\overset{‾}{s},\Delta s|t_{0}\right)=\int f\left(x\right)\cdot p_{\text{fix}}\left(\overset{‾}{s},\Delta s|t_{0},x\right)\;dx,$$

(8) $$\approx \int f\left(x\right)\cdot p_{\text{fix}}\left(\overset{‾}{s}+s_{\text{eff}}\left(t_{0}\right),0|x\right)\;dx,$$

where $f(x)\sim e^{-x^{2}/2v}$ is the background fitness distribution in Eq. (S4). Past work (39) has shown that the integral in Eq. (8) is dominated by backgrounds where $x+\overset{‾}{s}+s_{\text{eff}}\approx x_{c}$, because fixation is strongly suppressed at lower fitnesses, but higher-fitness backgrounds are much rarer in the population. Therefore, a well-timed specialist mutation (with larger $s_{\text{eff}}$) can reach fixation on slightly lower-fitness backgrounds (smaller x; SI 2.1.4). Because these lower-fitness backgrounds are exponentially more frequent in the population, the overall fixation probability has a significant dependence on establishment time:

(9) $$p_{\text{fix}}\left(\overset{‾}{s},\Delta s|t_{0}\right)\approx \frac{x_{c}}{\overset{‾}{s}}\sqrt{\frac{2v}{\pi }}\text{exp}\left[-\frac{1}{2v}{\left(x_{c}-\overset{‾}{s}-s_{\text{eff}}\left(t_{0}\right)\right)}^{2}\right].$$

Since rare environment-sensitive mutations arise uniformly over time, integrating over $t_{0}$ will yield the overall fixation probability. This average fixation probability is dominated by mutants that arise near the start of a beneficial environment, yielding

(10) $$\text{log}\left(\frac{p_{\text{fix}}(\overset{‾}{s},\Delta s)}{p_{\text{fix}}(\overset{‾}{s},0)}\right)\approx \frac{\Delta s\cdot \tau }{2}-\text{log}\;(\Delta s\cdot \tau ).$$

Thus, environment-sensitive mutations have an exponentially enhanced fixation probability scaling with Δs⋅τ (Fig. 3A), systematically outcompeting generalist mutations with the same geometric mean fitness. This result holds when $1/\Delta s\ll \tau \ll x_{c}/v$, corresponding to the strong-tradeoffs regime where the environment changes many times before the mutant reaches fixation. A similar result applies in the case of asymmetric environments with $\tau_{1}\neq \tau_{2}$, where the harmonic mean of $\tau_{1}$ and $\tau_{2}$ becomes the relevant timescale (SI 2.1.5).

### Effects of environmental stochasticity on rare lineages with tradeoffs

Environmental fluctuations are in general not perfectly periodic. We can account for this by modeling the amount of time the environment stays in one state as a random variable with mean τ and variance $\sigma_{\tau }^{2}$, e.g. $\text{Gamma}\left(\tau^{2}/\sigma_{\tau }^{2},\sigma_{\tau }^{2}/\tau \right)$, which we use in simulations. The ratio $\sigma_{\tau }/\tau$ sets the strength of environmental unpredictability or seasonal drift: $\sigma_{\tau }/\tau =1$ represents a memoryless environment which has an equal chance of switching at any time, while $\sigma_{\tau }/\tau \ll 1$ corresponds to a mostly periodic environment with small amounts of stochasticity.

Strikingly, even a modest amount of environmental stochasticity, $\sigma_{\tau }/\tau \ll 1$, increases the fixation probability of rare specialist mutations by an order of magnitude (Fig. 2B). To explain this, we can extend our analysis of successive mutations to account for seasonal drift. A lineage which spends longer in a beneficial environment will grow and found new mutations faster, so the rolling fitness will change stochastically each environmental cycle based on whether the beneficial or deleterious period lasted longer. When the stochasticity is not too strong, we can express its effect in terms of the time differences δτ between successive epochs (SI 2.2.1). This results in a modified version of Eq. (6a), where an environment-sensitive lineage experiences an effective fitness shift $s_{\text{eff}}^{\text{stoch}}$ based on the realized stochasticity δτ:

(11) $$s_{\text{eff}}^{\text{stoch}}(\Delta s,\delta \tau )\equiv \Delta s\cdot \frac{v\tau }{x_{c}}\sum_{i=0}^{x_{c}/s}e^{-2(i+1)v\tau /x_{c}}\frac{\delta \tau_{i}}{\tau },$$

valid in the slow-cycling $\tau \gg s_{g}/v$ regime (see SI 2.2.1 for the more general case). This is a weighted sum of the environmental stochasticity, with more weight placed on earlier cycles (smaller i) – similar to the weighted sum over environments in Eq. (5), where a bias toward beneficial environments soon after the lineage establishes acts like a long-term fitness benefit. Note that in principle, the true $s_{\text{eff}}$ in a stochastic environment should be some combination of the deterministic shift in Eq. (6b) and the stochastic shift in Eq. (11); here, we focus on the regime where the stochastic part dominates.

The overall fixation probability now requires marginalizing over genetic backgrounds and environmental stochasticity,

(12) $$p_{\text{fix}}(\overset{‾}{s},\Delta s)\approx {\left\langle \int f(x)\cdot p_{\text{fix}}\left(\overset{‾}{s}+s_{\text{eff}}^{\text{stoch}}(\Delta s,\delta \tau ),0\right)dx\right\rangle }_{\delta \tau },$$

where the interior integral will once again be dominated by genetic backgrounds near $x\approx x_{c}-\overset{‾}{s}-s_{\text{eff}}^{\text{stoch}}$. To average over environmental stochasticity, it is reasonable to invoke the central limit theorem if the underlying distribution of δτ is not too wide-tailed, approximating $s_{\text{eff}}^{\text{stoch}}\sim \sigma_{\text{tot}}𝒩$ with 𝒩 a standard normal random variable and

(13) $$\sigma_{\text{tot}}\equiv \frac{\Delta s\cdot \sigma_{\tau }}{2\tau }\sqrt{\frac{2v\tau }{x_{c}}}.$$

Numerically solving Eq. (12) under this normal approximation recapitulates the extreme specialist advantage observed in simulations (Fig. 3B–C), where specialist mutations can even be 10^10^ more times likely to fix as generalists. This is because lucky sequences of environmental fluctuations give environment-sensitive lineages on lower-fitness backgrounds a viable path to fixation, significantly widening the range of lineages that can fix. For modest environmental fluctuations $\Delta s\cdot \sigma_{\tau }≲\sqrt{x_{c}\tau }$, the specialist advantage is

(14) $$\text{log}\left(\frac{p_{\text{fix}}(\overset{‾}{s},\Delta s)}{p_{\text{fix}}(\overset{‾}{s},0)}\right)\approx \frac{x_{c}}{4v\tau }{\left(\Delta s\cdot \sigma_{\tau }\right)}^{2}$$

to leading order. Because the $x_{c}/v\tau$ prefactor is large, even weak environmental stochasticity $\left(\Delta s\cdot \sigma_{\tau }\ll 1\right)$ can significantly influence fixation probabilities.

The expectation in Eq. (12) can be dominated by rare environmental fluctuations strongly biased toward beneficial environments, which may not be observed in practice (SI 2.2.1). However, these rare events are not critical for our qualitative results, because even typical realizations of environmental stochasticity continue to provide a large advantage to mutations with tradeoffs (Fig. S1). Another possibility is that the distribution of δτ is wide-tailed enough for the normal approximation to break down, in which case the fixation probability can be dominated by long beneficial epochs where the mutation appears to provide an unconditional benefit of $\overset{‾}{s}+\Delta s$ (Fig. S2; SI 2.2.2). More generally, Eq. (12) still applies for more complicated underlying distributions of δτ, such as correlated epoch durations (43). These details do not change the overall picture that small levels of environmental stochasticity can provide huge benefits for a rare lineage sensitive to environmental change.

Note that the effects of stochasticity discussed in this section and Figure 3B–C are much larger than those observed in Figure 1C. The reason for this difference is our assumption that specialist mutations are rare: in this case, only one lineage at a time can benefit from environmental stochasticity, drastically increasing its fixation probability. When specialist mutations are widely available, this will no longer be the case, which we discuss later.

### Frequent mutations with tradeoffs in a periodic environment

Generally, mutations with substantially different fitness effects between environments are not always rare relative to generalist mutations. When specialist mutations are common $\left(NU_{s}\sim NU_{g}\gg 1\right.$), then the dynamics of the entire population will be reshaped by environmental change, so the one-dimensional “traveling wave” results we employed in the rare-specialists regime will no longer apply. By extending our earlier calculations to the frequent-specialists regime, we will confirm that tradeoffs continue to be favored even when they are common, and demonstrate their impact on collective properties like the population-wide rate of adaptation. As before, we will start with periodic environments $\left(\sigma_{\tau }=0\right)$ and then discuss stochastically varying ones.

When mutations with different environmental fitness effects are widely available, the population as a whole will have a fully two-dimensional fitness distribution which is responsive to environmental change. Likewise, the descendants of a focal lineage will follow many different “mutational paths” depending on which types of mutations they acquire at each time. Despite this complexity, we can make progress by realizing that *successful* lineages (those that eventually reach fixation) are likely to follow an optimized mutational path which depends on environmental change. In other words, selection favors the organisms that happened to acquire the right mutations at the right times, even though each individual mutation arises randomly. To uncover the impact of frequent specialist mutations, we will derive this optimal mutational path and self-consistently relate it to the entire fitness distribution of the population.

#### Treatment of frequent tradeoffs.

In order to describe competition between a focal lineage and the rest of the population, we must make an ansatz for the dynamics of the bulk population in two-dimensional fitness space. We choose to parametrize fitness in terms of geometric mean fitness $X_{\|}$ and environmental “specialism” $X_{⊥}$, defined such that a mutation with fitness effect $\left(\overset{‾}{s},\Delta s\right)$ shifts $X_{\|}$ by $\overset{‾}{s}$ and $X_{⊥}$ by Δs. We similarly define relative fitnesses $x_{\|}\equiv X_{\|}-{\overset{‾}{X}}_{\|}$ and $x_{⊥}\equiv X_{⊥}-{\overset{‾}{X}}_{⊥}$, where bars represent the population mean. In the rare-specialists case we studied earlier, the bulk population was insensitive to the environment $\left({\overset{‾}{X}}_{⊥}=0\right)$ and had an average fitness which increased at rate v, such that ${\overset{‾}{X}}_{\|}\sim vt$. For the frequent-specialists regime, we make a similar linear ansatz:

(15a) $${\overset{‾}{X}}_{\|}\sim v_{\|}t,$$

(15b) $${\overset{‾}{X}}_{⊥}\sim I(t)\cdot v_{⊥}t.$$

where I(t)=±1 once again represents the current environmental state. Under this ansatz, the population zigzags in two-dimensional fitness space, increasing its long-term average fitness at the rate of adaptation $v_{\|}$, but also cyclically specializing to the current environment at the “rate of specialization” $v_{⊥}$ (Fig. 4A). The structure of this linear ansatz is in good agreement with simulations (Fig. S3A), but both rates $v_{\|}$ and $v_{⊥}$ must be determined self-consistently from the behavior of individual lineages.

To approach this complicated regime in the simplest possible way, we continue to employ our three-class JDFE containing generalist and specialist mutations, and assume that these mutations have the same geometric mean fitness effects $s\equiv \overset{‾}{s}=s_{g}$ and mutation rates $U_{g}=U_{s}$ (see SI 3.2.7 for the asymmetric generalization). We also specialize to the slower-cycling case $\tau =\tau_{1,2}\gg s/v_{\|}$. Finally, for the frequent-specialists regime we make a new assumption that s>Δs, so specialist mutations are always beneficial regardless of the environment. In the alternate regime Δs>s, many qualitative conclusions will hold (indeed, many of our simulation results are in this regime), but the optimal mutational path changes (SI 3.2.8). Finally, we note that the critical fitness scale $x_{c}$ can be redefined in terms of $v_{\|}$ according to Eq. (S60).

Now, we are equipped to generalize our rolling fitness recursion relations in Eq. (2) to account for acquisition of further specialist mutations:

(16a) $$x_{\|}(k+1)\approx x_{\|}(k)+s-v_{\|}T(k),$$

(16b) $$x_{⊥}(k+1)\approx x_{⊥}(k)+\Delta s\cdot D_{k}-v_{⊥}T(k)\cdot I(t(k)),$$

(16c) t(k+1)≈t(k)+T(k).

The identity of the mutations the focal lineage acquires is given by $D_{k}\in (-1,0,1)$, with 0 representing generalists and ±1 representing each specialist type. These equations are valid when t(k+1) and t(k) are both in the same environmental state, consistent with $\tau \gg s/v_{\|}$. They reduce to the rare-specialists case with constant $x_{⊥}$ when $v_{⊥}=D_{k}=0$.

#### Optimal mutational path.

Because the waiting time T(k) depends on $x_{\|}(k)$, $x_{⊥}(k)$ and t(k), the rolling fitness $x_{\|}(k)$ will depend on the mutational history of the lineage up to that point, i.e. the $D_{i}$ with i<k. We can write a solution for $x_{\|}(n)$ and $x_{⊥}(n)$ that generalizes Eq. (5) to a specializing lineage, valid when the effects of specialism $x_{⊥}$ can be treated perturbatively relative to $x_{\|}$:

(17a) $$x_{\|}(n,D)\approx x_{c}+e^{ns/x_{c}}\left[x_{\|}(0)-x_{c}+\zeta (D)\right],$$

(17b) $$x_{⊥}(n,D)\approx x_{⊥}(0)+\Delta s\sum_{i=0}^{n-1}D_{i}-\frac{v_{⊥}s}{v_{\|}}\sum_{i=0}^{n-1}I(t(i)),$$

(17c) $$\zeta (D)\equiv \frac{s}{x_{c}}\sum_{i=0}^{n-1}e^{-(i+1)s/x_{c}}\left(x_{⊥}(i,D)\cdot I(t(i))-\frac{sv_{⊥}}{2v_{\|}}\right).$$

Here, ζ is an effective fitness shift due to the net effect of specialization in the focal lineage and the rest of the population, which depends on the mutational path D through $x_{⊥}(i,D)$. The optimal mutational path $D^{*}$ maximizes ζ(D), and can be expressed as the sign of a weighted sum of environmental states after t(j):

(18a) $$D_{j}^{*}(n)\approx \text{sign}\left[\sum_{i=j+1}^{n-1}e^{-(i+1)s/x_{c}}I(t(i))\right],$$

(18b) $$D_{j}^{*}(n\to \infty )\approx \text{sign}\left[\frac{\tau }{2}-\text{min}(t(j),2\tau -t(j))\right]$$

for $n≳j+x_{c}/s$, measuring times modulo 2τ. The fitness boost for a lineage following this optimal path is

(19) $$\zeta^{*}\left(x_{⊥}^{0},t_{0}\right)\equiv \underset{n\to \infty }{\text{lim}}\zeta \left(D^{*},x_{⊥}^{0},t_{0}\right),$$

explicitly calculated in Eq. (S68) (SI 3.2.2).

The long-term optimal path in Eq. (18b) exactly matches our intuition from the rare-specialists case in Eq. (6b): specialist mutations aligned with the current environment are optimal only until it is halfway over. Afterward, a lineage should switch to the opposite type of specialist mutation to “pre-adapt” for the upcoming environment. This optimal behavior is phase-shifted by τ/2 generations relative to the specialization of the bulk population, which only adapts to the current environment – meaning that successful lineages are significantly more pre-adapted than typical ones (Fig. 4B; Fig. S4). Signatures of this pre-adaptation are also visible in the trajectories of individual mutations (Fig. 1D), where successful lineages often rise in frequency shortly after environmental switches – regardless of which environmental state has just started.

If the symmetry condition is relaxed so that environment-sensitive mutations have a smaller mutation rate or average fitness effect relative to generalist mutations, there will be a period roughly halfway into each environmental state where the optimal mutational path includes generalist mutations with D=0 (SI 3.2.7). Extreme differences in mutation rate of order $U_{g}/U_{s}\sim e^{\Delta s\cdot \tau /2}$ mark the transition between the rare-specialist and frequent-specialists regimes; more modest differences will largely be similar to the symmetric case.

#### Fixation probability and specialist advantage.

As in Eq. (8), we can estimate the fixation probability of a focal mutation with fitness effect $\left(\overset{‾}{s},\delta s\right)$ by integrating over the distribution of genetic backgrounds, which is now two-dimensional. (δs represents the tradeoff strength of the focal mutation: 0 for a generalist and ±Δs for a typical specialist mutation.)

(20) $$p_{\text{fix}}\left(\overset{‾}{s},\delta s|t_{0}\right)=\iint f\left(x_{\|},x_{⊥}\right)\cdot p_{\text{fix}}\left(\overset{‾}{s},\delta s|t_{0},x_{\|},x_{⊥}\right)dx_{\|}dx_{⊥}.$$

We can calculate the background distribution by estimating the effective fitness cost of deviating from the optimal path (SI 3.2.3), finding a two-dimensional Gaussian (Fig. S5):

(21) $$f\left(x_{\|},x_{⊥}\right)\approx \frac{1}{\sqrt{2\pi v_{\|}v_{⊥}}}e^{-x_{\|}^{2}/2v_{\|}-x_{⊥}^{2}/2v_{⊥}}.$$

The integral in Eq. (20) will be dominated by a narrow range of genetic backgrounds where the mutation lands at a critical fitness scale, $x_{\|}+\overset{‾}{s}+\zeta^{*}\left(x_{⊥}+\delta s,t_{0}\right)\approx x_{c}$ (Fig. S5, black curve); this favors values of $x_{⊥}$ which are pre-adapted to the upcoming environment (Fig. S5, pink dots). If the focal mutation is a specialist with δs≠0, it effectively shifts $x_{⊥}$, allowing the lineage to pre-adapt further. Thus, well-timed specialist mutations have a fixation advantage over generalists for the same reason as before: they provide an effective fitness boost which enables them to fix even on backgrounds with slightly lower average fitness.

The overall time dependence of Eq. (20) is somewhat more complicated than the rare-specialist regime, because the focal mutation must compete against environment-sensitive mutations on other genetic backgrounds (SI 3.2.5). Because these competing mutations will have an advantage if they arise at the start of a favorable environment, generalist mutations with δs=0 will be most likely to fix if they arise near the middle of an environmental state, where specialists have no advantage (Fig. S6A). While this effect attenuates the overall specialist advantage $p_{\text{fix}}(\overset{‾}{s},\Delta s)/p_{\text{fix}}(\overset{‾}{s},0)$ to scale linearly with Δs⋅τ rather than exponentially (Fig. S7A), mutations with tradeoffs are still strongly favored overall.

When tradeoffs are strong enough that specialist mutations appear deleterious in their unfavored environment (Δs>s), the optimal mutational path shifts so that successful lineages *stop* acquiring mutations in the middle of each environmental state, then acquire “bursts” of specialist mutations in rapid succession near environmental switches (SI 3.2.8; Fig. S6B). Because specialist mutations are strongly favored by natural selection at these times, this shift coincides with the specialist advantage growing to scale with exp(Δs⋅τ), as in the rare-specialists regime (Fig. 1C, Fig. 4C, Fig. S7A).

#### Rates of specialization and adaptation.

We can estimate the rate of specialization $v_{⊥}$ by considering the “short-term” optimal mutational path for mutations shortly before the present (SI 3.2.3), yielding

(22) $$v_{⊥}\sim \frac{x_{c}\Delta s}{s\tau }.$$

As τ increases, the population maintains less steady-state diversity in environmental preference, lowering the rate of specialization $v_{⊥}$. This result holds qualitatively in simulations (Fig. S3B), but Eq. (22) tends to overestimate the measured $v_{⊥}$ except in extremely large populations where all of our asymptotic assumptions can be strongly satisfied.

We can estimate the long-term rate of adaptation $v_{\|}$ through the self-consistent relation

(23) $$v_{\|}=Ns\left[U_{s}\cdot p_{\text{fix}}(s,\pm \Delta s)+U_{g}\cdot p_{\text{fix}}(s,0)\right].$$

The fitness boost $\zeta^{*}$ from following the optimal mutational path will increase $p_{\text{fix}}$ and therefore $v_{\|}$ relative to the rare-specialist regime. To leading order (SI 3.2.6), this boost is

(24) $$\frac{v_{\|}-v_{0}}{v_{0}}\approx \frac{\Delta s}{3s}\frac{v_{0}\tau }{x_{c}}$$

where $v_{0}$ corresponds to v for a population with rare specialists (or τ→0). This small boost stems from the fact that specializing lineages will rise in frequency more quickly by adapting to the environment, making the rate of adaptation larger than the one predicted by average fitness alone (Fig. 5A). This boost is sensitive to our symmetry assumption that the JDFE contains specialist and generalist mutations with the same average fitness effect s. In fact, as we will discuss in the next section, $v_{\|}$ can fall below $v_{0}$ if specialist mutations provide a smaller average fitness benefit than generalists.

Despite the complexities of the frequent-specialists regime, we see that the key qualitative conclusion from the rare-specialists regime holds. Mutations with environmental tradeoffs have a significant fixation advantage over generalist mutations when they arise near the beginning of their favored environment, because transient fitness benefits have more time to compound if they occur soon after a lineage is founded. Because of this advantage, successful lineages will generically “pre-adapt,” acquiring a suite of opposing specialist mutations at times aligned with upcoming environmental states.

### Environmental stochasticity with multiple specializing lineages

When the environmental state varies stochastically $\left(\sigma_{\tau }>0\right)$ in a population with common specialist mutations $\left(NU_{s}\gg 1\right)$, the entire population will respond to stochastic environmental change. To roughly describe this adaptation, we will continue to use the linear ansatz in Eq. (15) described by the rate of adaptation $v_{\|}$ and the rate of specialization $v_{⊥}$. However, the environmental state I(t) is now a random variable that does not perfectly balance the time spent in each environment, so the population will have more time to specialize toward an environment that lasts longer.

#### Optimal mutational path.

The optimal mutational path will also depend on the realized environmental stochasticity – but unlike the bulk population, it will “anticipate” the environmental state that happens to be overrepresented in the near future. To formalize this, we take the fast-cycling $\tau \ll s/v_{\|}$ limit, where the relevant unit of environmental stochasticity is the environmental bias over a single founding step of time $s/v_{\|}\gg \tau$:

(25) $$\delta \tau_{j}^{\text{step}}\equiv \sum_{i=1+j\left(s/2v_{\|}\tau \right)}^{(j+1)\left(s/2v_{\|}\tau \right)}\delta \tau_{i},$$

with $\delta \tau_{i}$ the time difference between adjacent environmental states. Solving the rolling fitness relations (SI 3.3) reveals that the optimal mutational path is given by a weighted sum of future environmental stochasticity:

(26) $$D_{j}^{*}(n,\delta \tau )\approx \text{sign}\left[\sum_{i=j+1}^{n-1}e^{s(i-j)/x_{c}}\cdot \delta \tau_{i}^{\text{step}}\right].$$

A lineage which follows this optimal mutational path will acquire an effective fitness boost of $\xi^{*}\left(x_{⊥}^{0},\delta \tau \right)$, playing a similar role to $\zeta^{*}$ in Eq. (19). A focal lineage will be likely to fix if its starting fitness satisfies $x_{\|}^{0}+\xi^{*}\left(x_{⊥}^{0},\delta \tau \right)≳x_{c}$. However, the fitness shift $\xi^{*}$ has a complicated dependence on the environmental stochasticity δτ, since it encodes the competition between an optimally-specializing lineage and the background population. Thus, in order to average over environmental stochasticity and estimate the overall fixation probability, we employ a series of rough semianalytic approximations to the distribution of $\xi^{*}$ rather than solving it exactly (SI 3.3.1).

#### Specialist advantage.

When specialist mutations are widely available, the specialist advantage $p_{\text{fix}}(\overset{‾}{s},\Delta s)/p_{\text{fix}}(\overset{‾}{s},0)$ in stochastically fluctuating environments continues to be much greater than unity (Fig. 4C). This advantage arises from the contribution of a *single* specialist mutation to a lineage following the optimal mutational path, effectively shifting $x_{⊥}^{0}$ to $x_{⊥}^{0}+\Delta s$. This shift widens the distribution of $\xi^{*}$ toward higher fitness values (Fig. S8), making it easier for mutations to reach fixation.

However, the specialist advantage does not attain the extreme values of ~10^6^ and larger we observed in the rare-specialists regime (Fig. 3B–C; Fig. S7B). This is because the fates of specialist and generalist mutations alike are both sensitive to environmental stochasticity, due to linkage and competition with other specialist mutations. When the entire population is able to specialize at rate $v_{⊥}$, a single lineage will not be able to take sole advantage of a long stretch of beneficial environments: rather, it will compete with many other specialist mutations on other genetic backgrounds that are also rising in frequency, cutting off the benefit stochasticity can provide to the focal lineage.

#### Rate of adaptation.

Because successful lineages will acquire mutations that match future environmental stochasticity, they will rise in frequency more quickly and raise the overall rate of adaptation. We can estimate this by determining how typical values of $\xi^{*}$ feed back on the rate of adaptation through Eq. (S167), finding

(27) $$\frac{v_{\|}-v_{0}}{v_{0}}\sim \frac{\sigma_{\tau }}{\tau }\frac{\Delta s}{s}\sqrt{\frac{v_{0}\tau }{x_{c}}}.$$

While this adjustment is nominally small, for large enough Δs and $\sigma_{\tau }$ it can raise the population rate of adaptation by a factor of two (Fig. 5B). As in the case without seasonal drift, Eq. (27) assumes that specialist and generalist mutations provide the same average benefit s. If specialists have a smaller average fitness effect than generalists, this boost will decline and can become negative, when natural selection favors specialist mutations despite the fact that they contribute less to long-term fitness gain. Thus, the presence of specialist mutations can speed up or slow down the rate of adaptation depending on the shape of the underlying JDFE (Fig. S9).

#### Rate of specialization.

Finally, we can estimate the rate of specialization $v_{⊥}$ by tracking how the optimal mutational path depends on the realized stochasticity (SI 3.3.3):

(28) $$v_{⊥}\sim \frac{\Delta s}{s\sigma_{\tau }}\sqrt{v_{\|}\tau x_{c}}.$$

We see that $v_{⊥}$ declines with the stochasticity $\sigma_{\tau }$, mirroring Eq. (22), which shows that $v_{⊥}$ declines with τ in deterministic environments. Intuitively, environmental stochasticity reduces the diversity in the population by creating long stretches where one environment is favored over another, lowering the rate of specialization (Fig. S10). Thus, the rate of specialization is maximized for perfectly periodic environments with small τ – the same conditions where tradeoffs become effectively neutral.

Our results in this section continue to roughly apply in slower-cycling environments with $\tau \gg s/v_{\|}$ (SI 3.3.2). In principle, for larger τ the optimal mutational path will depend on a combination of stochasticity and the perfectly-periodic effects discussed in the previous section. However, we find that focusing on stochasticity alone is reasonable if $\sigma_{\tau }$ is sufficiently large.

## Discussion

Existing theory struggles to predict how natural selection contends with varying environmental conditions, when beneficial mutations with different fitness tradeoffs compete. To address this issue, we modeled a population in a two-state environment whose members acquire linked mutations, according to a simple joint distribution of fitness effects (JDFE). We found that competition between linked mutations systematically selects for environmental tradeoffs (Fig. 6), providing environment-sensitive mutations a fixation advantage over environment-insensitive ones with larger geometric mean fitness effects – in contrast to past work where mutations accumulate one-at-a-time (16–21). This “specialist advantage” is present because a mutant lineage which arises in a favorable environment can found further beneficial mutations sooner, providing a long-term advantage even after the environment changes. The specialist advantage is most evident in the strong-tradeoff regime when the environmental fluctuation timescale is long (Δs⋅τ≫1) or sufficiently stochastic $\left(\Delta s\cdot \sigma_{\tau }\gg \sqrt{v\tau /x_{c}}\right)$, and applies whether or not environment-sensitive mutations are rare or common in the JDFE.

Our results demonstrate how the fitness effects and tradeoffs of *successful* mutations depend on environmental fluctuations and the underlying JDFE, quantities which can be measured or manipulated in evolution experiments (9, 22, 26). Renewable barcoding systems are particularly potent experimental tools to study rapidly adapting populations, because they allow beneficial mutations to be detected while they are still at low frequencies (14, 26). Applying these methods to fluctuating environments would test our prediction that as the fluctuation timescale increases (for a fixed JDFE), natural selection will increasingly filter for strong-tradeoff mutations that arise near the start of favorable environments.

When environment-sensitive mutations are common, clonal interference between mutations with different tradeoff profiles dramatically reshapes the outcome of natural selection. By tracking how the fate of a lineage depends on the joint fitness effects of the entire sequence of mutations it acquires, we found that successful lineages generically pre-adapt to upcoming environmental transitions, by acquiring well-timed mutations with strong environmental tradeoffs. This alignment between tradeoffs and environmental change allows beneficial mutations to rise in frequency more quickly, speeding up the rate of adaptation of the population as a whole. However, environment-sensitive mutations can also slow down adaptation, by outcompeting constitutively beneficial mutations with higher geometric mean fitness. Thus, natural selection does not always optimize long-term fitness gain, and the rate of adaptation is sensitive to the shape of the two-dimensional JDFE.

In this work, we limited our analysis to a two-state environment, allowing us to focus on a single axis of environmental tradeoffs. While this approach could be considered a rough model of seasonal cycles (44) or experimental evolution protocols (9, 11), natural populations can experience a much larger number of potential environmental states. We conjecture that many of our conclusions should naturally extend to a broader set of environments: the fate of a mutation is determined by its fitness across each environment it encounters on its way to fixation, with more weight placed on early environments. On the population level, rapid adaptation constitutes a strategy to respond to environmental change, comparable to bet-hedging by stochastically switching between phenotypes (45). This evolutionary strategy is likely to be favored when environments change over many generations (46), relative to strategies like genetic regulation (47, 48) which allow for phenotypic flexibility on shorter timescales, but may carry fitness costs (49, 50).

Our work focuses on a simplified point JDFE that neglects epistatic interactions between mutations. In reality, the fitness effects of mutations depend not only on the environmental state but also on the mutation’s genetic background. While these epistatic fitness landscapes are beyond the scope of this work, recent work has begun to characterize their environmental dependence empirically (10). The structure of these landscapes critically determines whether long-term evolution tends to alleviate the effects of tradeoffs (51, 52), or sharpen them in the form of a Pareto front at the “boundary” of mutational space, where any increase in fitness requires a corresponding cost in another environment (53). Our results demonstrate that successful mutations can still carry strong tradeoffs even when unconditionally beneficial mutations are available, far from a hypothetical Pareto front. This circumstance is plausible for an organism which has recently migrated to a new environment, or for experimentally measured fitness effects in non-natural environments (9, 11).

Finally, a key limitation of this work was ignoring recombination between genomes, which has the potential to break up the linkage between alleles that underlies many of our results. Recombination has been proposed to be an important mechanism of adaptation in fluctuating environments, providing a gene pool of environment-specific alleles that allow for modularity under changing environmental conditions (54, 55). Understanding how these factors conspire to shape long-term evolutionary outcomes in fluctuating environments is a key direction for future work.

## Supplementary Material

1

## Figures and Tables

**Figure 1:**
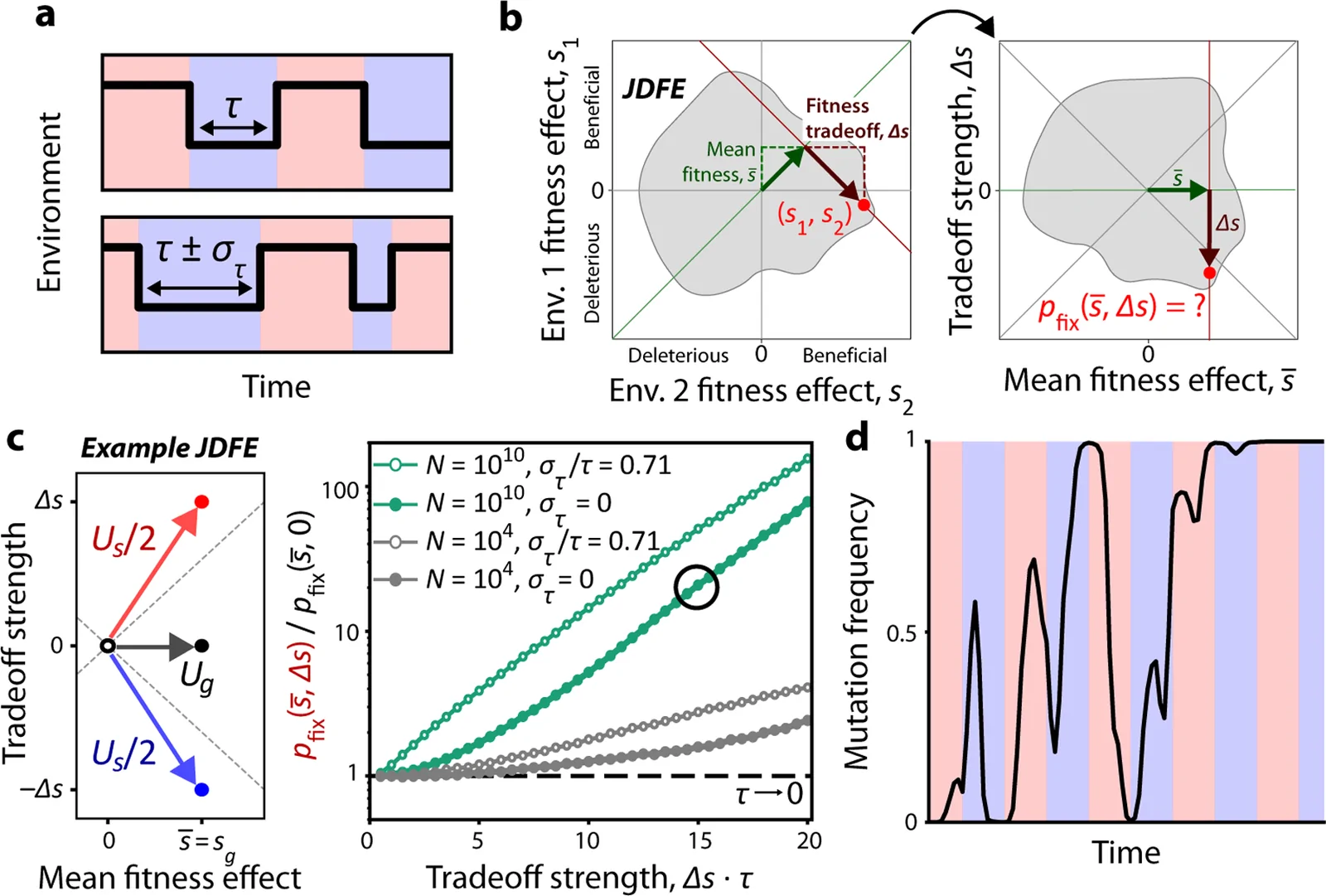
Mutations with tradeoffs are favored in a rapidly adapting population. **(a)** We consider a two-state environment where each state lasts τ generations. The switching can either be perfectly periodic (top) or have stochasticity $\sigma_{\tau }$ (bottom). **(b)** The mutational landscape in a two-state environment can be described by a two-dimensional joint distribution of fitness effects (JDFE). We reparametrize these fitness effects into geometric mean fitness $\overset{‾}{s}$ and tradeoff strength Δs, then aim to calculate the fixation probability as a function of these parameters. **(c)** Left: An example coarse-grained JDFE where mutations are grouped into generalist and specialist types that arise at rates $U_{g}$ and $U_{s}$, respectively. Generalist mutations have the same benefit $s_{g}$ in each environment. Specialist mutations have a geometric mean fitness effect $\overset{‾}{s}$ (which in this case, is equal to $s_{g}$) and an additional tradeoff Δs. Right: We simulated evolution under this JDFE, and measured the specialist advantage $p_{\text{fix}}(\overset{‾}{s},\Delta s)/p_{\text{fix}}(\overset{‾}{s},0)$ as a function of tradeoff strength Δs. Results are shown for the rapid adaptation regime $\left(N=10^{10}\right)$ and the successive mutations regime $\left(N=10^{4}\right)$, at different values of switching stochasticity $\sigma_{\tau }$. Dashed line shows the τ→0 prediction where the fixation probability depends only on $\overset{‾}{s}$. Simulations were run with $s_{g}=\overset{‾}{s}=0.03$, $U_{g}=U_{s}=3\cdot 10^{-6}$, and τ=100. **(d)** Sample trajectory of a successful specialist mutation (favoring the red environment) for the parameters corresponding to the circled point in (c).

**Figure 2:**
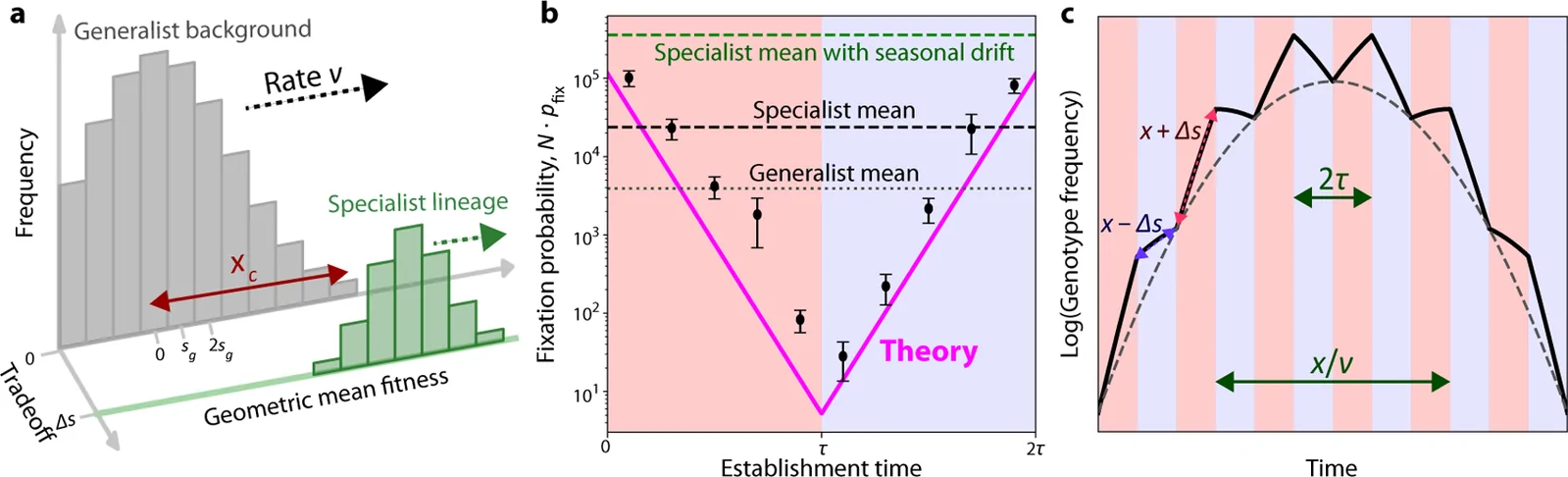
Traveling fitness waves with rare environmental tradeoffs. **(a)** Schematic of the two-dimensional fitness distribution of a population with rare specialist mutations. The background population forms a traveling wave (gray) insensitive to environmental change, increasing its fitness at rate v by acquiring generalist mutations with fitness effect $s_{g}$. A small specialist lineage (green) descends from a mutation with an environmental tradeoff Δs, and can acquire further generalist mutations that advance its geometric mean fitness. **(b)** The fixation probability of a rare specialist mutation as a function of the time when it arises, within a beneficial (red) or deleterious (blue) environment. Dashed line shows the specialist fixation probability averaged over time while dotted line shows the fixation probability of a generalist mutation. The green line shows the fixation probability of the same specialist when the environment has a modest amount of seasonal drift, $\sigma_{\tau }/\tau =0.35$. Pink line is the theoretical prediction from Eq. (9). Error bars show SEM (see SI 4.2). Simulations were run with $N=10^{10}$, $\overset{‾}{s}=s_{g}=0.03$, Δs=0.1, $U_{g}=3\cdot 10^{-6}$, and τ=100. **(c)** A schematic of the expected trajectory of a specialist genotype that arises with geometric mean fitness x, in an environment which fluctuates with timescale τ (shading). Over timescales ~x/v, this genotype will be outcompeted by fitter ones, which may be this genotype’s descendants or those of another strain in the background population. The dashed line shows the trajectory of an environment-insensitive genotype with the same geometric mean fitness.

**Figure 3:**
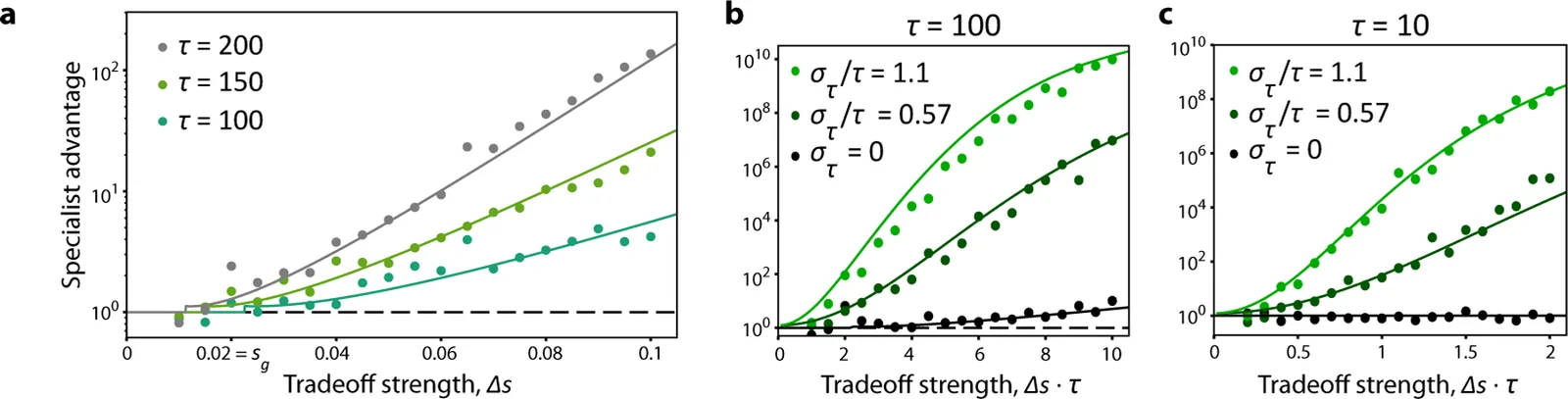
Fixation probabilities of rare mutations with environmental tradeoffs. **(a)** Fixation advantage of rare environment-sensitive specialists, $p_{\text{fix}}(\overset{‾}{s},\Delta s)/p_{\text{fix}}(\overset{‾}{s},0)$, in a perfectly periodic environment $\left(\sigma_{\tau }=0\right)$ for different environmental fluctuation times τ and tradeoff strengths Δs. Curves show theory prediction from Eq. (S31) and Eq. (S32), and unity for Δs⋅τ<2.25 (where the specialist advantage is negligible). **(b)** The specialist advantage in a stochastic environment with τ=100, for different values of $\sigma_{\tau }$ and Δs. Curves show theory prediction obtained from numerically integrating Eq. (S46) under the normal approximation in Eq. (S47) with no cutoff c. **(c)** Same as (b), but in a rapidly fluctuating environment with τ=10. Simulations were run with $N=10^{20}$, $s_{g}=\overset{‾}{s}=0.02$, and $U_{g}=3\cdot 10^{-9}$.

**Figure 4:**
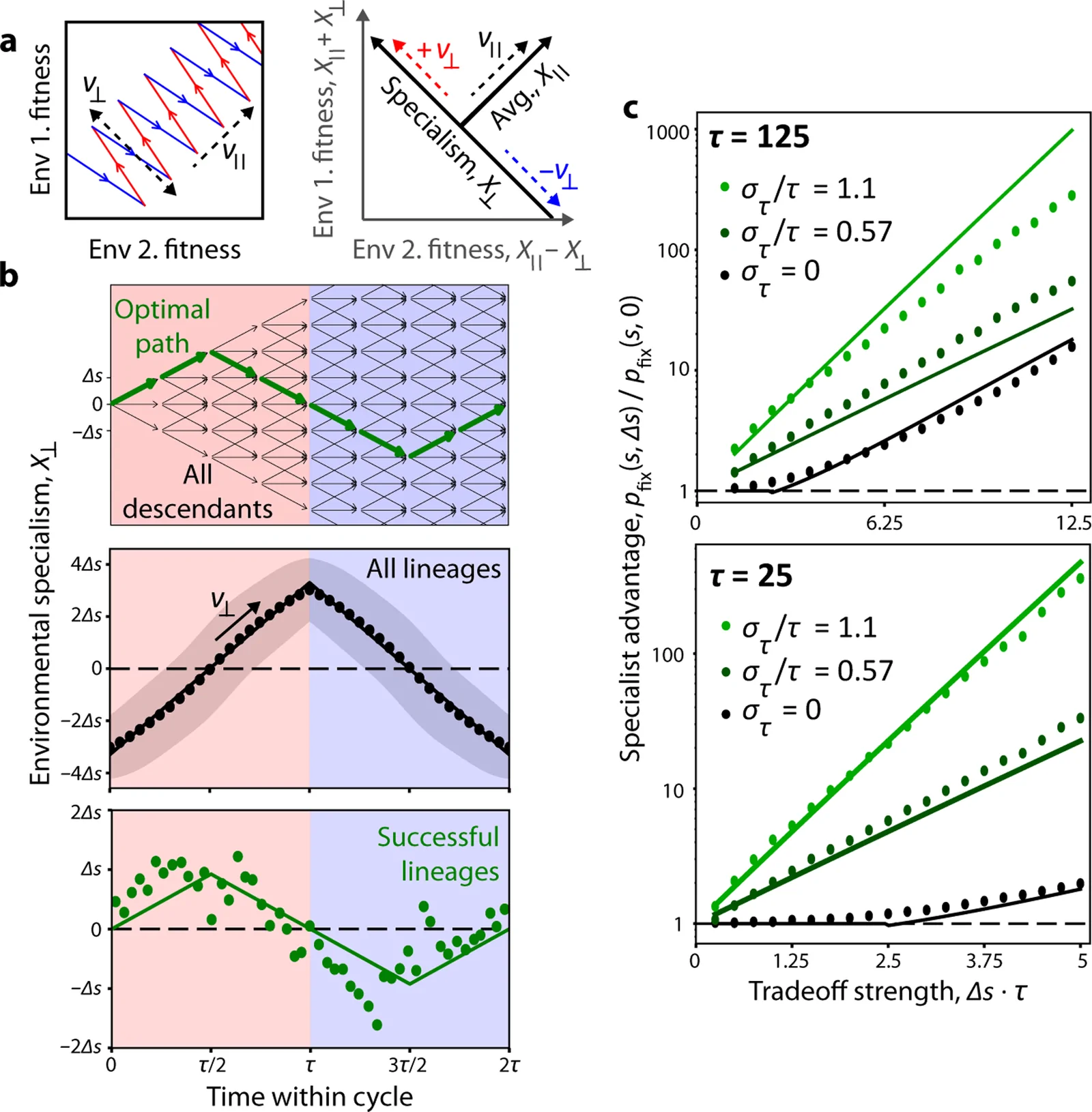
Populations with frequent specialist mutations follow an optimal mutational path. **(a)** Left: When specialist mutations are frequent, the population mean zigzags in two-dimensional fitness space, adapting at rate $v_{\|}$ over the long term, but cyclically specializing to the current environment at rate $v_{⊥}$. Right: We parametrize fitness as average fitness $X_{\|}$ and environmental specialism $X_{⊥}$, corresponding to $v_{\|}$ and $v_{⊥}$. **(b)** Top: Schematic of the environmental specialism of the members of a lineage, with mutations represented by arrows. The descendants with the largest long-term growth rate follow the “optimal mutational path” shown by the green arrow. Middle: The average environmental specialism ${\overset{‾}{X}}_{⊥}$ over an environmental cycle. The line is a best-fit line to simulation results (points); the shading shows a standard deviation. Bottom: The empirical environmental specialism $X_{⊥}$ of new mutations (accounting for their genetic background) binned by establishment time, conditioned on those mutations later reaching fixation. The line shows a best-fit line consistent with the optimal mutational path. **(c)** Specialist advantage $p_{\text{fix}}(s,\Delta s)/p_{\text{fix}}(s,0)$ of frequent specialist mutations, for various values of Δs, τ, and $\sigma_{\tau }$. Theory curves show prediction from Eq. (S135) when $\sigma_{\tau }=0$, and from Eq. (S165) when $\sigma_{\tau }>0$, using values of $v_{\|}$ fit from simulations. Simulations run with $N=10^{20}$, $U_{g}=U_{s}=3\cdot 10^{-9}$, s=0.02, Δs=0.05, and τ=200 unless otherwise noted.

**Figure 5:**
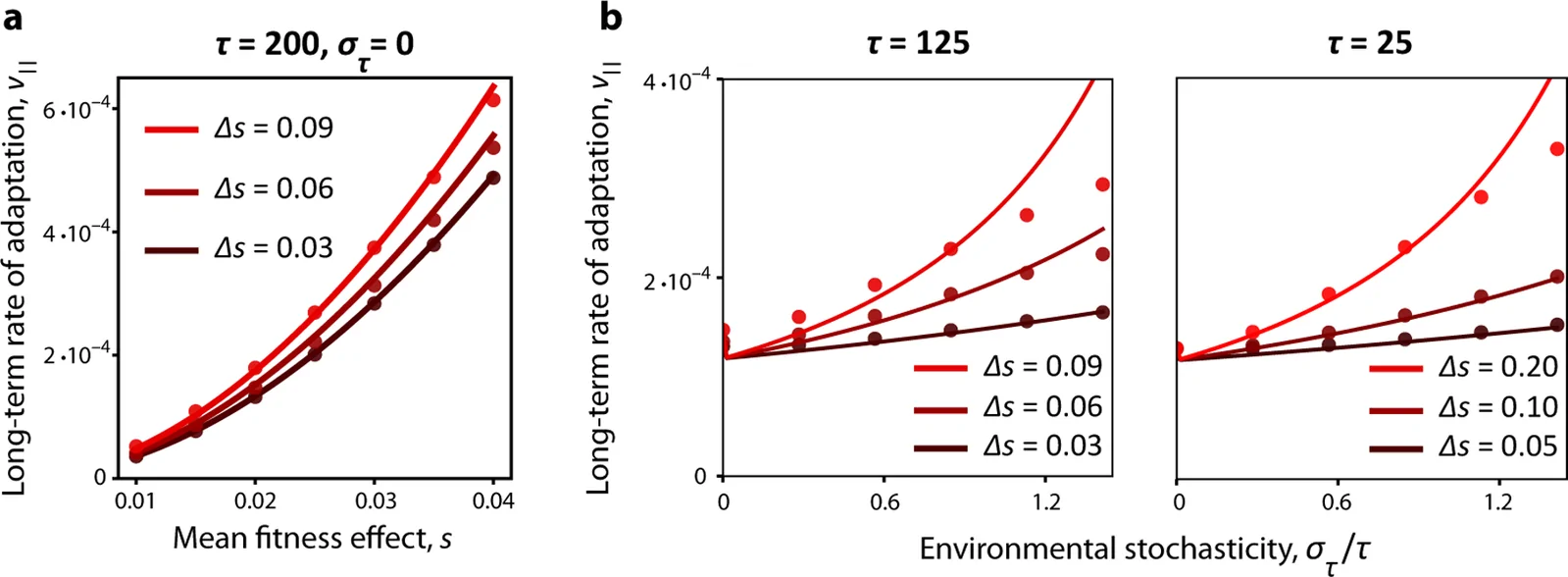
Environmental fluctuations increase the population’s overall rate of adaptation. **(a)** Rate of adaptation $v_{\|}$ as a function of the mean fitness benefit s of mutations, for different specialist environmental tradeoffs Δs in a deterministically switching environment $\left(\sigma_{\tau }=0\right)$. Curves show theory prediction, numerically solved from Eq. (S106). **(b)** The rate of adaptation as a function of environmental stochasticity $\sigma_{\tau }$ for different values of Δs and τ. Curves show theory prediction from solving Eq. (S167), compared to values of $v_{⊥}$ measured from simulations. Simulations used s=0.02, $N=10^{20}$, and $U_{g}=U_{s}=3\cdot 10^{-9}$ unless otherwise noted.

**Figure 6:**
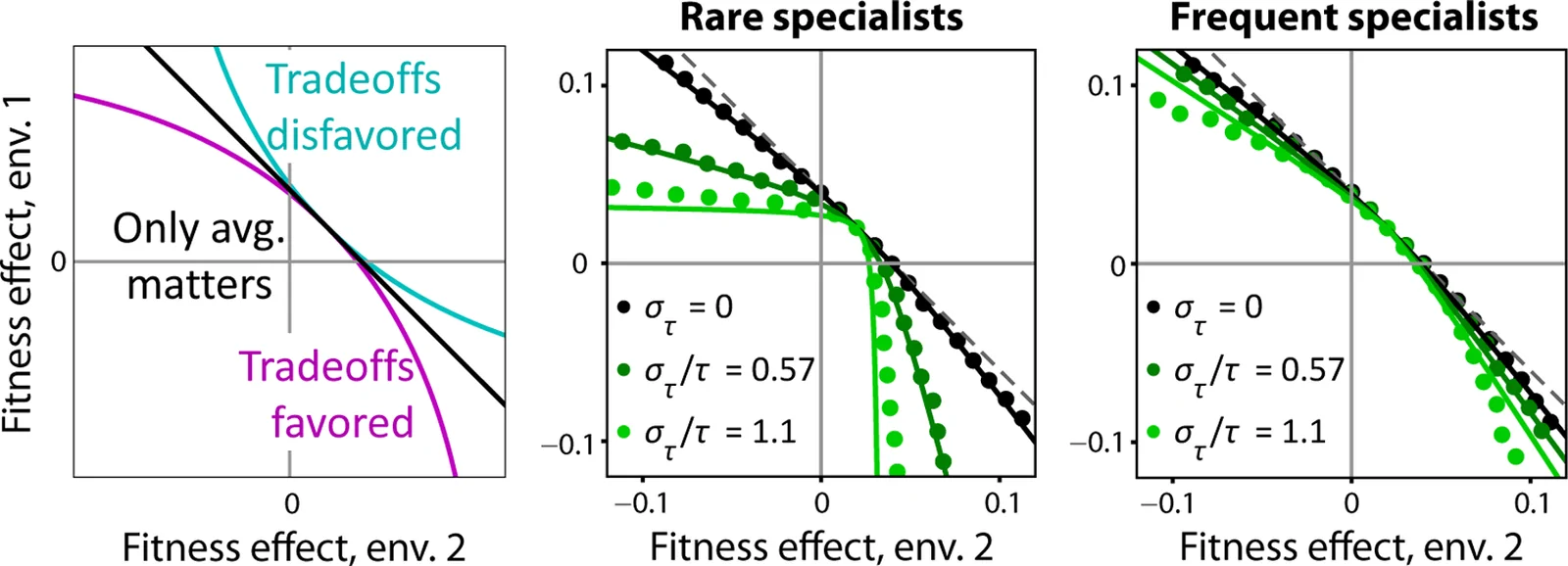
Natural selection favors environmental tradeoffs when linked mutations compete. Fixation probability isoclines (as described in SI 4.3) in two-dimensional fitness space, with fixation probability equal to $p_{\text{fix}}(s,0)$. Plots show a schematic (left) and results for simulations with τ=200 with rare and frequent specialist mutations (center and right). Dashed lines show the “average only” curve in the schematic, equivalent to τ→0 and $\sigma_{\tau }/\tau \to 0$. Solid lines show theory predictions. Parameters are the same as Fig. 4B unless otherwise noted.

## Data Availability

Code for simulations and figure generation is available on GitHub (github.com/jdmcenany/Fluctuating_Environments). Repository of precomputed simulation results is available on Zenodo (56).
