# Supplementary material for: Rapid evolution can select for fitness tradeoffs in fluctuating environments
Source: bioRxiv. 2026 Sep 4:2026.09.01.748641. Preprint. [Version 1] doi: 10.64898/2026.09.01.748641 (PMC13684556; doi:10.64898/2026.09.01.748641)
Supplement: Supplementary file 1 [file NIHPP2026.09.01.748641V1-supplement-1.pdf]

bioRxiv preprint doi: https://doi.org/10.64898/2026.09.01.748641; this version posted September 4, 2026. The copyright holder for this preprint (which was not certified by peer review) is the author/funder, who has granted bioRxiv a license to display the preprint in perpetuity. It is made available under a CC-BY 4.0 International license.

Supplementary Information

Contents

Page

1 Model Overview **13**
1.1 Clonal Interference Regime . . . 13
1.2 Mutations in a Fluctuating Environment . . . 14

2 Fixation Probability of a Rare Specialist **15**
2.1 Fixation in a Deterministic Environment . . . 16
2.1.1 Successive Establishments and Rolling Fitness . . . 16
2.1.2 Long-term Rolling Fitness with Environmental Tradeoffs . . . 17
2.1.3 Jackpot Mutations . . . 19
2.1.4 Overall Fixation Probability . . . 20
2.1.5 Asymmetric Temporal Fluctuations . . . 21
2.2 Fixation with Seasonal Drift . . . 22
2.2.1 Rolling Fitness with Seasonal Drift . . . 23
2.2.2 Higher-Variance Environments . . . 26

3 Dynamics with Frequent Specialists **27**
3.1 Introduction . . . 27
3.2 Frequent Specialists in a Periodic Environment . . . 29
3.2.1 Rolling Fitness of a Specializing Lineage . . . 29
3.2.2 Optimal Mutational Path . . . 30
3.2.3 Two-Dimensional Traveling Wave . . . 31
3.2.4 Jackpot Mutations in the Frequent-Specialist Regime . . . 34
3.2.5 Overall Fixation Probability . . . 36
3.2.6 Self-Consistent Rate of Adaptation . . . 38
3.2.7 Asymmetric Fitness Effects and Mutation Rates . . . 39
3.2.8 Stronger Tradeoffs and Specialist Bursting . . . 41
3.3 Frequent Specialists with Seasonal Drift . . . 44
3.3.1 Faster Environmental Cycles . . . 45
3.3.2 Slower Environmental Cycles . . . 50
3.3.3 Rate of Specialization . . . 51

4 Simulated Population Dynamics **52**
4.1 Overview . . . 52
4.2 Test Lineages . . . 53
4.3 Fixation Probability Isoclines . . . 55

bioRxiv preprint doi: https://doi.org/10.64898/2026.09.01.748641; this version posted September 4, 2026. The copyright holder for this preprint (which was not certified by peer review) is the author/funder, who has granted bioRxiv a license to display the preprint in perpetuity. It is made available under a CC-BY 4.0 International license.

List of Figures

S1 Typical stochastic fluctuations still provide a large advantage to specialists. 3
S2 Rare environment-sensitive mutations in high-variance environments. . . . 4
S3 The bulk population specializes toward the current environment. 5
S4 Optimal mutational path in asymptotically large populations. 6
S5 Two-dimensional fitness distribution in a population with frequent specialist mutations. 7
S6 Time-dependent fixation probabilities when specialist mutations are frequent. 8
S7 The specialist advantage depends on the mutation rate of specialist mutations. 9
S8 Characterizing the effect of seasonal drift on fixation in specializing populations. 10
S9 The rate of adaptation depends on the joint distribution of fitness effects. . . 11
S10 The rate of specialization declines in stochastic environments. 12

bioRxiv preprint doi: https://doi.org/10.64898/2026.09.01.748641; this version posted September 4, 2026. The copyright holder for this preprint (which was not certified by peer review) is the author/funder, who has granted bioRxiv a license to display the preprint in perpetuity. It is made available under a CC-BY 4.0 International license.

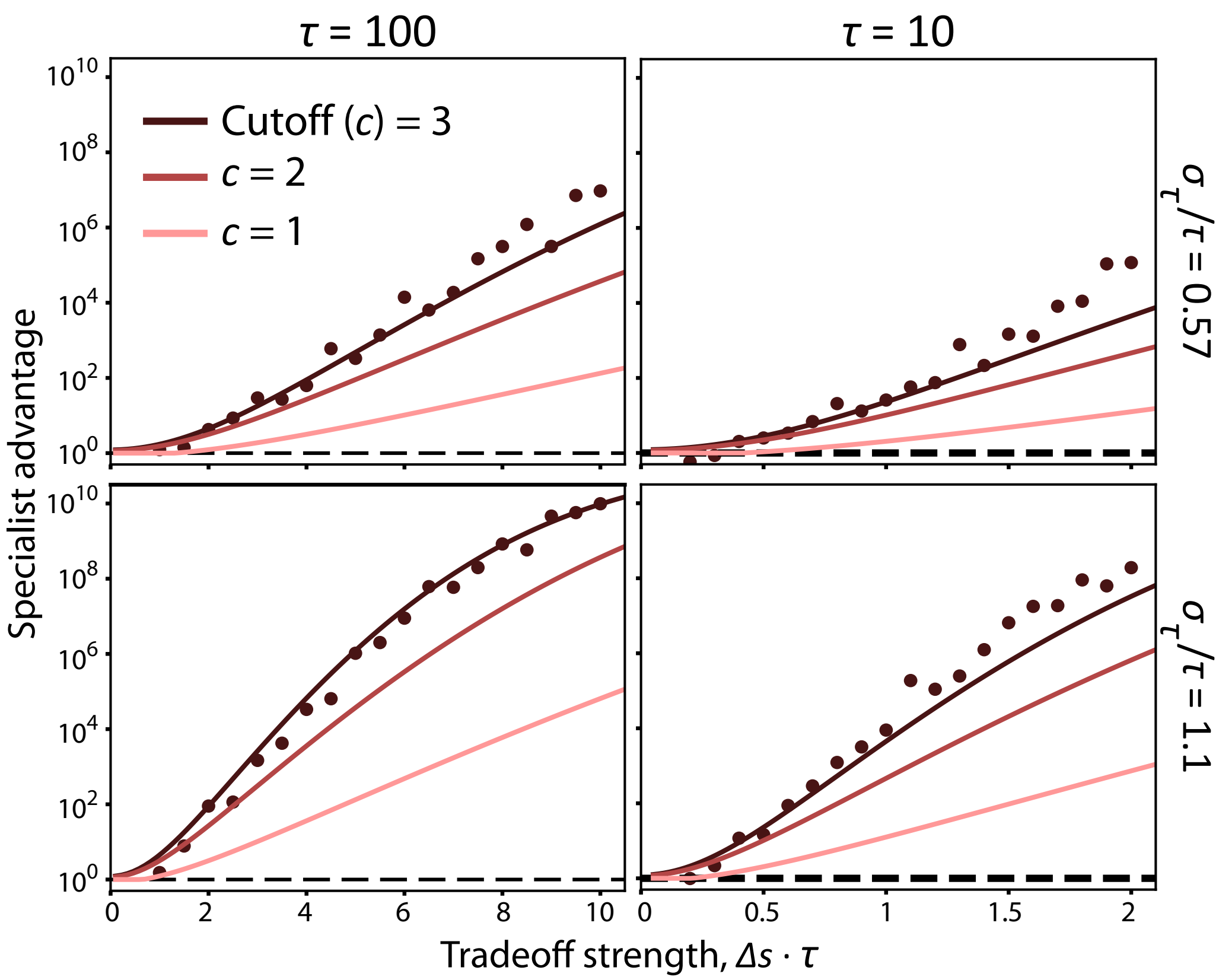

Figure S1: Typical stochastic fluctuations still provide a large advantage to specialists. Points show the specialist advantage $p_{\text{fix}}(\bar{s}, \Delta s)/p_{\text{fix}}(\bar{s}, 0)$ in a stochastic environment, for different values of τ, σ_τ and Δs. Curves show theory prediction as in Fig. 3B-C for different values of c, the cutoff (measured in standard deviations) imposed on stochastic fluctuations. Typical environmental fluctuations with $c \sim 1$ still have a large specialist advantage, although reduced from the full average captured over many independent simulations. Simulations were run with $N = 10^{20}$, $\bar{s} = s_g = 0.02$, and $U_g = 3 \cdot 10^{-9}$.

bioRxiv preprint doi: https://doi.org/10.64898/2026.09.01.748641; this version posted September 4, 2026. The copyright holder for this preprint (which was not certified by peer review) is the author/funder, who has granted bioRxiv a license to display the preprint in perpetuity. It is made available under a CC-BY 4.0 International license.

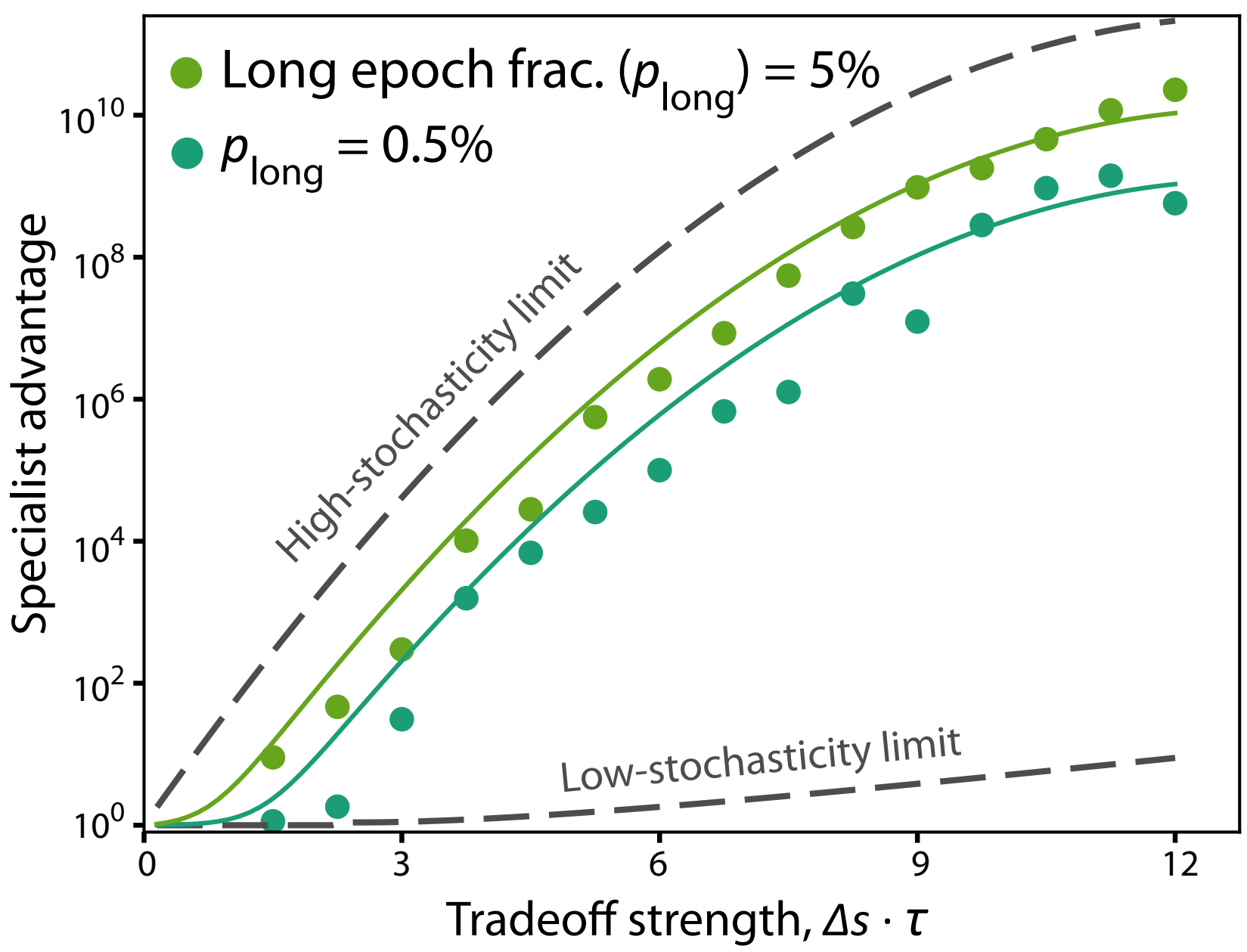

Figure S2: Rare environment-sensitive mutations in high-variance environments. Specialist advantage $p_{\text{fix}}(\bar{s}, \Delta s)/p_{\text{fix}}(\bar{s}, 0)$ in a high-variance environment where environmental states usually last $\tau = 150$ generations, but rarely last $\tau_{\text{long}} = 1000$ generations with probability p_{long}. Points show simulation results for two values of p_{long}. Bottom dashed line shows the low-stochasticity limit ($\sigma_\tau = 0$) from Eq. (S31) and Eq. (S32). Top dashed line shows the high-stochasticity limit, $p_{\text{fix}}(\bar{s} + \Delta s, 0)/p_{\text{fix}}(\bar{s}, 0)$. Theory curves show the low-stochasticity limit plus p_{long} times the high-stochasticity limit, which is dominated by the probability of a rare long epoch. Simulations were run with $N = 10^{20}$, $\bar{s} = s_g = 0.02$, and $U_g = 3 \cdot 10^{-9}$ with rare specialist mutations.

bioRxiv preprint doi: https://doi.org/10.64898/2026.09.01.748641; this version posted September 4, 2026. The copyright holder for this preprint (which was not certified by peer review) is the author/funder, who has granted bioRxiv a license to display the preprint in perpetuity. It is made available under a CC-BY 4.0 International license.

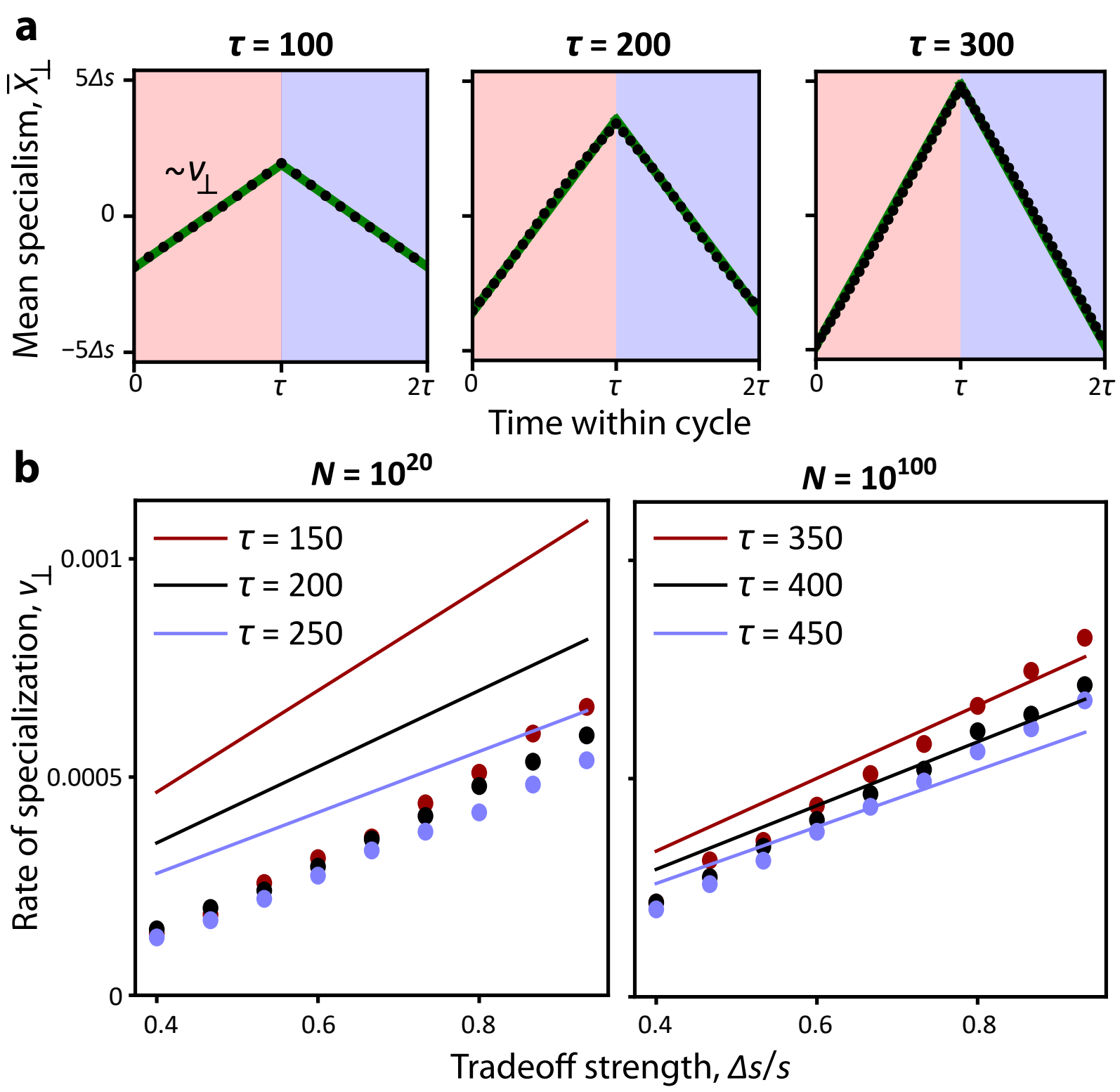

Figure S3: The bulk population specializes toward the current environment. (a) The mean specialism of the population $\overline{X}_\perp$ as a function of time within an environmental cycle, for different environmental cycling times τ. Points show simulation averages (measured relative to the start of an environmental cycle and normalized to have mean zero), while green curves are fit lines. The slope of these fit lines defines $v_\perp$. **(b)** Rate of specialization $v_\perp$ as a function of tradeoff strength Δs and environmental cycling time τ. Points represent simulation results, while theory curves are from Eq. (S71) (using the leading-order approximations for $v_\parallel$ and x_c from the rare-specialists regime). Note that the theory only agrees quantitatively with simulations when the population size is large enough to strongly satisfy all the regime assumptions. Parameters for (a) and (b), left: $N = 10^{20}$, $s = 0.015$, $U_g = U_s = 10^{-6}$. Parameters for (b), right: $N = 10^{100}$, $s = 0.015$, $U_g = U_s = 10^{-15}$.

bioRxiv preprint doi: https://doi.org/10.64898/2026.09.01.748641; this version posted September 4, 2026. The copyright holder for this preprint (which was not certified by peer review) is the author/funder, who has granted bioRxiv a license to display the preprint in perpetuity. It is made available under a CC-BY 4.0 International license.

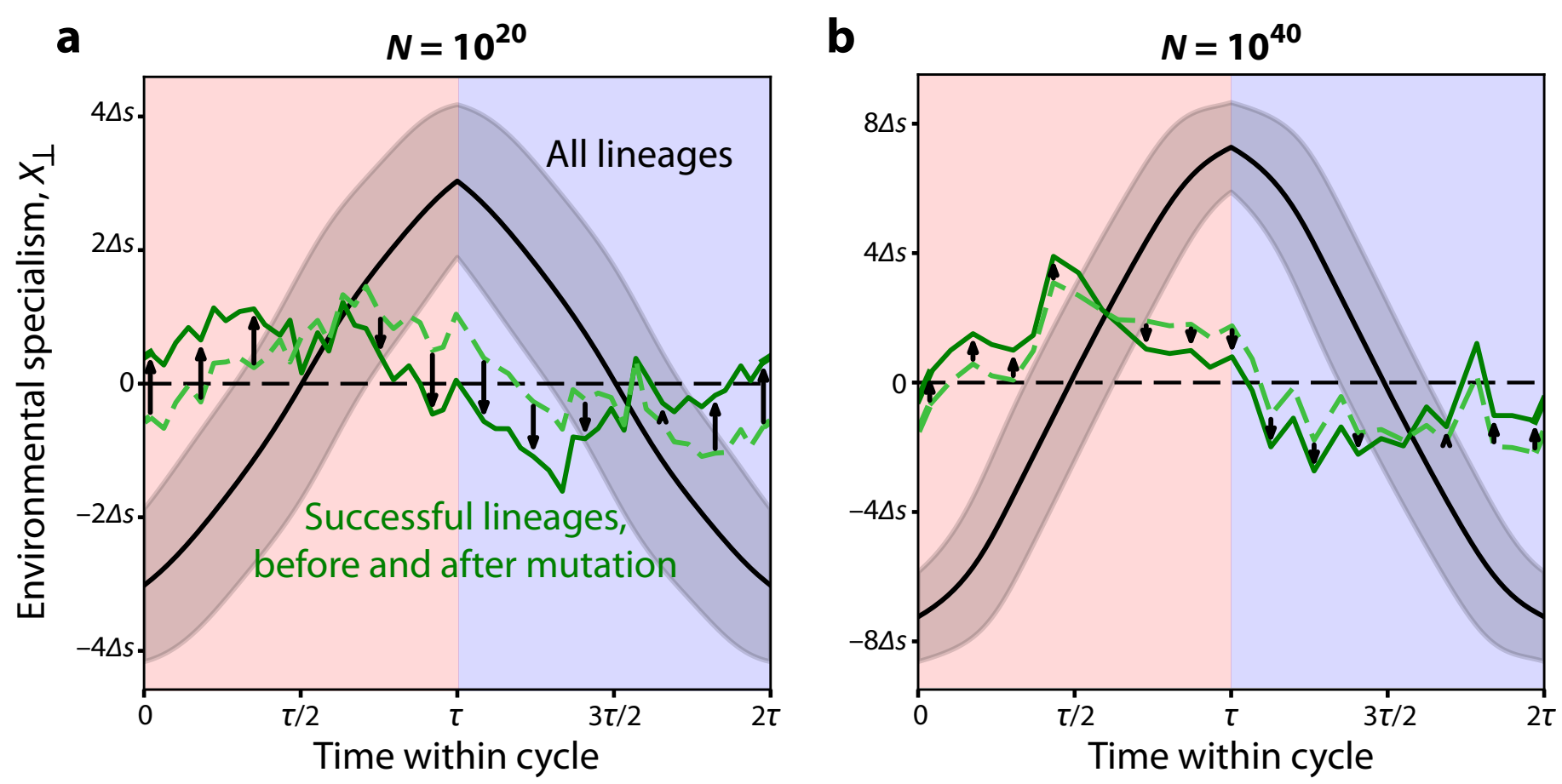

Figure S4: Optimal mutational path in asymptotically large populations. (a) The empirical specialism $X_\perp$ of lineages over a cycle, with the same simulation results as in Fig. 4B. Black points show the population mean, with shading $\pm$ one standard deviation. Green curves show the specialism of successful mutants (i.e., those fated for fixation) immediately before (dashed) and after (solid) a new mutation, with the arrows indicating the direction of mutation. Mutations are binned by establishment time. Simulations were run with $N = 10^{20}$, $U_g = U_s = 3 \cdot 10^{-9}$, $s = 0.02$, $\Delta s = 0.05$, and $\tau = 200$. **(b)** Similar plot as (a), but in a larger population with $N = 10^{40}$, $U_g = U_s = 5 \cdot 10^{-9}$, and $s = 0.03$. Note the y-axis spans a wider range of specialism.

bioRxiv preprint doi: https://doi.org/10.64898/2026.09.01.748641; this version posted September 4, 2026. The copyright holder for this preprint (which was not certified by peer review) is the author/funder, who has granted bioRxiv a license to display the preprint in perpetuity. It is made available under a CC-BY 4.0 International license.

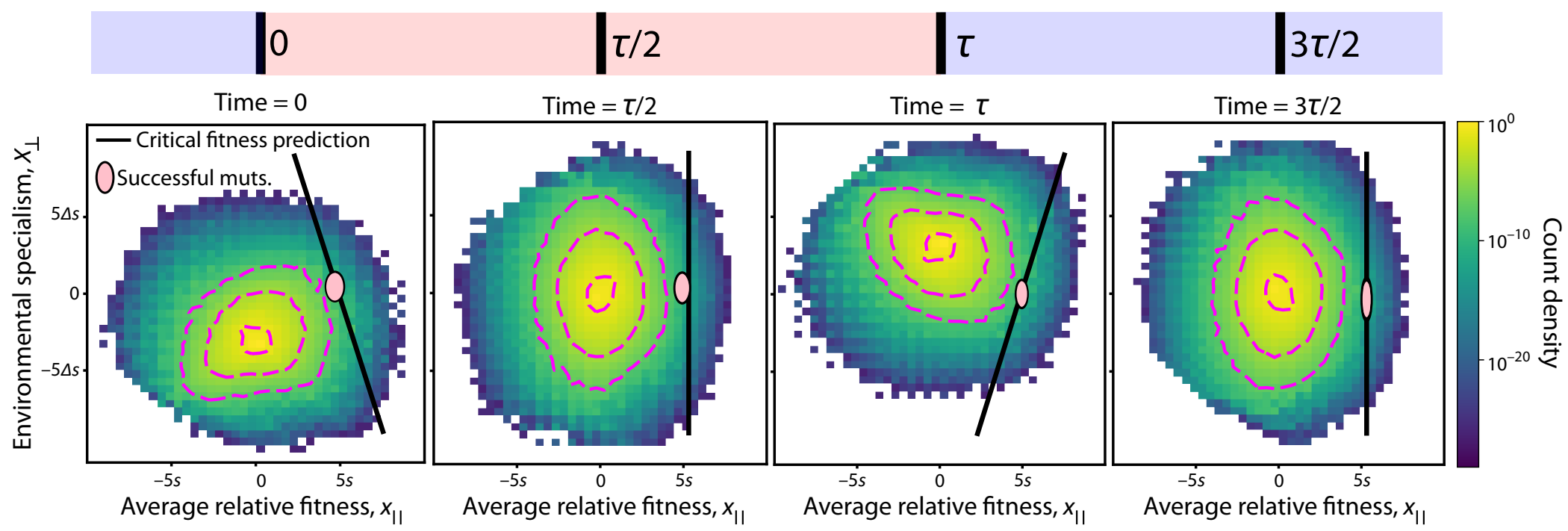

Figure S5: Two-dimensional fitness distribution in a population with frequent specialist mutations. Two-dimensional fitness distribution of the population at various points in the environmental cycle, parametrized by average fitness $X_{\|}$ and environmental preference $X_\perp$. Dashed magenta curves show empirical contour lines for densities 10^{-1}, 10^{-4} and 10^{-7}. Solid black line is the theory curve where $x_{\|}+s+\zeta^*(x_\perp+\delta s,t_0)=x_c$, with ζ defined in Eq. (S68); fixation is likely for mutatnts which arise on or to the right of this curve. Pink ellipse shows the average values of $x_{\|}$ and $X_\perp$ for lineages which have just acquired mutations fated for fixation, with the height and width of the ellipse equal to twice the standard deviation among these successful lineages in each dimension. Note that the pink ellipse is expected to roughly correspond with the maximum-density portion of the black line. An animated GIF of this distribution over the full time range can be found on Zenodo (56), where the position of the ellipse was inferred via linear interpolation. Simulation was run with $N=10^{20}$, $U_g=U_s=3\cdot10^{-9}$, $s=0.02$, $\Delta s=0.05$, and $\tau=200$.

bioRxiv preprint doi: https://doi.org/10.64898/2026.09.01.748641; this version posted September 4, 2026. The copyright holder for this preprint (which was not certified by peer review) is the author/funder, who has granted bioRxiv a license to display the preprint in perpetuity. It is made available under a CC-BY 4.0 International license.

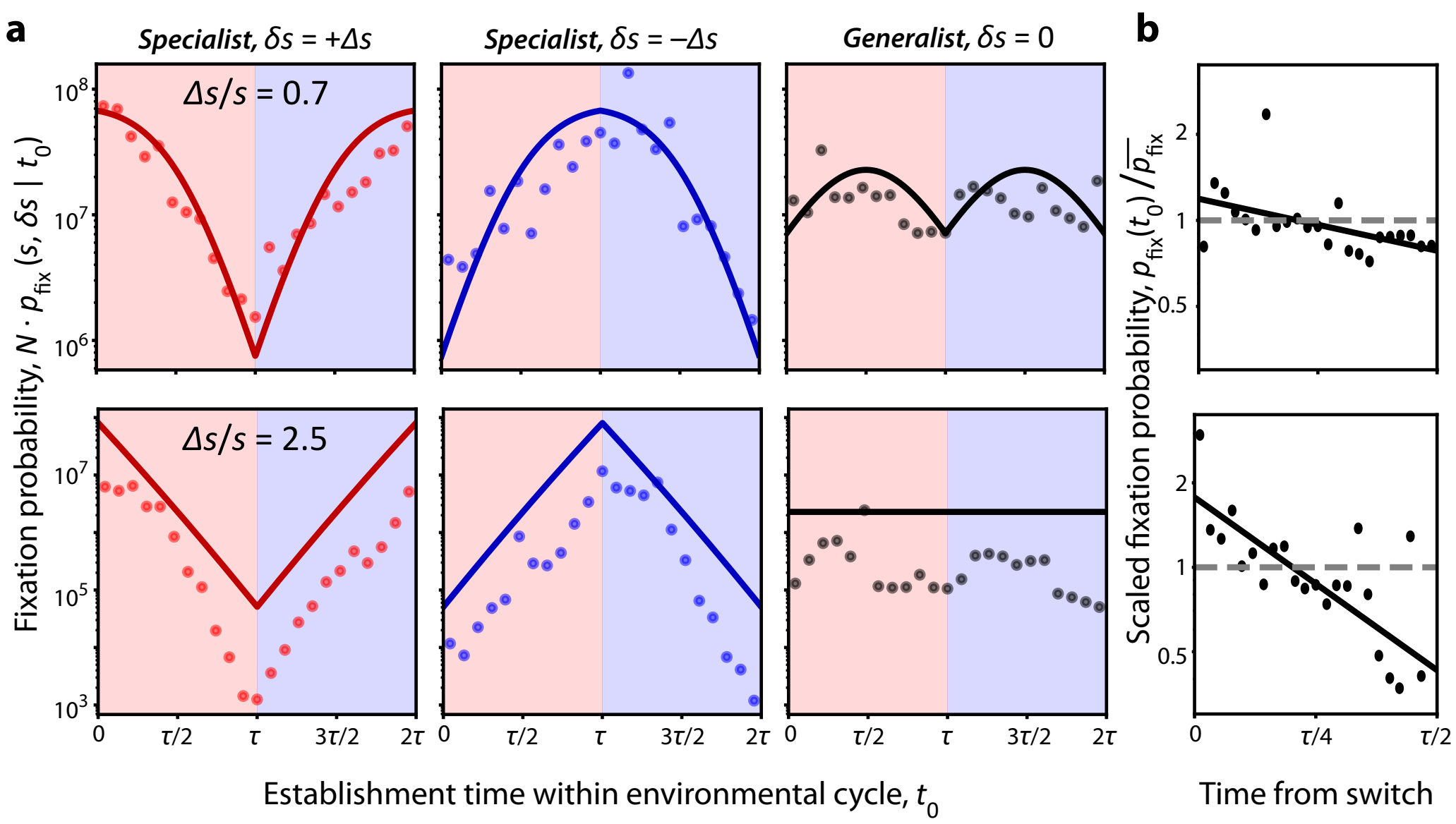

Figure S6: Time-dependent fixation probabilities when specialist mutations are frequent. **(a)** Fixation probabilities of each type of mutation as a function of their establishment time in a cycle, in a population where specialist mutations are common. Solid lines show theory prediction from Eq. (S102) (top row) or Eq. (S134) (bottom row). **(b)** Fixation probability averaged across all types of mutations, as a function of the time to or from the nearest environmental switch in the cycle. Dashed line shows the time-averaged fixation probability, while solid line is a best-fit line in log space, demonstrating that successful mutations tend to occur near environmental switches when tradeoffs are large. Top row: parameters are the same as Fig. 4B, with $\Delta s > s$. Bottom row: $N = 10^{20}$, $U_s = U_g = 10^{-10}$, $s = 0.022$, $\Delta s = 0.015$, and $\tau = 300$. Note that $s > \Delta s$, meaning tradeoffs are weaker than the generalist benefit of mutations.

bioRxiv preprint doi: https://doi.org/10.64898/2026.09.01.748641; this version posted September 4, 2026. The copyright holder for this preprint (which was not certified by peer review) is the author/funder, who has granted bioRxiv a license to display the preprint in perpetuity. It is made available under a CC-BY 4.0 International license.

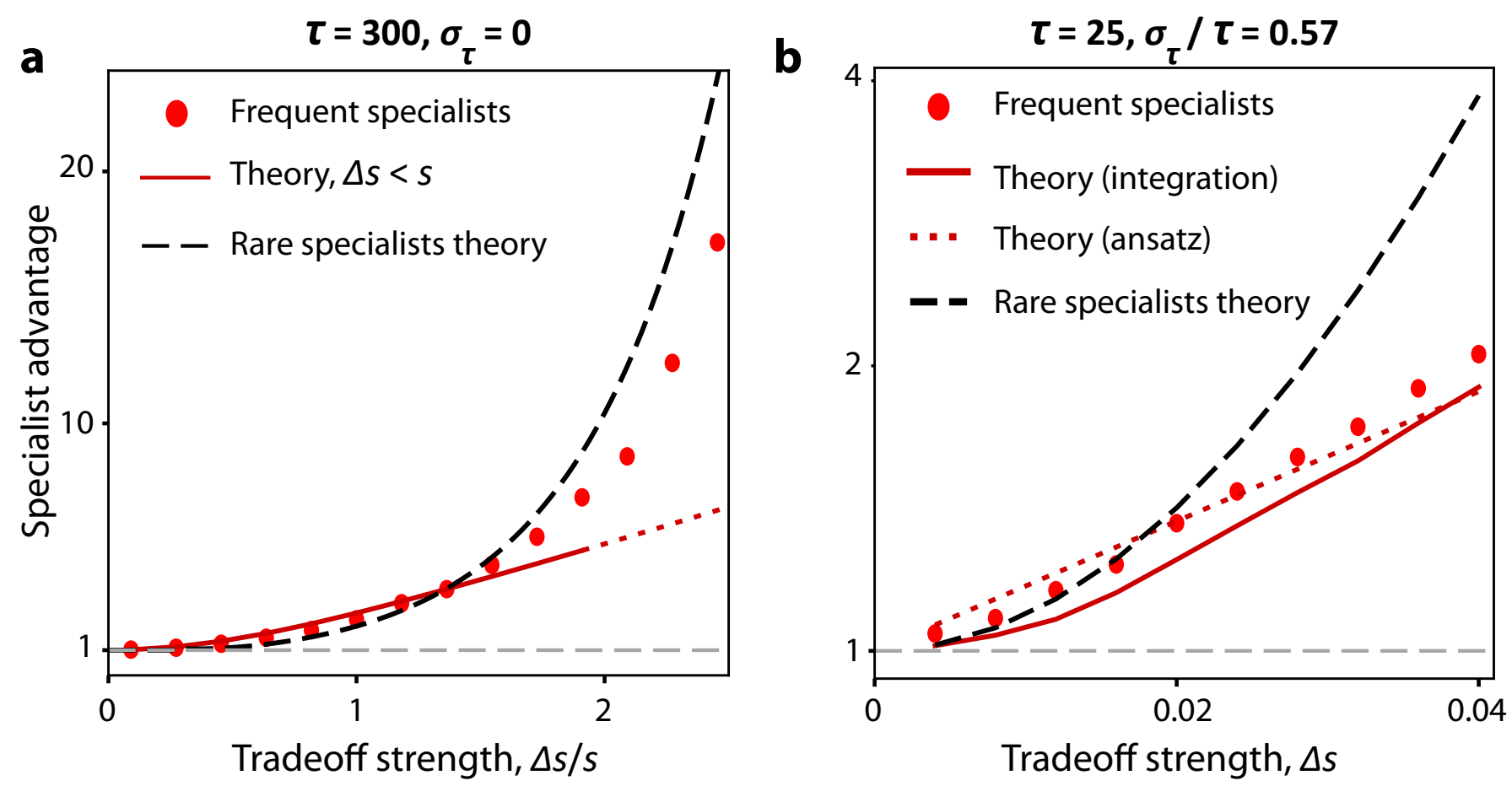

Figure S7: The specialist advantage depends on the mutation rate of specialist mutations. **(a)** The specialist advantage $p_{\text{fix}}(s, \Delta s)/p_{\text{fix}}(s, 0)$ in a periodic environment where specialist mutations are common. Points show simulation results, while red curve shows prediction from averaging Eq. (S102) over time, valid when $\Delta s < s$. Black dashed line shows rare-specialists prediction from Eq. (S31). Note that the specialist advantage increases to become exponential when $s > \Delta s$, similar to the rare-specialists regime. Parameters: $N = 10^{20}$, $s = 0.022$, $U_g = U_s = 10^{-10}$ (or $U_g = 2 \cdot 10^{-10}$ and $U_s \to 0$ for the rare-specialists curve). **(b)** The same plot for a stochastically fluctuating environment. Red curve shows prediction from numerically integrating Eq. (S156) for fit σ_{eff}; dotted red curve shows ansatz from Eq. (S165). Black curve shows rare-specialists prediction from Eq. (S46). Parameters: $N = 10^{20}$, $s = 0.02$, $U_g = U_s = 3 \cdot 10^{-9}$ (or $U_g = 6 \cdot 10^{-9}$ and $U_s \to 0$ for the rare-specialists curve).

bioRxiv preprint doi: https://doi.org/10.64898/2026.09.01.748641; this version posted September 4, 2026. The copyright holder for this preprint (which was not certified by peer review) is the author/funder, who has granted bioRxiv a license to display the preprint in perpetuity. It is made available under a CC-BY 4.0 International license.

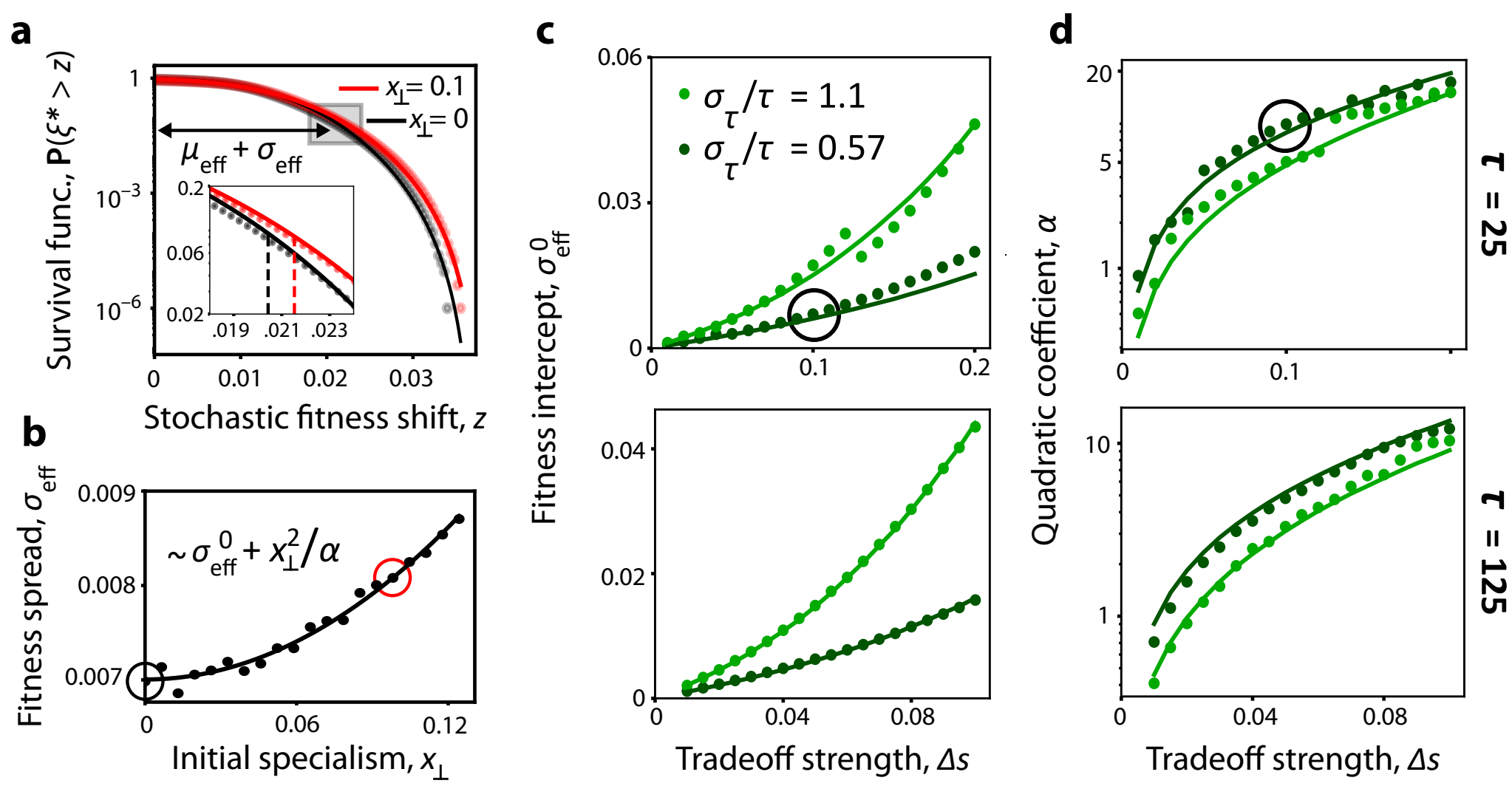

Figure S8: Characterizing the effect of seasonal drift on fixation in specializing populations. **(a)** Positive tail of the empirical survival function of the stochastic fitness shift ξ^*, defined in Eq. (S143), for two values of initial specialism $x_\perp$. Curves show the best fit of σ_{eff} to Eq. (S149). Inset shows a zoomed-in version of the shaded region with dashed lines indicating $\mu_{\text{eff}} + \sigma_{\text{eff}}$ for each distribution. **(b)** Dependence of fit σ_{eff} on $x_\perp$ was parametrized using a quadratic fit for $x_\perp$ between 0 and $2\Delta s$, yielding intercept σ^0_{eff} and quadratic scale α. Circles correspond to red and black curves in (a). **(c)** Dependence of the intercept σ^0_{eff} on Δs and σ_τ for small τ (top) and large τ (bottom). Curves show the ansatz in Eq. (S153) with fit $C_0 = 0.10$ (top) or 0.086 (bottom). Circled point corresponds to the parameters in (a). **(d)** Dependence of the quadratic scale α on Δs and σ_τ for small τ (top) and large τ (bottom). Points show fits as in (b), while curves the ansatz in Eq. (S154) with fit $C_\alpha = 1.2$ or 3.5 (bottom). Parameters in all panels are the same as Fig. 4C, with $v_\parallel$ and $v_\perp$ measured from simulations and x_c calculated as a function of $v_\parallel$.

bioRxiv preprint doi: https://doi.org/10.64898/2026.09.01.748641; this version posted September 4, 2026. The copyright holder for this preprint (which was not certified by peer review) is the author/funder, who has granted bioRxiv a license to display the preprint in perpetuity. It is made available under a CC-BY 4.0 International license.

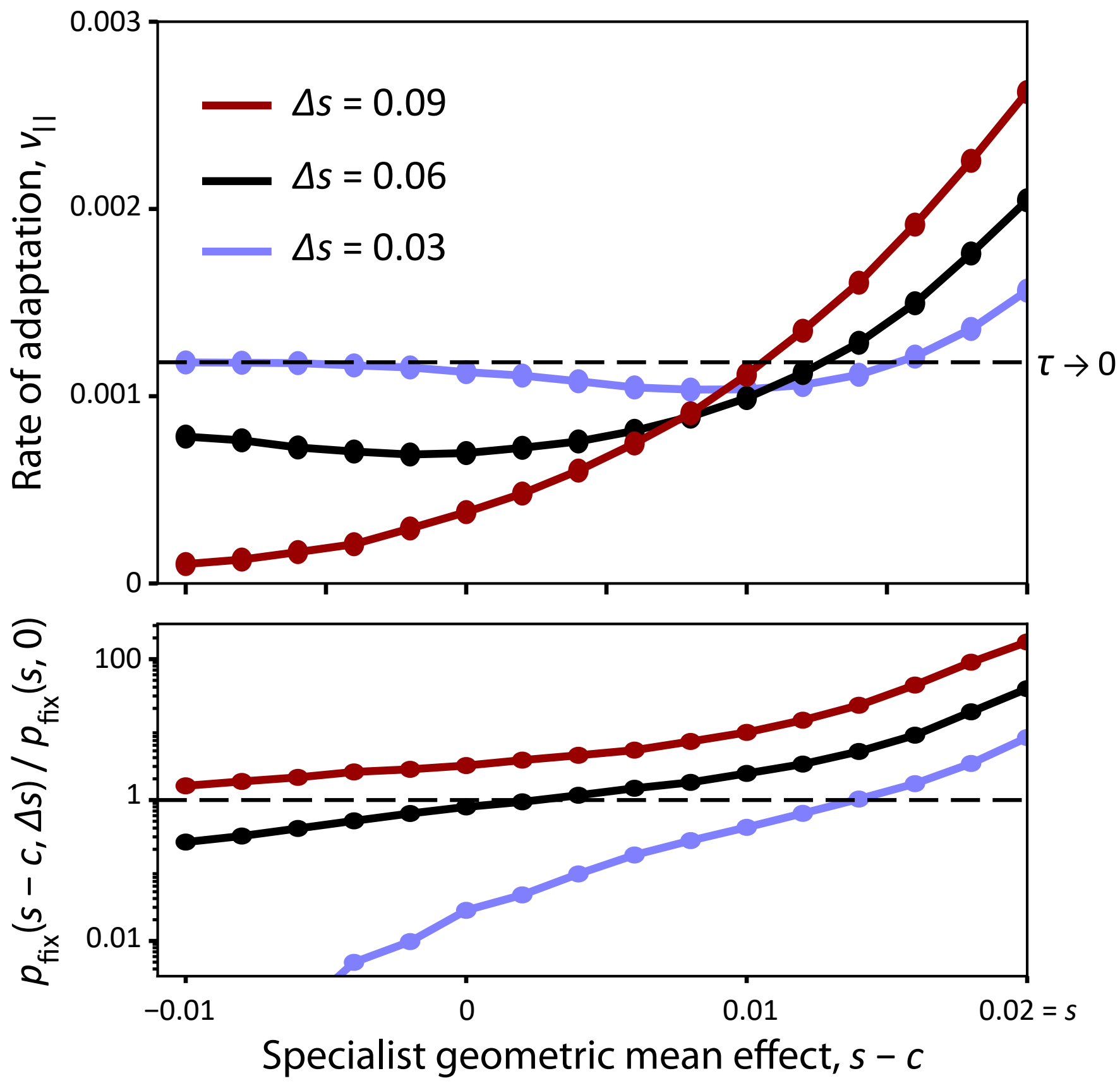

Figure S9: The rate of adaptation depends on the joint distribution of fitness effects. Top: Long-term rate of adaptation $v_{\|}$ in a stochastically varying environment measured in simulations where generalist mutations have fitness effect $(s, \Delta s)$ and specialist mutations have fitness effect $(s - c, \Delta s)$ for different fitness costs c and tradeoff strengths Δs. Dashed line shows the no-specialists rate of adaptation ($\tau \to 0$ or $U_g = 0$). Bottom: The specialist advantage for the same parameters as the top panel. The dashed line shows where fixation probabilities of specialist and generalist mutations are equal. Simulation parameters were $N = 10^{20}$, $U_g = U_s = 3 \cdot 10^{-9}$, $s = 0.02$, $\tau = 125$, and $\sigma_\tau/\tau = 1.1$.

bioRxiv preprint doi: https://doi.org/10.64898/2026.09.01.748641; this version posted September 4, 2026. The copyright holder for this preprint (which was not certified by peer review) is the author/funder, who has granted bioRxiv a license to display the preprint in perpetuity. It is made available under a CC-BY 4.0 International license.

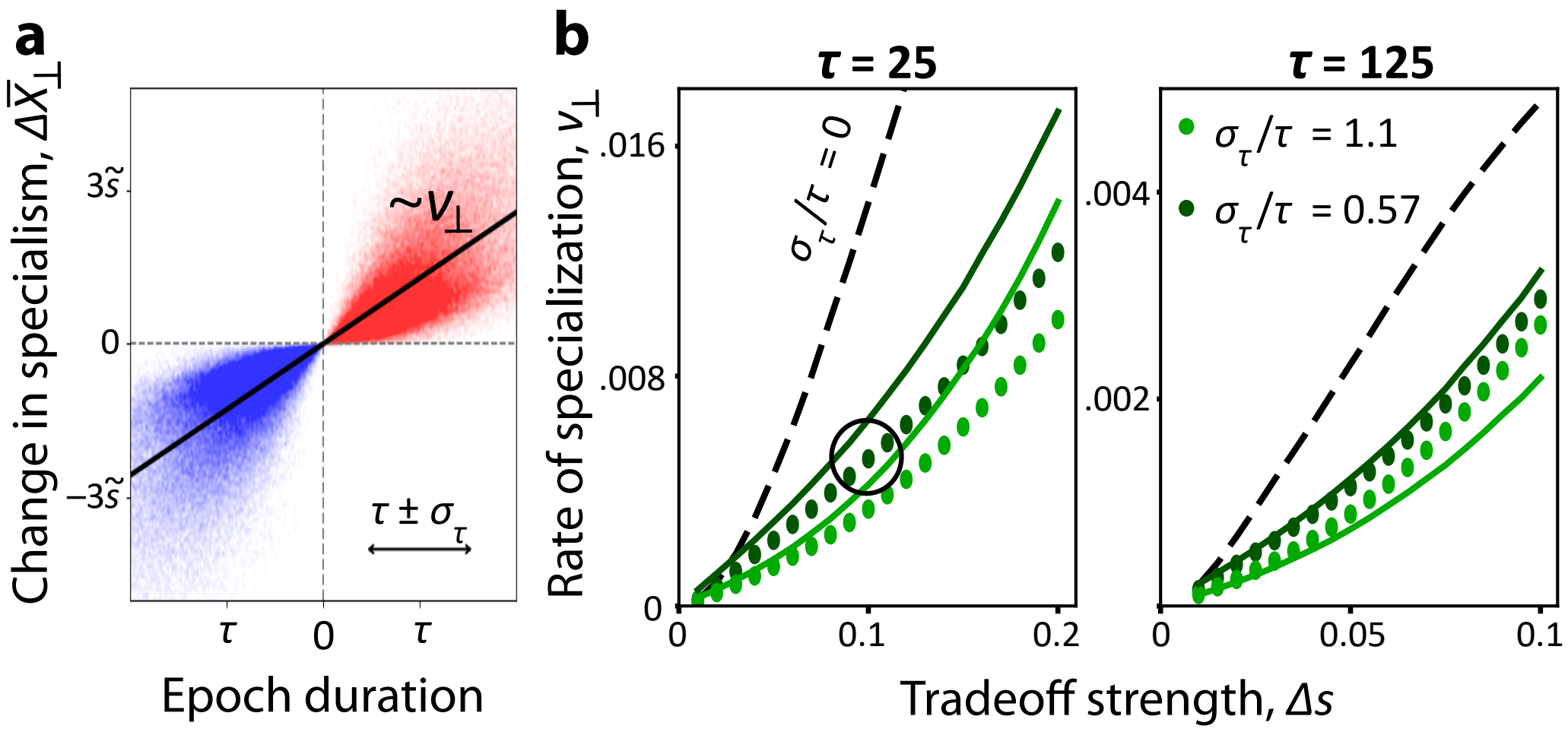

Figure S10: The rate of specialization declines in stochastic environments. (a) Scatterplot of the change in population mean specialism $\overline{X}_\perp$ over a single environmental epoch, as a function of the stochastic epoch length (with different environmental states reflected across zero). The slope of the best-fit line to this plot is defined as the rate of specialization $v_\perp$. **(b)** Measured rate of specialization $v_\perp$ for stochastic environments (points) and periodic environments (dashed line), compared to theory prediction in Eq. (S178), left, or Eq. (S179), right. Parameters in all panels were the same as Fig. 4C, with (a) corresponding to the circled point in (b).

bioRxiv preprint doi: https://doi.org/10.64898/2026.09.01.748641; this version posted September 4, 2026. The copyright holder for this preprint (which was not certified by peer review) is the author/funder, who has granted bioRxiv a license to display the preprint in perpetuity. It is made available under a CC-BY 4.0 International license.

1 Model Overview

1.1 Clonal Interference Regime

This work follows a body of literature modeling the evolution of populations in the "clonal interference" regime (38–41), where each beneficial mutation must compete with many others in order to reach fixation. Thus, much of our model setup follows previous work, whose central assumptions we restate here for completeness.

We consider a single-species population of constant size $N \gg 1$, whose members evolve over time by acquiring beneficial mutations. For simplicity, we model these mutations with a coarse-grained point distribution of fitness effects in the infinite-sites limit: beneficial mutations arise at a constant rate U (regardless of background), and each mutation has the same incremental log fitness effect s. Under these assumptions, any individuals with the same number of beneficial mutations will have the same fitness, making it convenient to define individuals by their "fitness class." Over time, new higher-fitness classes will be founded as old ones die out, and the absolute fitness of the population will increase.

Generally speaking, the empirically relevant clonal interference regime will apply for a broad range of parameters when $Ns \gg NU \gg 1$, such that many beneficial mutations arise each generation. More precisely, the parameter range we consider satisfies

$$\log^2(s/U) \gg 2\log(Ns) \gg \log(s/U). \tag{S1}$$

In physical terms, the left inequality requires that the fitness benefits of individual mutations are strong enough to be felt in the time it takes for a new fitness class to be founded. The right inequality states that mutations are frequent enough that at any given time, individuals with a range of fitnesses coexist, rather than a "pseudo-sweep" regime where only two fitness classes are present at once.

When these conditions are satisfied, the steady-state fitness distribution of the population can be described by a "traveling wave" with well-studied properties. This wave advances at rate v, the rate of adaptation of the population. While most individuals in the population have fitness close to the mean, the high-fitness "nose" of the wave has a fitness advantage x_c relative to the bulk. It is often convenient to work with these derived parameters, whose dependence on N and U has been calculated in previous work:

$$v \approx s^2 \cdot \frac{2\log(Ns)}{\log^2(s/U)}, \tag{S2}$$

$$x_c \approx \frac{v}{s}\log\left(\frac{s}{U}\right) + \frac{s}{2} \approx s \cdot \frac{2\log(Ns)}{\log(s/U)}. \tag{S3}$$

Expressed in these parameters, the clonal interference conditions are $s^2 \gg v$ and $x_c \gg s$. The distribution of relative fitness of the population can be approximated by

$$f(x) \approx \frac{e^{-x^2/2v}}{\sqrt{2\pi v}} \cdot \theta\left(x_c - |x|\right), \tag{S4}$$

where the Heaviside function $\theta(x)$ sets a fitness cutoff.

bioRxiv preprint doi: https://doi.org/10.64898/2026.09.01.748641; this version posted September 4, 2026. The copyright holder for this preprint (which was not certified by peer review) is the author/funder, who has granted bioRxiv a license to display the preprint in perpetuity. It is made available under a CC-BY 4.0 International license.

1.2 Mutations in a Fluctuating Environment

We would now like to extend the framework in the previous section to account for mutations whose effects depend on a time-varying environment. We can account for this in the simplest way possible by considering only two environments, E_1 and E_2. Suppose that the environmental state is given by a time-dependent indicator function $I(t) = \pm 1$, which applies to all members of the population. To begin with, we consider an environment where each environmental state $E_{1,2}$ lasts exactly $\tau_{1,2}$ generations:

$$I(t) \equiv \begin{cases} 1 & \text{if } 0 \leq t < \tau_1, \\ -1 & \text{if } \tau_1 \leq t < \tau_1 + \tau_2, \end{cases} \tag{S5}$$

and is defined by the periodic relationship $I(t + \tau_1 + \tau_2) = I(t)$ afterward. Through most of the main text, we focus on the symmetric case $\tau \equiv \tau_1 = \tau_2$ (although see SI 2.1.5 for the more general case). In this symmetric case, it is also useful to define the function

$$R(t) \equiv \min(t, 2\tau - t) \tag{S6}$$

for $0 \leq t < 2\tau$, and periodic with period 2τ otherwise. This triangle wave has the useful interpretation that $R(t_1) - R(t_0)$ is equal to the difference in the number of generations spent in each environmental state between t_0 and t_1. Later, we will extend this framework to consider the impact of seasonal drift, where the duration of each environmental epoch is a random variable.

The (log) fitness effect of a general mutation must now be expressed as a two-dimensional quantity, $(s_1, \ s_2)$. It is convenient to reparametrize these fitness effects into geometric mean fitness $\bar{s}$ and "tradeoff strength" Δs, defined as

$$\bar{s} \equiv \frac{\tau_1 s_1 + \tau_2 s_2}{\tau_1 + \tau_2}, \tag{S7a}$$

$$\Delta s \equiv \frac{s_1 - s_2}{2}. \tag{S7b}$$

In the symmetric case $\tau_1 = \tau_2$, these new variables form an orthogonal basis and the original fitness effects can be simply written as $(s_1, s_2) = (\bar{s} + \Delta s, \bar{s} - \Delta s)$.

Aggregating over the fitness effects of all possible mutations will yield a two-dimensional joint distribution of fitness effects (JDFE), which we choose to express in the $(\bar{s}, \Delta s)$ basis. In this work, we will focus on a particularly simple JDFE where mutations are grouped into three classes, which each have constant fitness effects. "Generalist mutations" have fitness effect $(s, 0)$, providing the same constant fitness boost s in both environments (notated as s_g in the main text). By contrast, there are two symmetric classes of "specialist mutations" with fitness effects $(\bar{s}, +\Delta s)$ and $(\bar{s}, -\Delta s)$. The tradeoff component Δs of these mutations causes them to favor one environment over the other. We will suppose that generalist mutations arise at rate U_g, and each type of specialist arises at rate $U_s/2$, regardless of genetic background.

bioRxiv preprint doi: https://doi.org/10.64898/2026.09.01.748641; this version posted September 4, 2026. The copyright holder for this preprint (which was not certified by peer review) is the author/funder, who has granted bioRxiv a license to display the preprint in perpetuity. It is made available under a CC-BY 4.0 International license.

The behavior of the population will be highly sensitive to the environmental fluctuation timescale. In particular, past research suggests the existence of simple regimes where mutations in fluctuating environments behave like mutants in static ones. The first case is $\tau_{1,2} \ll 1/\Delta s$, where the environment switches too quickly for the tradeoff to significantly change the frequency of a specialist mutation. In this case, selection will proceed along the direction of geometric mean fitness, so only the mean fitness effect $\bar{s}$ of the mutation matters. Conversely, when τ_1 and τ_2 are greater than the fixation time T_{fix}, then the mutant will either reach fixation or go extinct in a single environment, so its fitness in the other environment will be irrelevant. This fixation time is known to be $T_{\text{fix}} \approx x_c/v$ for a traveling wave in a constant environment. However, these limiting cases do not describe a wide range of intermediate fluctuation times,

$$1/\Delta s \ll \tau_{1,2} \ll x_c/v, \tag{S8}$$

where specialist mutations with environmental tradeoff Δs may behave very differently from generalist mutations with $\Delta s = 0$. The rest of our analysis focuses on this intermediate regime.

We will also specialize to the regime where the tradeoff strength is not so strong that it dominates the spread in generalist fitnesses in the population, requiring

$$\Delta s \ll x_c. \tag{S9}$$

Under this condition, specialist mutations which arise on high-fitness backgrounds can have a positive initial growth rate $\sim x_c \pm \Delta s$ regardless of the environmental state. Importantly, this does not set a restriction on the relative sizes of $\bar{s}$ and Δs, meaning that tradeoffs of individual mutations can still be strong or weak relative to their average effect.

A final important scale is the dimensionless parameter $v\tau_{1,2}/s$, which can either be much larger or smaller than unity given our other assumptions. In the fast-cycling regime defined by $v\tau_{1,2}/s \ll 1$, a newly established lineage will not produce a new beneficial mutation until many environmental cycles have passed. In the slow-cycling regime $v\tau_{1,2}/s \gg 1$, a lineage founded near the beginning of an environmental state is likely to have descendants with many new mutations by the time the environment switches. We will consider the effects of environmental fluctuations in both of these regimes and find that they are often surprisingly similar, although we will sometimes specialize to one regime for ease of calculation.

2 Fixation Probability of a Rare Specialist

To understand how specialist mutations behave when subject to clonal interference, we will first aim to calculate their fixation probability while rare. More precisely, we will assume that the specialist mutation rate U_s and the generalist mutation rate U_g satisfy $NU_s \ll 1 \ll NU_g$, so that no more than one specialist lineage is typically present in the population at any given time. This assumption enables us to consider the fixation probability of a single specialist lineage competing against a purely generalist background, which is

bioRxiv preprint doi: https://doi.org/10.64898/2026.09.01.748641; this version posted September 4, 2026. The copyright holder for this preprint (which was not certified by peer review) is the author/funder, who has granted bioRxiv a license to display the preprint in perpetuity. It is made available under a CC-BY 4.0 International license.

well-described by the traveling wave framework discussed above. For simplicity, we will also assume symmetry in temporal fluctuations ($\tau \equiv \tau_1 = \tau_2$), relaxing this assumption in SI 2.1.5.

Crucially, the specialist lineage is able to acquire additional generalist mutations at rate U_g (just like the rest of the population), which increase its fitness by s but have no effect on its level of specialism. In biological terms, rare specialists might arise in an evolving population where a small fraction of mutations can exploit a subtly changing environmental niche, which most of the population is insensitive to. More broadly, the rare-specialists regime will provide crucial intuition for the more generic $U_s \sim U_g$ case, making it useful to analyze first.

2.1 Fixation in a Deterministic Environment

We will first analyze the case of a perfectly periodic environment, which switches between states every τ generations according to Eq. (S5).

2.1.1 Successive Establishments and Rolling Fitness

To analyze the fixation of specialist lineages, we will employ a heuristic approach, tracking the time it takes a lineage to found successively more-fit offspring. This calculation is similar to that of Ref. (42), extended to the case of fluctuating environments. The central idea is that the fate of a lineage will be determined by whether its descendants are able to increase their fitness (by acquiring additional mutations) faster than the rest of the population.

Consider a specialist mutation which arises at time t_0 on an otherwise generalist genetic background. Its initial relative fitness will be $x_0 + I(t_0) \cdot \Delta s$, where x_0 is the geometric mean fitness, which depends on the mutation's original genetic background. If the specialist genotype establishes in the population (i.e., rises to high enough frequency to escape initial genetic drift), it will thereafter undergo approximately deterministic dynamics,

$$f_0(x_0, t_0, t) \approx \frac{1}{2N(x_0 + \Delta s I(t_0))} e^{x_0 t - vt^2/2 + \Delta s \cdot [R(t+t_0) - R(t_0)]}, \tag{S10}$$

where t tracks the number of generations since the genotype's establishment. We will focus on high-fitness genotypes with $x_0 \sim x_c$, since lower-fitness genotypes are unlikely to contribute to fixation. In this case, x_0 will be the dominant fitness scale, and non-exponential correction factors such as the $\Delta s I(t_0)$ in the denominator can be ignored.

Eventually, this genotype will begin establishing beneficial generalist mutations, founding a new fitness class with higher absolute fitness. Ref. (42) demonstrates that because the new fitness class grows more quickly than its parent genotype, it will be dominated by descendants of the *first* successful mutation, rather than future mutants that have had less time to grow at higher fitness. This result implies that the frequency trajectory of the new class can be approximated as that of a single genotype, of the same form as Eq. (S10) – but

bioRxiv preprint doi: https://doi.org/10.64898/2026.09.01.748641; this version posted September 4, 2026. The copyright holder for this preprint (which was not certified by peer review) is the author/funder, who has granted bioRxiv a license to display the preprint in perpetuity. It is made available under a CC-BY 4.0 International license.

with a new mean establishment fitness x_1. Accounting for environmental fluctuations also requires that we keep track of the new establishment time t_1, since the trajectory depends on when in the environmental cycle a lineage established. The same pattern will hold for the offspring of the new genotype, and so on, such that the frequency of the genotype with k additional beneficial mutations is

$$f_k(x_k, t_k, t) \approx \frac{1}{2Nx_k} e^{x_k t - vt^2/2 + \Delta s \cdot [R(t+t_k) - R(t_k)]} \tag{S11}$$

t generations after it establishes. As shown in Ref. (42), we can write recursive relations for x_k and t_k which depend on the random variable T_{k-1}, the number of generations between t_k and t_{k-1}:

$$x_k \approx x_{k-1} + s - vT_{k-1}, \tag{S12a}$$

$$t_k = t_{k-1} + T_{k-1}. \tag{S12b}$$

The last two terms on the RHS of Eq. (S12a) have a simple interpretation: it takes T_{k-1} generations for the focal lineage to produce a mutation with fitness effect s, during which time the rest of the population increases its average fitness by vT_{k-1}. A successful lineage will tend to increase its "rolling fitness" x over many successive establishments, until the lineage reaches high frequency in the population and achieves fixation. Conversely, typical lineages will be outcompeted by the rest of the population, until x falls to the point that the specialist lineage goes extinct. Thus, the fixation probability of the specialist mutation is equivalent to the probability that x tends to increase over the long term, marginalizing over the distributions of T_k. Most of the complexity in this process is therefore hidden in the random variables T_k, which we discuss in the upcoming sections.

2.1.2 Long-term Rolling Fitness with Environmental Tradeoffs

The rate at which a genotype described by Eq. (S11) establishes new beneficial generalist mutations is

$$\begin{aligned} n_{\text{est}}(x_k, t_k, t) &= 2NU_g(x_k + s - vt) \cdot f_k(x_k, t_k, t), \\ &\approx U_g \exp\left[x_k t - \frac{v}{2}t^2 + \Delta s \left(R(t+t_k) - R(t_k)\right)\right]. \end{aligned} \tag{S13}$$

To uncover the key effects of environmental fluctuations, it is sufficient to consider only the *typical* values of T_k. This typical waiting time (which we will also denote by T_k for convenience) is defined as the time when the expected number of mutations founded becomes order unity,

$$\begin{aligned} 1 &\approx \int_0^{T_k} n_{\text{est}}(x_k, t_k, t)dt, \\ &\approx \frac{U_g}{x_k} \exp\left[x_k T_k - \frac{v}{2}T_k^2 + \Delta s R(T_k + t_k) - \Delta s R(t_k)\right], \\ &\approx \frac{s}{x_k} \exp\left[x_k T_k - \frac{v}{2}T_k^2 + \Delta s R(T_k + t_k) - \Delta s R(t_k) - \frac{x_c s}{v} + \frac{s^2}{2v}\right] \end{aligned} \tag{S14}$$

bioRxiv preprint doi: https://doi.org/10.64898/2026.09.01.748641; this version posted September 4, 2026. The copyright holder for this preprint (which was not certified by peer review) is the author/funder, who has granted bioRxiv a license to display the preprint in perpetuity. It is made available under a CC-BY 4.0 International license.

after plugging in the relationship in Eq. (S3). Neglecting the $\sim s/x_c$ logarithmic prefactor, the solution to this equation satisfies

$$T_k \approx \frac{1}{v}\left[x_k - \sqrt{x_k^2 - 2sx_c + s^2 + 2v\Delta s \cdot \Delta R_k - 2v\log(x_k/s)}\right] \tag{S15}$$

$$\approx \frac{s}{v}\left(1 - \frac{x_k - x_c}{x_c}\right) - \frac{\Delta s}{x_c}\left(1 + \frac{s}{x_c}\right)\Delta R_k + \mathcal{O}\left(\frac{\log(x_c/s)}{x_c}\right). \tag{S16}$$

for $|x_k - x_c| \ll x_c$, where $\Delta R_k \equiv R(t_{k+1}) - R(t_k)$. Plugging this into Eq. (S12a) yields a solution to the recursive rolling fitness $\overline{x}$ over successive foundings:

$$x_n \approx x_c + e^{ns/x_c}\left[x_0 - x_c + \frac{v\Delta s}{x_c}\sum_{i=0}^{n-1} e^{-(i+1)s/x_c}\Delta R_i\right]. \tag{S17}$$

Because the ΔR_i depend on t_i, this solution is still not fully explicit. In the main text, we make the additional assumption that $\tau \gg s/v$, so that t_k and $t_k + T_k$ will usually be in the same environmental cycle. In this case, we can simplify $\Delta R_i \approx (s/v) \cdot I(t_i)$. However, a more general approach is to expand the sum

$$\sum_{i=0}^{n-1} e^{-(i+1)s/x_c}\Delta R_i \approx \frac{s}{x_c}\sum_{i=1}^{n-1} e^{-is/x_c}R(t_i) - e^{-s/x_c}R(t_0) + e^{-ns/x_c}R(t_n). \tag{S18}$$

The fate of the focal lineage will be determined at least a coalescence time after it arises, $n \gtrsim x_c/s$. In this case, we can neglect the third term and approximate the slowly decaying sum in the first term as converging to its average, $\overline{R} = \tau/2$. This yields

$$x_n \approx x_c + e^{ns/x_c}\left[x_0 - x_c + \frac{v\Delta s}{x_c}\left(\frac{\tau}{2} - R(t_0)\right)\right]. \tag{S19}$$

Fixation will be likely if the contents of the brackets in Eq. (S19) are positive, so that x_n increases with time. Thus, the tradeoff component of the specialist mutation acts like an effective shift in the initial geometric mean fitness x_0, which depends on the time the lineage arises:

$$s_{\text{eff}}(t_0) = \frac{v\Delta s}{x_c}\left(\frac{\tau}{2} - R(t_0)\right). \tag{S20}$$

A specialist mutation with mean fitness effect $\bar{s}$ and tradeoff Δs will therefore have a similar fixation probability to a generalist mutation with mean fitness effect $\bar{s} + s_{\text{eff}}$ and no tradeoff. Lineages which establish at the optimal time ($R(t_0) = 0$, at the start of a beneficial epoch) will be capable of fixing even if they start at a slightly lower fitness. Conversely, specialist lineages which establish at the start of a deleterious period ($R(t_0) = \tau$) must have a higher average fitness than a corresponding generalist lineage to avoid going extinct.

The source of this time-dependence is evident in Eq. (S17). Small differences in rolling fitness compound over successive foundings (even for a generalist lineage with $\Delta s = 0$), because a lineage with higher starting fitness founds further beneficial mutations faster, giving less time for the rest of the population to adapt. The influence of the environment

bioRxiv preprint doi: https://doi.org/10.64898/2026.09.01.748641; this version posted September 4, 2026. The copyright holder for this preprint (which was not certified by peer review) is the author/funder, who has granted bioRxiv a license to display the preprint in perpetuity. It is made available under a CC-BY 4.0 International license.

enters as a weighted sum in Eq. (S17) which places more weight on environmental states soon after the lineage arises, because they have more time to compound in the future. Specifically, a lineage that establishes at the start of a beneficial period will found its next mutations more quickly, giving it a fitness head start which increases its rate of founding future beneficial mutations, even in a deleterious period. Thus, the fate of a specialist mutation will depend on the time it arises even many environmental cycles later, rather than the environments averaging out.

2.1.3 *Jackpot Mutations*

Our next goal is to map the fitness shift in Eq. (S20) to the fixation probability $p_{\text{fix}}(\bar{s}, \Delta s \mid x)$ of a specialist mutation which arises on a genetic background with fitness x. Since we have already calculated the effective fitness shift induced by environmental tradeoffs, the remainder of the calculation closely follows previous work (42), and is consistent with past results predicting how the fixation probability depends on the fitness effect of a mutation (39). We reproduce the main features of the calculation here for clarity.

In the previous section, we employed a deterministic approximation to the mutation waiting time T_k. This corresponds to a "step function" approximation to the fixation probability, where fixation occurs if the brackets in Eq. (S19) are positive: i.e. if $x_0 > \tilde{x}_c(t_0)$ where $\tilde{x}_c$ is a critical "break-even" fitness scale defined as

$$\tilde{x}_c(t_0) \equiv x_c - s_{\text{eff}}(t_0). \tag{S21}$$

While the fixation probability indeed experiences a critical shift around this value of x_0, accounting for stochasticity in T_k is important to predict it more accurately. This is because successful lineages can acquire atypically early "jackpot" mutations that increase their rolling fitness more than expected, allowing them to fix even if their starting fitness is slightly below $\tilde{x}_c$.

The probability density of establishing an early jackpot mutation in only t generations is given by Eq. (S13). The corresponding establishment fitness is $x_{k+1} = x_k + s - vt$. Changing variables from t to x_{k+1}, the probability of jackpotting from x_k to x_{k+1} is

$$\begin{aligned} p_{\text{jackpot}}(x_{k+1}) &\approx \frac{U_g}{v} \exp\left[\frac{x_k^2 - x_{k+1}^2}{2v} + \frac{s(2x_{k+1} - s)}{2v} + \Delta s \left[R\left(t(x_{k+1}) + t_k\right) - R(t_k)\right]\right], \\ &\approx \frac{s}{v} \exp\left[\frac{x_k^2 - x_{k+1}^2}{2v} + \frac{s(x_{k+1} - x_c)}{v} + \Delta s \left[R\left(t(x_{k+1}) + t_k\right) - R(t_k)\right]\right]. \end{aligned} \tag{S22}$$

We can remove the remaining dependence on t by changing variables to $\chi_k \equiv x_k - \tilde{x}_c(t_k)$, yielding

$$p_{\text{jackpot}}(\chi_1 | \chi_0) \approx \frac{s}{v} \exp\left[\frac{x_c}{v}(\chi_0 - \chi_1) + \frac{\chi_0^2 - \chi_1^2 + 2s\chi_1}{2v}\right], \tag{S23}$$

bioRxiv preprint doi: https://doi.org/10.64898/2026.09.01.748641; this version posted September 4, 2026. The copyright holder for this preprint (which was not certified by peer review) is the author/funder, who has granted bioRxiv a license to display the preprint in perpetuity. It is made available under a CC-BY 4.0 International license.

retaining only the dominant terms in the exponent. This jackpotting probability is identical to the constant-environment jackpotting probability in Ref. (42), but in terms of χ rather than x. Thus, the calculation of the fixation probability proceeds identically:

$$p(\chi) \approx p(\chi + s\chi/x_c)\left(1 - \int_{\chi+v/x_c}^{\infty} p_{\text{jackpot}}(\chi'|\chi)d\chi'\right) + \int_{\chi+v/x_c}^{0} p_{\text{jackpot}}(\chi'|\chi)p(\chi')d\chi' + \int_{0}^{\infty} p_{\text{jackpot}}(\chi'|\chi)d\chi', \tag{S24}$$

where $p(\chi)$ is the fixation probability of a lineage which establishes at shifted fitness χ. The first term represents the deterministic decline of a non-jackpotting lineage with $\chi < 0$, the second term is the probability of jackpotting below the break-even fitness, and the third term is the probability of jackpotting above the break-even fitness (followed by deterministic fixation). The solution to this equation is

$$p(\chi) \approx \exp\left(\frac{\chi(2x_c + \chi)}{2v}\right) \tag{S25}$$

for $\chi < 0$, or

$$p(x, t_0) \approx \exp\left[\frac{x^2 - x_c^2}{2v} + \Delta s\left(\frac{\tau}{2} - R(t_0)\right) + \mathcal{O}\left(\frac{xs\tau\Delta s}{x_c^2}\right)\right] \tag{S26}$$

in untransformed fitness units, where t_0 is the establishment time of the lineage within a cycle. Because this is the fixation probability of a lineage after establishment, the full fixation probability of a new mutation also accounts for the probability of escaping genetic drift:

$$p_{\text{fix}}(\bar{s}, \Delta s \mid x, t_0) \approx 2x \cdot \exp\left[\frac{(x + \bar{s})^2 - x_c^2}{2v} + \Delta s\left(\frac{\tau}{2} - R(t_0)\right)\right], \tag{S27}$$

which applies when $x \lesssim \tilde{x}_c(t_0)$.

2.1.4 *Overall Fixation Probability*

The overall fixation probability of a specialist mutation with fitness effect $\bar{s} \pm \Delta s$ can be calculated by integrating over the starting fitness and establishment time of the mutation:

$$p_{\text{fix}}(\bar{s}, \Delta s) = \int_{-\infty}^{x_c} dx \int_{0}^{2\tau} \frac{dt_0}{2\tau} \cdot p_{\text{fix}}(\bar{s}, \Delta s \mid x, t_0) \cdot f(x), \tag{S28}$$

with $f(x)$ defined in Eq. (S4) and p_{fix} in Eq. (S27). The dominant contribution to the integral over background fitness x will be $x \gtrsim \tilde{x}_c - \bar{s} - \mathcal{O}(v/\bar{s})$, just below the break-even fitness:

$$p_{\text{fix}}(\bar{s}, \Delta s \mid t_0) \approx \frac{x_c}{\bar{s}}\sqrt{\frac{2v}{\pi}} \exp\left[-\frac{(\tilde{x}_c - \bar{s})^2}{2v} - \Delta s \cdot R(t_0)\right]. \tag{S29}$$

bioRxiv preprint doi: https://doi.org/10.64898/2026.09.01.748641; this version posted September 4, 2026. The copyright holder for this preprint (which was not certified by peer review) is the author/funder, who has granted bioRxiv a license to display the preprint in perpetuity. It is made available under a CC-BY 4.0 International license.

Because Eq. (S27) is only valid for $x < \tilde{x}_c$, we must verify this contribution is greater than the contribution of mutations that arise above $\tilde{x}_c$ and fix deterministically after escaping genetic drift, where $p_{\text{fix}} \approx 2x$. The dominant contribution in this drift-dominated region will be $x \lesssim \tilde{x}_c - \bar{s} + \mathcal{O}(v/x_c)$, just above the break-even fitness. Ignoring smaller correction terms, we find

$$p_{\text{drift}}(t_0) \approx \sqrt{\frac{2v}{\pi}} \exp\left[-\frac{(\tilde{x}_c - \bar{s})^2}{2v} - \Delta s \cdot R(t_0)\right]. \tag{S30}$$

This is a factor of $x_c/\bar{s}$ smaller than Eq. (S29), so it can be safely ignored, in which case Eq. (S29) approximates the entire integral. Finally, we can integrate over time, which will be dominated by times within $1/\Delta s$ of $t_0 = 0$:

$$p_{\text{fix}}(\bar{s}, \Delta s) \approx \frac{1}{\tau \Delta s} \frac{x_c}{\bar{s}} \sqrt{\frac{2v}{\pi}} \exp\left[-\frac{(x_c - \bar{s} - v\tau\Delta s/2x_c)^2}{2v}\right]. \tag{S31}$$

This is larger than the fixation probability of a generalist with the same geometric mean fitness effect,

$$p_{\text{fix}}(\bar{s}, 0) \approx \frac{x_c}{\bar{s}} \sqrt{\frac{2v}{\pi}} \exp\left[-\frac{(x_c - \bar{s})^2}{2v}\right]. \tag{S32}$$

If the generalist has a different fitness effect s_g, the log-ratio between the fixation probability of a specialist and a generalist is

$$\log\left(\frac{p_{\text{fix}}(\bar{s}, \Delta s)}{p_{\text{fix}}(s_g, 0)}\right) \approx \frac{\tau \Delta s}{2} - \log(\tau \Delta s) + \frac{x_c}{v}(\bar{s} - s_g). \tag{S33}$$

The first term reflects the increase in fixation probability for a specialist which arises at the ideal time, while the second term represents the reduced probability of the mutant appearing in this critical period. The final term simply accounts for any difference in average fitness between the mutation types, and is zero for the symmetric case $\bar{s} = s_g$. Note that while the logarithmic term is subdominant for $\Delta s \cdot \tau \gg 1$, it prevents the deterministic specialist advantage from becoming notable until $\Delta s \cdot \tau \gtrsim 10$, broadening the transition from the weak-tradeoffs regime $\Delta s \cdot \tau \ll 1$.

2.1.5 Asymmetric Temporal Fluctuations

Here, we briefly discuss the impact of considering a periodic environment which does not spend an equal amount of time in each environmental state ($\tau_1 \neq \tau_2$). We will find that our main conclusions still hold, but the harmonic mean of the environmental durations becomes the relevant timescale.

When one environmental state lasts longer than the other, natural selection will more strongly favor mutations that increase fitness in that environment. However, this effect is already described by the geometric mean fitness defined in Eq. (S7a), which weighs each environmental state by its duration. The effect of environmental *tradeoffs* is given by the

bioRxiv preprint doi: https://doi.org/10.64898/2026.09.01.748641; this version posted September 4, 2026. The copyright holder for this preprint (which was not certified by peer review) is the author/funder, who has granted bioRxiv a license to display the preprint in perpetuity. It is made available under a CC-BY 4.0 International license.

influence of Δs for a fixed $\bar{s}$. We can calculate this by generalizing the sum in Eq. (S19) to asymmetric environments, yielding

$$x_n \approx x_c + e^{ns/x_c}\left[x_0 - x_c + \frac{v\Delta s}{x_c}\left(\frac{\tau_1\tau_2}{\tau_1+\tau_2} - R_{\text{asym}}(t_0)\right)\right], \tag{S34}$$

where R_{asym} is an asymmetric triangular wave:

$$R_{\text{asym}}(t_0) \equiv \begin{cases} 2\tau_2 t_0/(\tau_1+\tau_2) & \text{if } 0 \le t_0 < \tau_1, \\ 2\tau_1(\tau_1+\tau_2-t_0)/(\tau_1+\tau_2) & \text{if } \tau_1 \le t_0 < \tau_1+\tau_2. \end{cases} \tag{S35}$$

The effective fitness boost provided by the tradeoff is therefore

$$s_{\text{eff}}(t_0) \equiv \frac{v\Delta s}{x_c}\left(\frac{\tau_1\tau_2}{\tau_1+\tau_2} - R_{\text{asym}}(t_0)\right), \tag{S36}$$

reducing to Eq. (S20) in the symmetric case.

As before, the influence of the environmental tradeoff is maximized at the beginning of each environmental state, $t_0 = 0$ or $t_0 = \tau_1$. However, the scaling of s_{eff} with τ has been generalized to the harmonic mean of the epoch durations τ_1 and τ_2. If the same total time $\tau_1 + \tau_2$ is split between environments asymmetrically, the specialist advantage will be weakened slightly. Intuitively, the limiting case is a constant environment with $\tau_2 = 0$, where geometric mean fitness $\bar{s}$ coincides with s_1 only and the tradeoff Δs is irrelevant at fixed $\bar{s}$. Despite these nuances, the overall takeaway is that our conclusions do not critically depend on the environmental states having identical durations; instead, environmental asymmetry simply results in a new effective timescale parameter.

2.2 Fixation with Seasonal Drift

In general, environmental conditions may not be perfectly periodic, and can vary stochastically over time. The fate of specialist mutations will be strongly affected by this "seasonal drift." A simple modeling approach to capture this effect is to take the spent in each environment as an i.i.d. random variable with mean τ and variance σ_τ^2. We will generally focus on the regime of modest seasonal drift ($\sigma_\tau \lesssim \tau$) where the switching time is approximately normally distributed, although we will also discuss the impact of higher-variance distributions. (In simulations, we generally draw switching times from a gamma distribution to enforce non-negativity.)

We will find that even modest amounts of stochasticity can have enormous impacts on the specialist fixation probability, with a critical scale of σ_τ where the effects of stochasticity dominate over the deterministic effects discussed in the previous section. As such, we will largely neglect those deterministic effects, focusing on the seasonal drift-dominated regime. Our approach is an extension of the "rolling fitness" analysis discussed above for deterministic environments ($\sigma_\tau = 0$).

bioRxiv preprint doi: https://doi.org/10.64898/2026.09.01.748641; this version posted September 4, 2026. The copyright holder for this preprint (which was not certified by peer review) is the author/funder, who has granted bioRxiv a license to display the preprint in perpetuity. It is made available under a CC-BY 4.0 International license.

2.2.1 *Rolling Fitness with Seasonal Drift*

In this section, we will calculate the fixation probability of specialist mutations in a randomly fluctuating environment. To do so, it is important to consider the relative timescale of founding a new mutation compared to environmental change. If environmental epochs are the longer timescale ($\tau \gg s/v$), then seasonal drift over a single environmental cycle will have significant impacts on a lineage's rolling fitness. If environmental cycles are faster ($\tau \ll s/v$), then the effect of seasonal drift on rolling fitness is not felt until a lineage actually produces a beneficial mutation – meaning that the net effect of seasonal drift over many cycles is the relevant "unit" of stochasticity. However, it will turn out that both regimes give identical results for the fixation probability under the normal approximation we will employ.

Longer environmental cycles. To start, we will assume that environmental epochs are the longer timescale, $\tau \gg s/v$. Consider a lineage with relative fitness $\delta x \equiv x - x_c$ at the start of an environmental cycle. Over the cycle, seasonal drift means the time spent in the beneficial and deleterious environmental states will not perfectly balance out. We can express the impact of this imbalance on rolling fitness by assuming that the lineage takes n_1 founding steps in a beneficial environment, followed by n_2 founding steps in a deleterious environment. Following the recursion relations in Eq. (S12a), its rolling fitness after this process will be

$$\delta x_{n_1+n_2} \approx \delta x_0 \cdot e^{(n_1+n_2)s/x_c} + \Delta s \left(e^{(n_1+n_2)s/x_c} + 1 - 2e^{n_2 s/x_c}\right). \tag{S37}$$

The difference between n_1 and n_2 comes from a random difference $\delta\tau$ between the environmental epoch times. For relatively weak stochasticity $|\delta\tau| \ll \tau$, we can approximate $n_1 + n_2 \approx 2v\tau/s$ and $n_1 - n_2 \approx v\delta\tau/s$. Plugging these in and expanding the exponentials gives us a new recursion relation in terms of $\delta\tau$,

$$\delta x_{K+1} - \delta x_K \approx \frac{2v\tau}{x_c}\left(\delta x_K + \frac{\Delta s}{2\tau}\delta\tau_K\right), \tag{S38}$$

where the capital K indices represent environmental cycles, during which many new fitness classes are founded. We see that Eq. (S38) takes the form of a biased random walk, where the first term on the RHS deterministically pushes δx away from 0, and the second term introduces a random fluctuation $\delta\tau_K$ each environmental cycle. The solution to Eq. (S38) can be expressed as

$$\delta x_K \approx e^{2v\tau K/x_c}\left[\delta x_0 + \xi_K^{\text{long}}(\boldsymbol{\delta\tau})\right], \tag{S39}$$

with ξ a random fitness scale dependent on the realized environmental stochasticity:

$$\xi_K^{\text{long}}(\boldsymbol{\delta\tau}) \equiv \frac{\Delta s v}{x_c}\sum_{J=0}^{K-1} e^{-2v\tau(J+1)/x_c}\delta\tau_J. \tag{S40}$$

bioRxiv preprint doi: https://doi.org/10.64898/2026.09.01.748641; this version posted September 4, 2026. The copyright holder for this preprint (which was not certified by peer review) is the author/funder, who has granted bioRxiv a license to display the preprint in perpetuity. It is made available under a CC-BY 4.0 International license.

Similarly to s_{eff} in the deterministic case, $\xi^{\text{long}}_{K\to\infty}$ acts as an effective boost to the lineage's initial fitness. Incorporating the effects of early jackpot mutations as discussed in SI 2.1.3, the fixation probability is

$$p_{\text{fix}}(\delta x, \xi) \approx \begin{cases} e^{-(x_c+\delta x+\xi)^2/2v - x_c^2/2v} & \text{if } \xi \lesssim -\delta x, \\ 1 & \text{if } \xi > -\delta x. \end{cases} \tag{S41}$$

Thus, the focal lineage is likely to reach fixation if stochasticity boosts the lineage above its nominal break-even fitness. In particular, seasonal drift allows lineages with $\delta x_0 < 0$ to nonetheless have a significant chance of fixation if $|\delta x|$ is comparable to the typical scale of ξ. In other words, a lower-fitness lineage can be brought to fixation by a lucky sequence of longer favorable environments.

Shorter environmental cycles. In the $\tau \ll s/v$ regime, we consider a similar random walk process, but where the timescale of a "step" is now s/v rather than τ. The impact of seasonal drift over this time now includes stochastic effects from many environmental epochs with varying length, yielding a rolling fitness update rule

$$\delta x_{k+1} - \delta x_k \approx \frac{s}{x_c}\left(\delta x_k + \frac{\Delta s}{s/v}\sum_{i=0}^{s/2v\tau-1}\delta\tau_{i+ks/2v\tau}\right) \tag{S42}$$

with solution

$$\delta x_k \approx e^{sk/x_c}\left[\delta x_0 + \xi^{\text{short}}_k(\boldsymbol{\delta\tau})\right], \tag{S43}$$

$$\xi^{\text{short}}_k(\boldsymbol{\delta\tau}) \equiv \frac{\Delta s v}{x_c}\sum_{j=0}^{k-1} e^{-s(j+1)/x_c}\sum_{i=0}^{s/2v\tau-1}\delta\tau_{i+js/2v\tau}. \tag{S44}$$

Besides the new effective value of ξ, the fixation probability exactly maps onto Eq. (S41). Notably, both ξ^{short}_k and ξ^{long}_K have mean zero and the same width at long times:

$$\sigma_\xi \equiv \frac{\Delta s \sigma_\tau}{2\tau}\cdot\sqrt{\frac{2v\tau}{x_c}}. \tag{S45}$$

This width corresponds to the scale of the lineage's *realized* fitness averaged over the $\sim x_c/2v\tau$ cycles it takes to reach fixation. The first factor represents the fitness effect of stochasticity over one environmental cycle, while the second factor attenuates the stochasticity by averaging over the many independent cycles a lineage experiences before fixation.

Averaging over seasonal drift. In both regimes, the overall specialist fixation probability is given by averaging over the background fitness x and the stochasticity-induced fitness shift ξ. Suppose the distribution of ξ is $\rho_{\Delta s}(\xi)$, which depends on Δs through σ_ξ. Then the fixation probability is

$$p_{\text{fix}}(\bar{s}, \Delta s) \approx 2x_c\int_{-x_c+\bar{s}}^{x_c+\bar{s}} dx\frac{e^{-(x-\bar{s})^2/2v}}{\sqrt{2\pi v}}\int_{-x}^{\infty} d\xi\ \rho_{\Delta s}(\xi)\cdot\min\left[e^{[(x+\xi)^2-x_c^2]/2v},\ 1\right]. \tag{S46}$$

bioRxiv preprint doi: https://doi.org/10.64898/2026.09.01.748641; this version posted September 4, 2026. The copyright holder for this preprint (which was not certified by peer review) is the author/funder, who has granted bioRxiv a license to display the preprint in perpetuity. It is made available under a CC-BY 4.0 International license.

While the full distribution of ξ is complicated, it depends on a weighted sum of many independent random variables, so in its bulk it is reasonable to invoke the central limit theorem and approximate $\xi \sim \sigma_\xi \mathcal{N}$ with $\mathcal{N}$ a standard normal random variable. However, integrating out to the tail of ξ represents averaging over many independent environmental realizations, which may not be appropriate for realistic experimental setups where fixations are observed over a limited number of environmental epochs. Furthermore, the extreme tail of ξ is sensitive to the underlying distribution of $\delta\tau$, and is not always well-described by the central limit theorem. A clear limiting case is that the effective benefit ξ can be no more than Δs, the fitness benefit from a permanent beneficial environment. (This is evident from Eq. (S37) before expanding for $|\delta\tau| \ll \tau$.) To account for these effects, it is useful to include an explicit cutoff c for the stochasticity,

$$\rho_{\Delta s}(\xi) \approx \begin{cases} e^{-\xi^2/2\sigma_\xi^2} \Big/ \sqrt{2\pi\sigma_\xi^2} & \text{if } |\xi| < \min(c\sigma_\xi, \Delta s), \\ 0 & \text{otherwise.} \end{cases} \tag{S47}$$

Plugging this into Eq. (S46) reveals that environmental stochasticity can provide a huge boost to the fixation probability for lineages with tradeoffs. This is because lucky environmental stochasticity (large ξ) can allow a lineage to reach fixation even on a lower-fitness background (smaller x), providing an alternate route to fixation that can increase p_{fix} by many orders of magnitude.

When boundary effects in the stochasticity are negligible, then the integral in Eq. (S46) will be dominated by ξ near $(x_c - \bar{s})\sigma_\xi^2/(v + \sigma_\xi^2)$, and we obtain

$$\log p_{\text{fix}}(\bar{s}, \Delta s) \approx -\frac{(x_c - \bar{s})^2}{2(v + \sigma_\xi^2)}, \tag{S48}$$

reducing to the generalist fixation probability when $\sigma_\xi^2 = 0$. If stochasticity is relatively weak, $\sigma_\xi \ll \sqrt{v}$ (equivalently, $\Delta s \cdot \sigma_\tau \ll \sqrt{x_c \tau}$) then the fixation probability simplifies to

$$p_{\text{fix}}(\bar{s}, \Delta s) \approx \sqrt{\frac{2v}{\pi}} \frac{x_c}{\bar{s} + x_c\sigma_\xi^2/v} \exp\left[-\frac{(x_c - \bar{s})^2}{2v} + \frac{(x_c - \bar{s})^2\sigma_\xi^2}{2v^2}\right] \tag{S49}$$

to leading order in the exponent. Thus, the specialist advantage becomes noticeable when the effect of stochasticity over a single cycle is above a critical scale, $\Delta s \cdot \sigma_\tau \gtrsim \sqrt{4v\tau/x_c}$. Since $v\tau/x_c \ll 1$, this scale can be much less than unity, implying that even small levels of seasonal drift strongly influence selection. This counterintuitive effect arises because the possibility of lucky environmental fluctuations expands the range of background fitnesses that have an appreciable chance of reaching fixation. Because fixation is so difficult to begin with in the clonal interference regime, an additional source of stochasticity acts like a strong benefit, even though the environmental fluctuations are not systematically biased toward beneficial environments.

The full numerical integral in Eq. (S46) with the truncated normal approximation in Eq. (S47) is in good agreement with the specialist fixation probability measured from simulations. In particular, simulation results agree with the large-c limit of Eq. (S47), indicating that our

bioRxiv preprint doi: https://doi.org/10.64898/2026.09.01.748641; this version posted September 4, 2026. The copyright holder for this preprint (which was not certified by peer review) is the author/funder, who has granted bioRxiv a license to display the preprint in perpetuity. It is made available under a CC-BY 4.0 International license.

$\sim 10^5$ simulated mutation trajectories sufficiently sample the relevant range of environmental stochasticity. However, this thorough sampling is not required for our qualitative results: even if we restricted our sampling to environments within a single standard deviation of the mean (as might be more realistic for experiments), we still predict that specialist mutations should fix $10^2 - 10^4$ more times as often as generalists (Fig. S1). (For our isocline results in Fig. 6, we find that a cutoff $c = 3$ is most accurate, which nearly saturates the stochasticity in Fig. S1 anyway.)

The effects of seasonal drift will dominate the deterministic specialist advantage in Eq. (S33) when

$$\frac{\sigma_\tau}{\tau} \gtrsim \sqrt{\frac{2v}{\Delta s x_c}}, \tag{S50}$$

meaning that stochastic effects are likely to be relevant even when the overall scale of stochasticity is not very large. Note that our normal approximation sometimes underestimates the fixation probability slightly, likely because our simulated $\delta\tau$ possess wider exponential tails. Even wider-tailed distributions will also make the normal approximation to ξ suspect, as discussed in the next section.

2.2.2 *Higher-Variance Environments*

The normal approximation will be most sensible if fixations are dominated by accumulating a fitness advantage over many independent environmental cycles, rather than a single extremely long environmental state. We will briefly discuss how this assumption depends on the tail statistics of $\delta\tau$, and the properties of the fixation probability in the higher-variance regime.

Suppose that the probability distribution of $\delta\tau$ (the difference in duration between two adjacent environmental states) has a tail which is given by a compressed exponential,

$$\rho(\delta\tau) \sim \exp\left[-(\delta\tau/\sigma_\tau)^\alpha\right]. \tag{S51}$$

This corresponds to a Gaussian when $\alpha = 2$ (used in the normal approximation) and an exponential when $\alpha = 1$ (used in simulations with gamma-distributed times). The probability of drawing an extremely long time difference of at least $\delta\tau^*$ will have a similar scaling $\sim \rho(\delta\tau^*)$, up to algebraic factors.

Now (specializing to $\tau \gg s/v$), we will consider the probability a lower-fitness lineage is set on a deterministic path to fixation by a *single* random walk step with an abnormally large $\delta\tau$. For a lineage which starts at fitness $x_c + \delta x$, this roughly requires $\delta\tau > |\delta x| x_c/\Delta s v$. Integrating over $-\delta x$, we write

$$p_{\text{fix}}^{\text{atyp}} \sim \int_0^{\Delta s} e^{-(x_c - s - \delta x)^2/2v} \rho\left(\frac{x_c}{\Delta s v}\delta x\right) d\delta x \tag{S52}$$

$$\sim \int_0^{\Delta s} \exp\left[-\frac{x_c^2}{2v} + \frac{x_c s}{v} + \frac{x_c}{v}\delta x - \left(\frac{x_c}{\Delta s v \sigma_\tau}\delta x\right)^\alpha\right] d\delta x. \tag{S53}$$

bioRxiv preprint doi: https://doi.org/10.64898/2026.09.01.748641; this version posted September 4, 2026. The copyright holder for this preprint (which was not certified by peer review) is the author/funder, who has granted bioRxiv a license to display the preprint in perpetuity. It is made available under a CC-BY 4.0 International license.

When $\alpha > 1$, the integral will have a saddle point at $\delta x^* \sim (v/x_c)(\Delta s\sigma_\tau)^{\alpha/(\alpha-1)}$ (up to α-dependent prefactors), yielding

$$p_{\mathrm{fix}}^{\mathrm{atyp}} \sim \exp\left[-\frac{x_c^2}{2v} + \frac{x_c s}{v} + (\Delta s\sigma_\tau)^{\frac{\alpha}{\alpha-1}}\right]. \tag{S54}$$

when $\Delta s\sigma_\tau^\alpha < (x_c/v)^{(\alpha-1)}$. For larger stochasticity, the integral will be dominated by its upper bound, yielding

$$p_{\mathrm{fix}}^{\mathrm{atyp}} \sim \exp\left[-\frac{x_c^2}{2v} + \frac{x_c(s+\Delta s)}{v} - \left(\frac{x_c}{v\sigma_\tau}\right)^\alpha\right]. \tag{S55}$$

This atypical path to fixation entails an effective fitness benefit of $\sim (v/x_c)(\Delta s\sigma_\tau)^{\alpha/(\alpha-1)}$, generically weaker than the normal approximation unless α is close to unity and $\Delta s\sigma_\tau \gtrsim 1$.

For $\alpha = 1$ (an exponential tail), the relevance of rare environmental cycles is marginal, depending on the size of $\Delta s\sigma_\tau$. When $\Delta s\sigma_\tau < 1$, randomness across many environmental cycles will dominate; when $\Delta s\sigma_\tau > 1$, the most likely path to fixation will be lineages $\sim \Delta s$ below the break-even fitness experiencing an abnormally long beneficial environment. This effect likely explains the fact that the normal approximation can underestimate the specialist fixation probability in simulations (Fig. 3C), where $\Delta s\sigma_\tau \gtrsim 1$. However, the quantitative predictions are still broadly reasonable even up to $\Delta s\sigma_\tau \sim 10$, likely because the effective fitness benefit is capped at Δs in both cases, yielding similar results for higher stochasticity.

For wider-tailed distributions (e.g. $\alpha < 1$), the normal approximation is suspect, but may still apply for sufficiently small $\Delta s\sigma_\tau$. We consider a simple high-variance distribution in Fig. S2, where rare environmental states last an extremely long time. In this case, the fixation probability is dominated by the probability of these rare environmental states, where specialist mutations experience an effective benefit of Δs.

We emphasize that the approach taken in this section is critically dependent on the assumption that specialists are rare, such that only one lineage at a time can take advantage of environmental fluctuations‘. Outside of this regime, many lineages can simultaneously respond to environmental fluctuations, and their fixation advantage will be reduced. We explore this effect in SI 3.3.

3 Dynamics with Frequent Specialists

3.1 Introduction

Our analysis so far has focused on the case where specialist mutations with $\Delta s > 0$ arise rarely, so that only one specialist lineage is present in the population at any given time. In this section, we will relax this strong assumption, using heuristic methods to capture an overall picture of how the fates of mutations are determined in a population where specialist mutations are common. Relative to our discussion of rare specialists, this analysis will

bioRxiv preprint doi: https://doi.org/10.64898/2026.09.01.748641; this version posted September 4, 2026. The copyright holder for this preprint (which was not certified by peer review) is the author/funder, who has granted bioRxiv a license to display the preprint in perpetuity. It is made available under a CC-BY 4.0 International license.

focus on a more specialized regime, and neglect some quantitative details which would be compelling to further explore in future work. However, our approach will nonetheless reveal many of the most interesting ways frequent specialist mutations influence population dynamics, and allow for qualitative and quantitative agreement with key features of simulations.

When the specialist mutation rate is large, $NU_s \gg 1$, the background population will adapt just like the focal strain does, forming a fully two-dimensional fitness distribution. We parametrize this distribution in terms of geometric mean fitness $X_\parallel$ and specialism $X_\perp$, defined such that a mutation with tradeoff strength Δs shifts $X_\perp$ to $X_\perp + \Delta s$. In the time-symmetric case $\tau_1 = \tau_2$ (which we assume henceforth), the instantaneous fitness of a genotype is simply $X_\parallel + I(t) \cdot X_\perp$. Its relative fitness is given by $x_\parallel \equiv X_\parallel - \overline{X}_\parallel$ and $x_\perp \equiv X_\perp - \overline{X}_\perp$, where bars represent the population mean. We make a linear ansatz for how these population averages vary with time:

$$\frac{d\overline{X}_\parallel}{dt} = v_\parallel, \tag{S56a}$$

$$\frac{d\overline{X}_\perp}{dt} = v_\perp \cdot I(t). \tag{S56b}$$

Under this ansatz, the instantaneous mean fitness of the background population grows at rate $v_\parallel + v_\perp$ as it specializes to one environment, but drops discontinuously when the environment switches, such that the long-term rate of adaptation is only $v_\parallel$. The "rate of specialization" $v_\perp$ represents cyclic specialization to the current environment which is lost in the next environmental state. Both $v_\parallel$ and $v_\perp$ are now unknown parameters which must be determined self-consistently. In particular, $v_\parallel$ may not take the same value as v in the rare-specialists case, although we will find that they agree to leading order.

The dynamics in Eq. (S56) can be thought of as a linear approximation to the true dynamics of the bulk population, which is sufficient to estimate the key behavior to leading order. One neglected feature of the dynamics is that over long timescales where multiple specialist mutations fix, the number of specialist mutations of each type will not perfectly balance, so $\overline{X}_\perp$ will drift stochastically from its original value. However, because fitness is always measured relative to the rest of the population, this drift should have minimal effects over the timescale of a single mutation reaching fixation or going extinct.

The next key effect of allowing frequent specialist mutations is that any focal lineage has access to multiple types of mutations, which change environmental specialism in addition to average fitness. This means that the fate of a mutation must be determined by aggregating over all the possible mutations its descendants could acquire. We can resolve this complexity by noting that only a small fraction of these descendants are likely to reach fixation – those that follow the "optimal mutational path," which depends on environmental fluctuations. In our analysis of rare specialists, we found that that specialist mutations aligned with the current environment lose their advantage halfway through an environmental state, so we might expect this optimal path to switch the type of mutation it gets at roughly the same time. Our rolling fitness framework will allow us to formalize this intuition.

bioRxiv preprint doi: https://doi.org/10.64898/2026.09.01.748641; this version posted September 4, 2026. The copyright holder for this preprint (which was not certified by peer review) is the author/funder, who has granted bioRxiv a license to display the preprint in perpetuity. It is made available under a CC-BY 4.0 International license.

3.2 Frequent Specialists in a Periodic Environment

We will first consider specialist mutations in a perfectly periodic environment ($\sigma_\tau = 0$). For ease of presentation, we will start with the symmetric case $U_s = U_g$ and $\bar{s} = s$, where specialist mutations arise at the same rate as generalists and have the same average fitness effect. The more general case is discussed in SI 3.2.7. We will also specialize to the $\tau \gg s/v_\parallel$ regime, where many new fitness classes are founded each environmental cycle. (Recall that our main results in the rare-specialists regime did not critically depend on this choice, but were often more dramatic for larger τ, making this regime easier to explore in simulations.) Finally, we will see that the frequent-specialists regime is sensitive to the relative sizes of Δs and s, which determines whether specialist mutations founded in the "wrong" environment have a higher initial growth rate than their parent strain. We will focus on the $s > \Delta s$ case in our analytic theory, but discuss the $\Delta s > s$ case in SI 3.2.8.

3.2.1 *Rolling Fitness of a Specializing Lineage*

Consider a lineage which establishes at time t_0 at relative geometric mean fitness $x_\parallel$ and relative specialism $x_\perp$. Its frequency trajectory will be

$$f(x_\parallel, x_\perp, t_0, t) \approx \frac{1}{2N(x_\parallel + x_\perp I(t_0))} e^{x_\parallel t - v_\parallel t^2/2 + x_\perp [R(t+t_0) - R(t_0)] - v_\perp [R(t+t_0) - R(t_0)]^2/2} \quad \text{(S57)}$$

We can follow the same procedure as in SI 2.1.2 to calculate the typical time it takes this lineage to produce a mutant,

$$\begin{aligned} T &\approx \frac{s}{v_\parallel}\left(1 - \frac{x - x_c}{x_c}\right) - \frac{\Delta R(x_\perp - v_\perp \Delta R/2)}{x_c} \\ &\quad + \frac{x - x_c - s}{v_\parallel x_c^2}\left[s(x - x_c) + v_\parallel \Delta R(x_\perp - v_\perp \Delta R/2)\right], &\text{(S58)} \\ &\approx \frac{s}{v_\parallel}\left(1 - \frac{x - x_c}{x_c}\right) - \frac{s x_\perp I(t_0)}{v_\parallel x_c} + \frac{s^2 v_\perp}{2 v_\parallel^2 x_c} &\text{(S59)} \end{aligned}$$

where $\Delta R \equiv R(T + t_0) - R(t_0) = I(t_0) \cdot T$ in the $\tau \gg s/v_\parallel$ regime. This equation reduces to Eq. (S16) for $x_\perp = \Delta s$ and $v_\perp = 0$. Importantly, we have defined x_c as a large fitness scale analogous to Eq. (S3),

$$x_c = \frac{v_\parallel}{s} \log\left(\frac{s}{U_s + U_g}\right) + \frac{s}{2}. \quad \text{(S60)}$$

Note that our perturbative expansion of T assumes $x_\perp \ll x_c$, which may not be valid if Δs is sufficiently large. We will compare relevant values of $x_\perp$ to x_c later, and find that they are generally smaller than x_c if $s > \Delta s$.

When the next fitness class is founded, the update rule for $x_\parallel$ is the same as before, but now $x_\perp$ can also change due to adaptation in the rest of the population, and new specialist

bioRxiv preprint doi: https://doi.org/10.64898/2026.09.01.748641; this version posted September 4, 2026. The copyright holder for this preprint (which was not certified by peer review) is the author/funder, who has granted bioRxiv a license to display the preprint in perpetuity. It is made available under a CC-BY 4.0 International license.

mutations in the focal lineage. The resulting update rules are

$$x_{\parallel}^{k+1} \approx x_{\parallel}^{k} + s - v_{\parallel} T_k, \tag{S61a}$$

$$x_{\perp}^{k+1} \approx x_{\perp}^{k} + \Delta s \cdot D_k - \frac{v_{\perp} s}{v_{\parallel}} I(t_k), \tag{S61b}$$

where $D_k \in (-1, 0, 1)$ parametrizes the type of the new mutation: 0 for a generalist, and ± 1 for each type of specialist. Crucially, these update rules are only valid when $s > \Delta s$, which guarantees that each new fitness class is dominated by descendants of the *first* mutation into that class (because daughter strains always grow faster than their parent). Under this assumption, the solution to these recursive relations is

$$x_{\parallel}^{n} \approx x_c + e^{ns/x_c} \left[x_{\parallel}^{0} - x_c + \frac{s}{x_c} \sum_{i=0}^{n-1} e^{-(i+1)s/x_c} \left(x_{\perp}^{i} \cdot I\left(t_i\right) - \frac{s v_{\perp}}{2 v_{\parallel}} \right) \right], \tag{S62a}$$

$$x_{\perp}^{n} \approx x_{\perp}(0) + \Delta s \sum_{i=0}^{n-1} D_i - \frac{v_{\perp} s}{v_{\parallel}} \sum_{i=0}^{n-1} I(t_i). \tag{S62b}$$

It is useful to plug in $x_{\perp}^{i}$ explicitly and separate out the portion of $x_{\parallel}^{n}$ dependent on the mutational path,

$$x_{\parallel}^{n} \approx \frac{s \Delta s}{x_c} \sum_{i=1}^{n-1} D_{i-1} \sum_{j=0}^{n-i-1} e^{(n-i-j-1)s/x_c} I(t_{i+j}) + \ldots \tag{S63}$$

with omitted terms independent of $\boldsymbol{D}$.

3.2.2 Optimal Mutational Path

Each combination of D_i represents a subset of the focal lineage that has taken a particular mutational path (i.e., a specific order of types of mutations). However, we expect the fixation probability of the lineage to be dominated by a particular mutational path which maximizes long-term fitness. Since Eq. (S63) depends on each D_i linearly, we can write this optimal path directly:

$$D_i^*(n) \approx \text{sign} \left[\sum_{j=0}^{n-i-2} e^{-js/x_c} I(t_{i+j+1}) \right] \tag{S64}$$

We see that D_i^* is based on a weighted sum of the environmental states between t_i and t_{n-1}, with more importance placed on the times soon after the mutation established. Crucially, Eq. (S64) depends on n, the “observation time” of the fitness $x_{\parallel}^{n}$. This dependence indicates that what appears to be optimal at one time may not be optimal over the long term. For example, a mutation acquired near the end of a favorable environment may appear optimal at first, but quickly be outcompeted by other mutational paths when the environment changes.

bioRxiv preprint doi: https://doi.org/10.64898/2026.09.01.748641; this version posted September 4, 2026. The copyright holder for this preprint (which was not certified by peer review) is the author/funder, who has granted bioRxiv a license to display the preprint in perpetuity. It is made available under a CC-BY 4.0 International license.

By plugging in the periodic structure of $I(t)$, we can express the sum in Eq. (S64) as a term going through $n_{\text{cyc}} \equiv \text{floor}\left[(n-i-1)/(2v_{\parallel}\tau/s)\right]$ complete cycles, followed by an "overhang" term based on the phase difference between t_i and t_k:

$$D_i^*(n) \approx \text{sign}\left[\frac{\tau}{2} - R(t_{i+1}) + e^{-n_{\text{cyc}}(i,n)\cdot 2v_{\parallel}\tau/x_c}\left(R(t_n) - \frac{\tau}{2}\right)\right]. \tag{S65}$$

We see that the optimal mutation depends on the time the mutation establishes (t_i) as well as the observation time (t_n), with the importance of the latter term decaying if the mutation happened many cycles in the past. When this term is negligible ($n_{\text{cyc}} \gtrsim x_c/2v_{\parallel}\tau$), the optimal mutational path reduces to the *long-term optimal* mutational path:

$$D_i^*(n \to \infty) = \text{sign}\left[\frac{\tau}{2} - R(t_{i+1})\right], \tag{S66}$$

reflecting the impact of a mutation many environmental cycles into the future. This exactly mirrors our intuition from the rare-specialists case: it is best to acquire mutations favoring the current environment only until it is halfway over. Afterward, it is best to switch to mutations favoring the upcoming environment.

Effective fitness shift. The long-term fixation probability of a lineage following the optimal mutational path will be determined by its initial mean fitness $x_{\parallel}^0$ plus a fitness shift ζ given by the last term in the brackets of Eq. (S62a):

$$\zeta\left(x_{\perp}^0, t_0\right) \approx \lim_{n\to\infty} \frac{s}{x_c} \sum_{i=0}^{n-1} e^{-(i+1)s/x_c}\left(x_{\perp}^i(\boldsymbol{D}^*)\cdot I\left(t_i\right) - \frac{sv_{\perp}}{2v_{\parallel}}\right), \tag{S67}$$

$$\approx \frac{v_{\parallel}\tau\Delta s}{4s} + x_{\perp}^0\frac{v_{\parallel}}{x_c}\left[\frac{\tau}{2} - R(t_0)\right] - \frac{v_{\parallel}v_{\perp}}{2x_c}\left[\frac{\tau}{2} - R(t_0)\right]^2 - \frac{v_{\parallel}v_{\perp}\tau^2}{24x_c} + \mathcal{O}\left(\frac{v_{\parallel}^2\tau^2\Delta s}{sx_c}\right) \tag{S68}$$

This choice of neglected terms will be valid if $v_{\perp} \gg v\Delta s/s$, which we will verify later. The first constant correction represents the effective benefit that a lineage acquires by following the optimal mutational path, providing a benefit proportional to Δs (the tradeoff strength of available specialist mutations). The $x_{\perp}^0$-dependent term has exactly the same form as the rare-specialists regime, representing the time-dependent benefit (or cost) a lineage has from having specialized already. Finally, the $v_{\perp}$-dependent terms represent the cost of competing against a specializing background population. Note that this cost is minimized in the middle of an environmental state, where the optimal path begins to diverge from the direction the background population is specializing.

3.2.3 Two-Dimensional Traveling Wave

In order to estimate the fixation probability of a mutation, we must characterize the two-dimensional fitness distribution of the population $f(x_{\parallel}, x_{\perp})$. We will find that f is a two-dimensional traveling wave which moves according to Eq. (S56). Our central approach is to realize that all extant organisms in the population must be descended from a common

bioRxiv preprint doi: https://doi.org/10.64898/2026.09.01.748641; this version posted September 4, 2026. The copyright holder for this preprint (which was not certified by peer review) is the author/funder, who has granted bioRxiv a license to display the preprint in perpetuity. It is made available under a CC-BY 4.0 International license.

ancestor in the distant past, whose dynamics when small were given by the rolling fitness calculation we just performed. Thus, our update rules for a focal lineage can be used to self-consistently determine the entire two-dimensional fitness distribution of the population. In the process of performing this calculation, we will also be able to estimate the rate of specialization $v_\perp$, which we address first.

Rate of specialization. The rate of specialization is encoded in the optimal mutational path we found earlier, Eq. (S65). This path corresponds to the organisms at the highest frequency in the high-fitness nose of the population at time t_n, which will eventually rise in frequency until they form the bulk of the wave. The specialism $x_\perp$ of this genotype can be inferred by aggregating over all the mutations it has gained:

$$D_{\text{tot}}(n) \equiv \sum_{i=0}^{n-2} D_i^*(n), \tag{S69}$$

$$\approx \frac{2x_c}{s\tau}\left(R(t_n) - \frac{\tau}{2}\right) \cdot \left(1 - e^{-ns/x_c}\right) + \mathcal{O}\left(\frac{v_\parallel \tau}{s}\right) \tag{S70}$$

since $n \sim x_c/s \gg x_c/2v_\parallel\tau$ is large enough to span many environmental cycles. Crucially, this sum is highly sensitive to the observation time t_n: unlike the long-term optimal mutational path, the short-term optimal mutational path entails strongly specializing toward the *current* environment. We see that the average specialism in the high-fitness nose changes at rate $\Delta s \cdot 2x_c/s\tau$. We will show that this rate of change in the nose feeds back to set the rate of specialization in the bulk,

$$v_\perp \approx \left(1 - e^{-1}\right) \cdot \Delta s \cdot \frac{2x_c}{s\tau}, \tag{S71}$$

taking $n = x_c/s$ to cut off the sum at a coalescence time, which improves agreement with simulations (Fig. S3B). Thus, longer environmental cycles tend to limit the diversity in environmental preferences present in the population, lowering $v_\perp$ according to Fisher's Fundamental Theorem. Because $v_\parallel\tau/x_c \ll 1$, the asymptotic limit of $v_\perp$ is large enough to satisfy our assumption that $v_\perp \gg \Delta s v_\parallel/s$.

Simulations verify this rough dependence of $v_\perp$ on Δs and τ, although its value quantitatively approaches Eq. (S71) only for extremely large population sizes when both $v_\parallel\tau/x_c$ and $s/v_\parallel\tau$ are very small (Fig. S3). Simulated values of $v_\perp$ are generally less than the asymptotic limit, meaning that it acts as an upper bound. In practice, we often measure $v_\perp$ from simulations and treat it as an input rather than relying on this leading-order analytic result.

Frequency distribution. We will now describe the frequency distribution of the population as a whole, showing that it is consistent with the rate of specialism we found in Eq. (S71). The peak of this frequency distribution in the nose is given by the optimal path in Eq. (S70), which predicts that the peak relative specialism $x_\perp^*$ should obey

$$x_\perp^*(t) = \left(\frac{2x_c\Delta s}{s\tau} - v_\perp\right)\left(R(t) - \frac{\tau}{2}\right), \tag{S72}$$

bioRxiv preprint doi: https://doi.org/10.64898/2026.09.01.748641; this version posted September 4, 2026. The copyright holder for this preprint (which was not certified by peer review) is the author/funder, who has granted bioRxiv a license to display the preprint in perpetuity. It is made available under a CC-BY 4.0 International license.

where we are once again treating $v_\perp$ as an unknown parameter. We would like to characterize not only the location of this peak, but also the shape of the frequency profile in the nose near $x_\perp^*$. To do so, we must extend Eq. (S63) to consider deviations from the optimal mutational path, which will form the frequency profile around the peak.

The coefficient of D_i in Eq. (S63) can be written as

$$c_i \approx \frac{v_\parallel \Delta s}{x_c} e^{(n-i)s/x_c} \left[\frac{\tau}{2} - R(t_{i+1}) + e^{-n_{\mathrm{cyc}} 2 v_\parallel \tau / x_c} \left(R(t_n) - \frac{\tau}{2}\right)\right]. \tag{S73}$$

The rolling fitness cost of sign-flipping D_i from its optimal value is $2c_i$. To find the least costly way of flipping a fixed number M of mutations, we define a fitness threshold z and flip all mutations with a cost of less than z per mutation. Over a single complete cycle n_{cyc} cycles before the present, this will result in flipping

$$M(z) = \frac{z x_c}{s \Delta s} e^{-n_{\mathrm{cyc}} 2 v_\parallel \tau / x_c} \tag{S74}$$

mutations. The total fitness cost of flipping these mutations will be

$$2 \sum_{m=1}^{M/2} \frac{2 m s \Delta s}{x_c} e^{n_{\mathrm{cyc}} 2 v_\parallel \tau / x_c} \approx \frac{M^2 s \Delta s}{2 x_c} e^{n_{\mathrm{cyc}} 2 v_\parallel \tau / x_c} \approx \frac{z^2 x_c}{2 s \Delta s} e^{-n_{\mathrm{cyc}} 2 v_\parallel \tau / x_c}. \tag{S75}$$

Aggregating over all cycles (i.e., summing over n_{cyc}) yields a total number of flips

$$M_{\mathrm{tot}} = \frac{z x_c^2}{2 s v_\parallel \tau \Delta s}, \tag{S76}$$

resulting in a total change in specialism

$$|x_\perp - x_\perp^*| = 2 \Delta s M_{\mathrm{tot}} = \frac{z x_c^2}{s v_\parallel \tau}, \tag{S77}$$

at a rolling fitness cost

$$x_\parallel(x_\perp^*) - x_\parallel(x_\perp) = \frac{z^2 x_c^2}{4 s v_\parallel \tau \Delta s} = \frac{s v_\parallel \tau}{4 x_c^2 \Delta s} |x_\perp - x_\perp^*|^2 \tag{S78}$$

relative to the optimal path, where we have rewritten dependence on z in terms of $|x_\perp - x_\perp^*|$. These equations describe how the rolling fitness $x_\parallel$ of organisms in the nose depends on their environmental specialism $x_\perp$.

Next, we will change variables in Eq. (S78) from $x_\parallel$ to frequency in the population. A lineage whose rolling fitness is $\delta x_\parallel$ larger than another's must have established $(\delta x_\parallel)/v_\parallel$ generations earlier. Lineages at the nose of the wave will have relative fitness near x_c, rising in frequency as $\sim e^{x_c t}$ after they establish in the population. So, rolling fitness can be converted to frequency via the relation

$$d \log f \sim \frac{x_c}{v_\parallel} d x_\parallel. \tag{S79}$$

bioRxiv preprint doi: https://doi.org/10.64898/2026.09.01.748641; this version posted September 4, 2026. The copyright holder for this preprint (which was not certified by peer review) is the author/funder, who has granted bioRxiv a license to display the preprint in perpetuity. It is made available under a CC-BY 4.0 International license.

The frequency profile of organisms in the high-fitness nose is therefore

$$f(x_\perp^{\text{est}}, t_0) \sim \exp\left[-\frac{s\tau}{4x_c\Delta s}\left(x_\perp^{\text{est}} - x_\perp^*(t_0)\right)^2\right], \tag{S80}$$

where $x_\perp^{\text{est}}$ is the relative specialism of a the lineage at the time it establishes. After establishing in the nose of the wave at average fitness $x_\parallel^{\text{est}}$ and specialism $x_\perp^{\text{est}}$, a lineage will grow in frequency over time. Its frequency t generations after establishing will be

$$\begin{aligned} f(x_\parallel^{\text{est}}, x_\perp^{\text{est}}, t_0, t) \sim \exp\Bigg[&-\frac{s\tau}{4x_c\Delta s}\left(x_\perp^{\text{est}} - x_\perp^*(t_0)\right)^2 + x_\parallel^{\text{est}} t - \frac{v_\parallel}{2}t^2 \\ &+ x_\perp^{\text{est}}\left(R(t+t_0) - R(t_0)\right) - \frac{v_\perp}{2}\left(R(t+t_0) - R(t_0)\right)^2\Bigg]. \end{aligned} \tag{S81}$$

Finally, we change variables from establishment fitness to instantaneous relative fitness, substituting $t \to (x_\parallel^{\text{est}} - x_\parallel)/v$ and $x_\perp^{\text{est}} \to x_\perp + v_\perp\left[R(t) - R(t_0)\right]$. This yields

$$f(x_\parallel, x_\perp, t_0, t) \sim \exp\left[-\frac{x_\parallel^2}{2v_\parallel} - \frac{s\tau x_\perp^2}{4x_c\Delta s} + x_\perp \Delta R(t, t_0)\left(1 - \frac{s\tau v_\perp}{2x_c\Delta s}\right) + \frac{s\tau x_\perp x_\perp^*}{2x_c\Delta s} + \dots\right],$$

where the omitted terms are independent of $x_\parallel$ and $x_\perp$ but may depend on $x_\perp^*$ and t_0. We observe that if $v_\perp$ follows Eq. (S70), the explicit time dependence will cancel out, and $x_\perp^*$ will be fixed at zero. This simplification leaves only

$$f(x_\parallel, x_\perp) \sim \exp\left(-\frac{x_\parallel^2}{2v_\parallel} - \frac{s\tau x_\perp^2}{4x_c\Delta s}\right) \tag{S82}$$

$$f(x_\parallel, x_\perp) = \frac{1}{\sqrt{2\pi v_\parallel v_\perp}} e^{-x_\parallel^2/2v_\parallel - x_\perp^2/2v_\perp} \tag{S83}$$

Thus, the two-dimensional traveling wave has a simple stationary Gaussian profile consistent with our original ansatz in Eq. (S56) and our expression for $v_\perp$ in Eq. (S71). Note that because $x_\perp$ is measured relative to the population mean, it is stationary in time even though the absolute specialism of the population fluctuates as the environment changes.

3.2.4 *Jackpot Mutations in the Frequent-Specialist Regime*

Now that we have characterized the fitness distribution present in the population, we would like to estimate the fixation probability of a mutant. This requires accounting for the probability that a lineage obtains an abnormally early "jackpot" mutation. This analysis will closely follow the rare-specialist analysis in SI 2.1.3 and leads to essentially the same results, where a mutation's fixation probability sharply decreases as $x_\parallel$ falls below $x_c - \zeta$.

To incorporate stochasticity in founding time, we write the rate of establishing beneficial mutations t generations after initial establishment as

$$n_{\text{est}}(x_\parallel^0, x_\perp^0, t_0, t) \approx U \exp\left[(x_\parallel^0 \pm x_\perp^0)t - \frac{v_\parallel + v_\perp}{2}t^2\right], \tag{S84}$$

bioRxiv preprint doi: https://doi.org/10.64898/2026.09.01.748641; this version posted September 4, 2026. The copyright holder for this preprint (which was not certified by peer review) is the author/funder, who has granted bioRxiv a license to display the preprint in perpetuity. It is made available under a CC-BY 4.0 International license.

assuming that t_0 and t are in the same environmental state, which determines the sign of $x_\perp^0$ chosen. Note that we assume $U = U_s = U_g$ (see SI 3.2.7 for the extension to differing mutation rates).

We first change variables from time to establishment fitness $x_\parallel^1 = x_\parallel^0 + s - v_\parallel t$, and then to $\chi = x_\parallel + \zeta(x_\perp, t_0) - x_c$. This gives the probability an optimally-specializing lineage jackpots from χ_0 to χ_1:

$$p_{\text{jackpot}}(\chi_1|\chi_0, t_0, x_\perp^0) \approx \frac{s}{v_\parallel} \exp\left[-\frac{x_c s}{v_\parallel} + \frac{s^2}{2v_\parallel} \right.$$
$$+ \frac{1}{v_\parallel}\left(\chi_0 + x_c - \zeta(x_\perp^0, t_0) + I(t_0)x_\perp^0\right)\left(\chi_0 - \chi_1 + \zeta(x_\perp^1, t_1) - \zeta(x_\perp^0, t_0) + s\right)$$
$$\left. - \frac{v_\parallel + v_\perp}{2v_\parallel^2}\left(\chi_0 - \chi_1 + \zeta(x_\perp^1, t_1) - \zeta(x_\perp^0, t_0) + s\right)^2 \right]. \tag{S85}$$

A lineage starting at the break-even fitness, $\chi_0 = 0$, will tend to land at the break-even fitness if it does not jackpot. Thus, the argument of the exponential should roughly cancel when $\chi_0 = \chi_1 = 0$, so we can ignore terms independent of χ_0 and χ_1. Next, we observe that $x_\perp^1$ and t_1 have implicit dependence on $\Delta\chi \equiv \chi_1 - \chi_0$ through

$$x_\perp^1 = x_\perp^0 + \Delta s D - I(t_0)\frac{v_\perp}{v_\parallel}\left(-\Delta\chi + \zeta(x_\perp^1, t_1) - \zeta(x_\perp^0, t_0) + s\right), \tag{S86}$$

$$t_1 = t_0 + \frac{1}{v_\parallel}\left(-\Delta\chi + \zeta(x_\perp^1, t_1) - \zeta(x_\perp^0, t_0) + s\right). \tag{S87}$$

We can account for this dependence using a Taylor approximation, assuming that the jackpot is not too far from the typical waiting time for a mutation:

$$\zeta(x_\perp^1, t_1) \approx \zeta(x_\perp^{1,\text{typ}}, t_1^{\text{typ}}) + \Delta\chi\left[I(t_0)\frac{v_\perp}{v_\parallel}\frac{\partial\zeta}{\partial x_\perp^1} - \frac{1}{v_\parallel}\frac{\partial\zeta}{\partial t_1}\right]\Bigg|_{x_\perp^{1,\text{typ}}, t_1^{\text{typ}}}, \tag{S88}$$

where $x_\perp^{1,\text{typ}}$ and t_1^{typ} are the non-jackpotting typical values of $x_\perp^1$ and t_1, independent of χ_0 and χ_1. With these simplifications, we can write the leading terms in the jackpotting probability as

$$p_{\text{jackpot}}(\chi_1|\chi_0, t_0, x_\perp^0) \approx \frac{s}{v_\parallel}\exp\left[\frac{x_c}{v_\parallel}(\chi_0 - \chi_1) + \frac{\chi_0^2 - \chi_1^2 + 2s\chi_1}{2v_\parallel} + \frac{\chi_0 - \chi_1}{v_\parallel}\left(-\frac{v_\parallel \Delta s \tau}{4s} \right.\right.$$
$$\left.\left. + I(t_0)x_\perp^0 - \frac{v_\perp}{2v_\parallel}(\chi_0 - \chi_1) - I(t_0)\frac{v_\perp x_c}{v_\parallel}\frac{\partial\zeta}{\partial x_\perp} + \frac{x_c}{v_\parallel}\frac{\partial\zeta}{\partial t_0}\right)\right]. \tag{S89}$$

The derivatives of ζ are, to leading order,

$$\frac{\partial\zeta}{\partial x_\perp} \approx \frac{v_\parallel}{x_c}\left(\frac{\tau}{2} - R(t_0)\right), \tag{S90}$$

$$\frac{\partial\zeta}{\partial t_0} \approx -\frac{v_\parallel x_\perp}{x_c}I(t_0) + \frac{v_\parallel v_\perp}{x_c}I(t_0)\left(\frac{\tau}{2} - R(t_0)\right) \tag{S91}$$

bioRxiv preprint doi: https://doi.org/10.64898/2026.09.01.748641; this version posted September 4, 2026. The copyright holder for this preprint (which was not certified by peer review) is the author/funder, who has granted bioRxiv a license to display the preprint in perpetuity. It is made available under a CC-BY 4.0 International license.

resulting in an expression identical to the rare-specialists case in Eq. (S23):

$$p_{\text{jackpot}} \approx \frac{s}{v_\parallel} \exp\left[\frac{x_c}{v_\parallel}(\chi_0 - \chi_1) + \frac{\chi_0^2 - \chi_1^2 + 2s\chi_1}{2v}\right]. \tag{S92}$$

As discussed in SI 2.1.3, this results in lineages with $\chi < 0$ surviving with probability

$$p(\chi) \approx \exp\left(\frac{\chi(2x_c + \chi)}{2v_\parallel}\right), \tag{S93}$$

$$\approx \exp\left[\frac{x_\parallel^2 - x_c^2}{2v_\parallel} + \frac{\Delta s \tau}{4s} x_c - \frac{v_\perp \tau^2}{24} + x_\perp\left(\frac{\tau}{2} - R(t_0)\right) - \frac{v_\perp}{2}\left(\frac{\tau}{2} - R(t_0)\right)^2\right] \tag{S94}$$

after returning to untransformed fitness units, with neglected terms of $\mathcal{O}\left(\Delta s v_\parallel \tau^2/s\right)$ in the exponent.

3.2.5 Overall Fixation Probability

We can now calculate the fixation probability of a general mutation $p_{\text{fix}}(\bar{s}, \delta s)$ based on our estimate for the fixation probability of an established lineage on starting fitness and time, Eq. (S94), and the background fitness distribution, Eq. (S83). Note that we use $\bar{s}$ and δs to describe the geometric mean fitness effect and tradeoff strength of the focal mutation, which may differ from the corresponding parameters (s and Δs) in the JDFE describing the other mutations available in the population. The fixation probability is given by an integral over backgrounds and time:

$$p_{\text{fix}}(\bar{s}, \delta s) \approx \int_0^{2\tau} \frac{dt_0}{2\tau} \int_{-x_c}^{x_c} dx_\perp \int_{-x_c}^{x_c} dx_\parallel\, 2x_c \cdot f(x_\parallel, x_\perp) \cdot p(x_\parallel + \bar{s}, x_\perp + \delta s, t_0) \tag{S95}$$

The integral over $x_\parallel$ is dominated by $x_\parallel$ within $\sim v_\parallel/s$ of $x_c - \bar{s} - \zeta$, while the integral over $x_\perp$ is dominated by $x_\perp$ within $\sim \sqrt{v_\perp}$ of

$$x_\perp^*(t_0) \equiv v_\perp\left(\frac{\tau}{2} - R(t_0)\right). \tag{S96}$$

The overall time-dependent expression after performing these integrals is

$$p_{\text{fix}}(\bar{s}, \delta s \mid t_0) \approx \frac{x_c}{\bar{s} + \zeta(x_\perp^*, t_0)} \sqrt{\frac{2v_\parallel}{\pi}} \exp\left[-\frac{(x_c - \bar{s})^2}{2v_\parallel} + \delta s\left(\frac{\tau}{2} - R(t_0)\right) + \frac{x_c \tau \Delta s}{4s} - \frac{v_\perp \tau^2}{24} + \mathcal{O}\left(\frac{v_\parallel \tau^2 \Delta s}{s}\right)\right], \tag{S97}$$

where the size of omitted terms in the exponent is set by our truncation of ζ in Eq. (S68), and may depend on t_0 but not $\bar{s}$ and δs to leading order.

Two conclusions immediately follow. First, $x_\perp^*(t_0)$ effectively cancels out the bulk population's environmental specialization over a cycle. This means that near environmental

bioRxiv preprint doi: https://doi.org/10.64898/2026.09.01.748641; this version posted September 4, 2026. The copyright holder for this preprint (which was not certified by peer review) is the author/funder, who has granted bioRxiv a license to display the preprint in perpetuity. It is made available under a CC-BY 4.0 International license.

switches, the genetic backgrounds of typical successful mutations will be "pre-adapted" to the upcoming environment relative to typical organisms. Second, the δs-dependence of p_{fix} has exactly the same form as the rare-specialists regime,

$$\log\left(\frac{p_{\text{fix}}(\bar{s},\delta s \mid t_0)}{p_{\text{fix}}(\bar{s},0 \mid t_0)}\right) \approx \delta s\left(\frac{\tau}{2} - R(t_0)\right), \tag{S98}$$

implying that the time-dependent specialist advantage has the same two-sided exponential form we obtained in the rare specialists regime.

However, calculating the time-averaged specialist advantage

$$\frac{p_{\text{fix}}(\bar{s},\delta s)}{p_{\text{fix}}(\bar{s},0)} = \frac{\int dt_0\; p_{\text{fix}}(\bar{s},\delta s \mid t_0)}{\int dt_0\; p_{\text{fix}}(\bar{s},0 \mid t_0)} \tag{S99}$$

is more difficult, because of the additional time-dependence introduced by the omitted terms in Eq. (S102). These time-dependent terms are not necessarily small and can enter at similar order to the δs term, influencing the value of t_0 that dominates each integral. Nonetheless, we can make an ansatz for the structure of these terms based on the observation that under the optimal mutational path, a successful lineage acquires specialist mutations that arise roughly uniformly over time across each environmental cycle. By enforcing this uniformity as a self-consistent constraint, we can impute the form of the omitted time-dependent terms.

Writing $p_{\text{fix}}(\bar{s},\delta s|t_0) = Q(t_0)\cdot p^0_{\text{fix}}(\bar{s},\delta s|t_0)$, with p^0_{fix} given by Eq. (S102) and Q the unknown correction term, we require that successful mutations arise uniformly across time:

$$\frac{d}{dt_0}\left[\frac{1}{2}p_{\text{fix}}(s,\Delta s|t_0) + \frac{1}{2}p_{\text{fix}}(s,-\Delta s|t_0) + p_{\text{fix}}(s,0|t_0)\right] = 0. \tag{S100}$$

Solving this equation yields

$$\frac{1}{Q(t_0)} \sim 1 + \cosh\left[\Delta s\left(\frac{\tau}{2} - R(t_0)\right)\right] \tag{S101}$$

up to a time-independent constant. The overall time-dependent fixation probability is then

$$p_{\text{fix}}(\bar{s},\delta s \mid t_0) \approx \frac{x_c}{\bar{s}+\zeta(x^*_\perp,t_0)}\sqrt{\frac{2v_\parallel}{\pi}}\left[1+\cosh\left(\Delta s\left(\frac{\tau}{2}-R(t_0)\right)\right)\right]^{-1}\cdot\exp\left[-\frac{(x_c-\bar{s})^2}{2v_\parallel} + \delta s\left(\frac{\tau}{2}-R(t_0)\right) + \frac{x_c\tau\Delta s}{4s} - \frac{v_\perp\tau^2}{24}\right]. \tag{S102}$$

The effect of the new term is to globally reduce the fixation probability for mutations that arise near environmental switches. Intuitively, this is because mutations that arise at these times face heightened competition with specialist mutations on other genetic backgrounds. This adjustment can be observed directly in simulations (most obviously for generalist mutations, where it is the dominant time-dependent effect; Fig. S6).

bioRxiv preprint doi: https://doi.org/10.64898/2026.09.01.748641; this version posted September 4, 2026. The copyright holder for this preprint (which was not certified by peer review) is the author/funder, who has granted bioRxiv a license to display the preprint in perpetuity. It is made available under a CC-BY 4.0 International license.

The overall specialist advantage can be found by averaging Eq. (S102) over time. The time-dependence is sensitive to the relative size of δs and Δs, determining how the tradeoff strength of the focal mutation compares to that of the JDFE. If $|\delta s| = \Delta s$ (a typical mutation), the fixation probability is nearly constant in the first $\sim\tau/2$ generations of its favored environmental state – then rapidly shifts to favor the other type of specialist mutation, with a brief crossover period where generalist mutations are equally likely to fix as specialists. Mutations with weak tradeoffs, $|\delta s| < \Delta s$, will act similarly to generalists with $\delta s = 0$, and are most likely to fix near this crossover region. Conversely, mutations with stronger-than-typical tradeoffs ($|\delta s| > \Delta s$) are most likely to fix near environmental switches, and have an exponential fixation advantage because they systematically outcompete other mutations at those times:

$$\log\left(\frac{p_{\text{fix}}(\bar{s},\delta s)}{p_{\text{fix}}(\bar{s},0)}\right) \approx \begin{cases} 0 & \text{if } |\delta s| \ll \Delta s \\ \log\left(\Delta s \cdot \tau/2\right) & \text{if } |\delta s| \approx \Delta s, \\ (|\delta s| - \Delta s)\cdot \tau/2 & \text{if } |\delta s| \gg \Delta s. \end{cases} \tag{S103}$$

For typical mutations with $\delta s = \Delta s$, the specialist advantage is now *linear* in $\Delta s\tau$, rather than exponential. This is because there are approximately $4/\Delta s$ generations in an environmental cycle where specialist mutations are about as likely to fix as generalists, out of 2τ total. This is in contrast to the rare-specialists regime, where successful specialist mutations mostly occurred at the start of environmental epochs, where their fixation advantage is strongest. Note that this conclusion critically changes in the $\Delta s > s$ regime discussed in SI 3.2.8, where successful mutations are no longer evenly distributed across an environmental cycle.

In addition to the new form of the time dependence, the constant terms in the exponent of Eq. (S102) (coming from the constant term in ζ^*) are also distinct from the rare-specialists regime. The leading-order adjustment to the fixation probability is independent of δs,

$$p_{\text{fix}} \sim \frac{x_c}{\bar{s}}\sqrt{\frac{2v_\parallel}{\pi}}\exp\left[-\frac{1}{2v_\parallel}\left(x_c - \bar{s} - \frac{v_\parallel \Delta s\tau}{4s} + \frac{v_\parallel v_\perp \tau^2}{24x_c}\right)^2\right], \tag{S104}$$

$$\approx \frac{x_c}{\bar{s}}\sqrt{\frac{2v_\parallel}{\pi}}\exp\left[-\frac{1}{2v_\parallel}\left(x_c - \bar{s} - \frac{v_\parallel \Delta s\tau}{6s}\right)^2\right]. \tag{S105}$$

in the asymptotic limit where Eq. (S71) is valid. The new terms in the parentheses will affect the population-wide rate of adaptation $v_\parallel$, discussed in the next section.

3.2.6 Self-Consistent Rate of Adaptation

The presence of frequent specialist mutations will adjust the overall rate of adaptation of the population v, and thereby the large fitness scale x_c, which we have defined in terms of v through Eq. (S60). We can calculate v through the self-consistent relation

$$v_\parallel \approx 2UNs \cdot p_{\text{fix}}, \tag{S106}$$

bioRxiv preprint doi: https://doi.org/10.64898/2026.09.01.748641; this version posted September 4, 2026. The copyright holder for this preprint (which was not certified by peer review) is the author/funder, who has granted bioRxiv a license to display the preprint in perpetuity. It is made available under a CC-BY 4.0 International license.

with p_{fix} defined in Eq. (S105). This equation is solved numerically to obtain the theory curves in Fig. S6, but we can also analytically calculate the leading-order correction to v by balancing the exponential terms in Eq. (S105) with NU_s, yielding:

$$0 \approx \log(NU) - \frac{(x_c - s)^2}{2v_{\|}} + \frac{\Delta s \tau x_c}{6s}, \tag{S107}$$

$$v \approx s^2 \frac{2\log(Ns)}{\log^2(s/U)} + \Delta s \tau s^2 \frac{2\log(Ns)}{3\log^3(s/U)} \tag{S108}$$

after plugging in the definition of x_c from Eq. (S60). We can write the frequent-specialist $v_{\|}$ and x_c in terms of their original values in the rare-specialist regime (denoted as v^0 and x_c^0):

$$v_{\|} \approx v^0 + \frac{\Delta s \tau}{3s} \frac{(v^0)^2}{x_c^0}, \tag{S109}$$

$$x_c \approx x_c^0 + \frac{\Delta s \tau}{3s} v^0. \tag{S110}$$

(Note that the prefactor of this adjustment term may be up to a factor of $3/2$ larger if $v_{\perp}$ is smaller than its asymptotic limit.) Thus, having access to environment-sensitive mutations allows the population to slightly increase its average rate of adaptation. This is consistent with the fact that the benefit of specialist mutations derives from their ability to speed up the rate at which a lineage founds future mutations. If all successful lineages have well-timed specialism, the bulk $v_{\|}$ should be larger as well.

3.2.7 *Asymmetric Fitness Effects and Mutation Rates*

Our analysis of the frequent-specialists regime above assumed that the average fitness effects and mutation rates of specialist and generalist mutations were equal. We will briefly discuss extending our calculation to the more general asymmetric case, focusing on specialist mutations with a lower average fitness than generalists. (Because specialist mutations are already favored over generalist mutations in the symmetric case, an additional benefit to specialist mutations can be treated as simply a higher effective value of s or U.)

Suppose specialist mutations have fitness effects $(s - c, \pm\Delta s)$, with an average fitness cost c relative to generalists. Effectively, each specialist mutation a lineage acquires in lieu of a generalist mutation will impose a rolling fitness cost of c. By accounting for this fitness cost, we can characterize the new population dynamics.

Long-term optimal path. To determine the long-term optimal mutational path, we can compare the time-dependent benefit of specialist mutations to their relative cost of c. We will focus on the crossover regime

$$0 < c < \frac{v \Delta s \tau}{2x_c}, \tag{S111}$$

bioRxiv preprint doi: https://doi.org/10.64898/2026.09.01.748641; this version posted September 4, 2026. The copyright holder for this preprint (which was not certified by peer review) is the author/funder, who has granted bioRxiv a license to display the preprint in perpetuity. It is made available under a CC-BY 4.0 International license.

where the average fitness cost of specialist mutations is competitive with their effective benefit. (For larger c, generalist mutations will tend to outcompete specialist mutations over the long term.) In the crossover regime, generalist mutations will be favored near the middle of each environmental state, such that the optimal mutational path includes each type of mutation:

$$D^*(t) = \begin{cases} 0 & \text{if } |\tau/2 - R(t)| < c \cdot x_c/(v\Delta s), \\ \text{sign}\left[\tau/2 - R(t)\right] & \text{if } |\tau/2 - R(t)| > c \cdot x_c/(v\Delta s). \end{cases} \tag{S112}$$

The fixation probability ratio is given by the fraction of time each mutation is optimal:

$$\frac{p_{\text{fix}}(s-c, \Delta s)}{p_{\text{fix}}(s, 0)} \approx \frac{v\Delta s\tau - 2x_c c}{2x_c c}. \tag{S113}$$

Following a similar procedure as in Eq. (S68), the effective fitness boost from following the optimal mutational path will be reduced,

$$\zeta^*(c) \sim \frac{\Delta s}{4v_\parallel \tau s}\left(v_\parallel \tau - \frac{2x_c c}{\Delta s}\right)^2, \tag{S114}$$

neglecting the $v_\perp$-dependent term (which we expect to adjust numerical factors but not the overall scaling).

Population rate of adaptation. Asymmetric fitness effects have interesting implications for the population-wide rate of adaptation, because they modulate both the relative fixation probability of specialist and generalist mutations as well as their direct fitness effects. The rate of adaptation is defined by the equation

$$v_\parallel = N(s-c)U_s p_{\text{fix}}(s-c, \pm\Delta s) + NsU_g p_{\text{fix}}(s, 0) \tag{S115}$$

$$\approx \left[NU_s(s-c)\frac{v_\parallel \Delta s\tau - 2x_c c}{v_\parallel \Delta s\tau} + NU_g s\frac{2x_c c}{v_\parallel \Delta s\tau}\right] \cdot \exp\left[-\frac{(x_c - s)^2}{2v_\parallel} + \frac{\Delta s x_c}{4s\tau}\left(\tau - \frac{2x_c c}{\Delta s v_\parallel}\right)^2\right] \tag{S116}$$

after plugging in the leading correction to the break-even fitness from Eq. (S114). When U_s and U_g are similar, the solution to this equation can be expressed as

$$v_\parallel \approx v^0 + \frac{\Delta s\tau(v^0)^2}{2sx_c^0}\left(1 - \frac{2x_c^0}{v^0\Delta s\tau}c\right)^2 - c\frac{4v^0 s}{(x_c^0)^2}\left(1 - \frac{2x_c^0}{v^0\Delta s\tau}c\right). \tag{S117}$$

Interestingly, this result is non-monotonic in c. Between $c = 0$ and $c = v^0\Delta s\tau/2x_c - v^0 s^2\Delta s\tau/x_c^3$, the first correction term dominates, and $v_\parallel$ decreases with larger c as the fitness benefit of successful mutations is reduced, eventually dropping below the rare-specialists v^0. But as c increases further toward $v^0\Delta s\tau/2x_c$, costless generalist mutations start to be favored by natural selection, and $v_\parallel$ increases back to v^0. While the non-monotonic range is small in the deterministic regime ($\sigma_\tau = 0$), this basic behavior will also apply in

bioRxiv preprint doi: https://doi.org/10.64898/2026.09.01.748641; this version posted September 4, 2026. The copyright holder for this preprint (which was not certified by peer review) is the author/funder, who has granted bioRxiv a license to display the preprint in perpetuity. It is made available under a CC-BY 4.0 International license.

the stochastic $\sigma_\tau > 0$ case, where the non-monotonicity of $v_\parallel$ can be easily observed in simulations.

Differing mutation rates. We can now easily extend our results to the $U_g \neq U_s$ case by observing that if $U_g > U_s$, a lineage will typically acquire its first generalist mutation before its first specialist mutation. This extra waiting time acts as an effective rolling fitness cost for specialist mutations of

$$c_{\text{eff}} \approx \frac{v_\parallel}{x_c} \log\left(\frac{U_g}{U_s}\right). \tag{S118}$$

Because the effective fitness cost depends only logarithmically on the mutation rate ratio, modest differences in mutation rate (e.g., factors of 2) will have minimal impact on the population dynamics. Generalist mutations will only comprise the majority of the long-term optimal mutational path if

$$U_g \gtrsim U_s \cdot \exp(\Delta s\tau/2). \tag{S119}$$

Note that this exponential factor corresponds to the specialist advantage in the rare-specialist regime. Thus, this transition represents the crossover between the frequent-specialist and the rare-specialist regime: as the specialist mutation rate increases, specialist mutations will begin to become dominant in the population when $U_s p_{\text{fix}}(s, \Delta s) \gtrsim U_g p_{\text{fix}}(s, 0)$.

3.2.8 Stronger Tradeoffs and Specialist Bursting

When calculating the optimal mutational path using the update rules in Eq. (S61a) and Eq. (S61b), we assumed that $s > \Delta s$, so that a mutant strain always grows faster than its parent strain regardless of the environmental state. In this regime, the members of a fitness class will mostly be composed of descendants of the first mutant into that class, so the optimal mutational path always entails acquiring a new mutation as soon as possible.

When $\Delta s > s$, a new type of step in the optimal mutational path becomes relevant: a lineage may acquire *no* mutations for $\sim \tau \gg s/v_\parallel$ generations, but rise to high enough frequencies that it can produce a "burst" of specialist mutations in rapid succession when the environmental state switches. While we will not provide a full summary of this regime in this work, we will derive the new optimal mutational path it implies, and its implications for some of the observables we have discussed in this section.

Rolling fitness of bursting lineages. Consider the process of a lineage acquiring specialist mutations that favor the upcoming environment, which (when $\Delta s > s$) appear deleterious in the current environment state. The key effect is that these fitness classes will no longer be dominated by the *first* mutation into each class – instead, most of their members will be new mutations from the faster-growing parent class that occur right before the environment switches.

To describe this process, we can take advantage of the fact that stochasticity in the time each individual mutation arises becomes largely negligible, because the composition of

bioRxiv preprint doi: https://doi.org/10.64898/2026.09.01.748641; this version posted September 4, 2026. The copyright holder for this preprint (which was not certified by peer review) is the author/funder, who has granted bioRxiv a license to display the preprint in perpetuity. It is made available under a CC-BY 4.0 International license.

each class will be determined by many independent mutational events. Consider a founding lineage at relative fitness $(x_\parallel, x_\perp)$ in a constant environment $I(t) = +1$, with frequency

$$f_0(t) = \frac{1}{2N(x_\parallel + x_\perp)} e^{(x_\parallel + x_\perp)t - (v_\parallel + v_\perp)t^2/2}. \tag{S120}$$

It founds successive classes via mutations that appear deleterious in the current environment, with fitness effect $\alpha \equiv \Delta s - s$. The dynamics of these classes are given by

$$\frac{df_n}{dt} = (x_\parallel + x_\perp - n\alpha - (v_\parallel + v_\perp)t) f_n + U_s f_{n-1}. \tag{S121}$$

We will assume that $x_\parallel + x_\perp - n\alpha - (v_\parallel + v_\perp)t$ remains positive (so new classes can still establish), and that enough time has passed for the focal class to contain several members (so stochasticity in founding mutations can be neglected). The solution to this equation can be written as an integral expression over all the possible times of each mutation,

$$\begin{aligned} f_n(t) &\approx \frac{U^n}{2N(x_\parallel + x_\perp)} \int_{0<t_1<\ldots<t_n}^{t} dt_1 \ldots dt_n \exp\left[(x_\parallel + x_\perp - n\alpha)t - \frac{v_\parallel + v_\perp}{2} t^2 + \alpha \sum_{k=1}^{n} t_k\right] \\ &= \frac{U^n}{2N(x_\parallel + x_\perp)} \frac{1}{n!\alpha^n} e^{(x_\parallel + x_\perp - n\alpha)t - (v_\parallel + v_\perp)t^2/2} \left(e^{\alpha t} - 1\right)^n \qquad \text{(S122)} \\ &\approx \left(\frac{s}{\alpha}\right)^n \frac{1}{2N(x_\parallel + x_\perp)} \frac{1}{n!} \exp\left[(x_\parallel + x_\perp)t - x_c \frac{ns}{v_\parallel} - \frac{v_\parallel + v_\perp}{2} t^2 + \frac{ns^2}{2v_\parallel}\right] \qquad \text{(S123)} \end{aligned}$$

for $t \gg \log(n)/\alpha$. (Loosely, we expect this to be valid when Δs and s are not fine-tuned to be extremely close to each other.) Because the optimal times for each mutation to arise are shortly before the present, the frequency profile of the fitness class with n additional "deleterious" specialist mutations depends only algebraically on α.

Now, suppose the $n+1$st mutation of this type lands in a beneficial environment, meaning that once again only the first mutation founded matters. This mutation will be founded near time T, defined via the integral relation

$$\begin{aligned} 1 &\approx \int_0^T 2NU \left[x_\parallel - (n+1)\alpha + s - (v_\parallel + v_\perp)t\right] f_n(t) dt \qquad \text{(S124)} \\ &\approx \frac{s^{n+1}}{\alpha^n n!} \int_0^T \exp\left[x_\parallel t - \frac{v_\parallel + v_\perp}{2} t^2 - x_c \frac{(n+1)s}{v_\parallel} + \frac{(n+1)s^2}{2v_\parallel}\right] \qquad \text{(S125)} \\ &\approx \frac{s}{x_\parallel} \frac{s^n}{\alpha^n n!} \exp\left[x_\parallel T - \frac{v_\parallel + v_\perp}{2} T^2 - x_c \frac{(n+1)s}{v_\parallel} + \frac{(n+1)s^2}{2v_\parallel}\right] \qquad \text{(S126)} \end{aligned}$$

$$T \approx \frac{(n+1)s}{v_\parallel}\left(1 - \frac{x_\parallel - x_c}{x_c}\right) + \frac{n(n+1)s^2}{2v_\parallel x_c} + \frac{(n+1)^2 s^2 v_\perp}{2v_\parallel^2 x_c} + \mathcal{O}\left(\frac{n^3 s^3 v_\perp^2}{v_\parallel^3 x_c^2}\right). \tag{S127}$$

These dynamics mean that a lineage which acquires a burst of n mutations right when the environment switches will land at rolling fitness

$$x_\parallel(n) - x_\parallel(0) \approx ns \frac{x_\parallel(0) - x_c}{x_c} - \frac{n(n-1)s^2}{2x_c} - \frac{n^2 s^2 v_\perp}{2v_\parallel x_c}, \tag{S128}$$

bioRxiv preprint doi: https://doi.org/10.64898/2026.09.01.748641; this version posted September 4, 2026. The copyright holder for this preprint (which was not certified by peer review) is the author/funder, who has granted bioRxiv a license to display the preprint in perpetuity. It is made available under a CC-BY 4.0 International license.

approximately independent of Δs. Instead, the rolling fitness contains a cost of $\sim n^2 s^2/x_c$, due to the lineage failing to acquire *any* beneficial mutations during the waiting period of $\sim ns/v_\parallel$ generations. However, this cost can be compensated by the benefit of acquiring n perfectly-timed specialist mutations, as we will discuss next.

Optimal mutational path. To derive the optimal mutational path, we will compare the effective fitness benefit of acquiring specialist mutations throughout an environmental cycle to the effective benefit of acquiring a burst of specialist mutations near the boundaries. Earlier, we found that an optimal specialist mutation which arises at time t in a cycle provides the same long-term fitness benefit as a generalist mutation with effect

$$s_{\text{eff}}(t) = s + \frac{v_\parallel \Delta s}{x_c}\left|\frac{\tau}{2} - R(t)\right|. \tag{S129}$$

The *total* effect of acquiring successive specialist mutations between time t_0 and τ (for $0 < t_0 < \tau$) is

$$s_{\text{cycle}}(t_0) = \frac{v_\parallel}{s}\int_{t_0}^{\tau} s_{\text{eff}}(t)dt = v_\parallel(\tau - t_0) + \frac{v_\parallel^2 \Delta s}{s x_c}\left[\frac{\tau^2}{8} + \frac{1}{2}\left(\frac{\tau}{2} - t_0\right)\left|\frac{\tau}{2} - t_0\right|\right]. \tag{S130}$$

We must compare s_{cycle} to the total fitness effect of acquiring a burst of $n = v_\parallel(\tau - t_0)/s$ specialist mutations at time τ, after not acquiring any mutations for $\tau - t_0$ generations. This will include both the direct fitness effects of the mutations, their effective fitness boost from being perfectly-timed specialist mutations, and the cost of waiting to acquire them:

$$s_{\text{burst}}(t_0) \approx ns + \frac{n v_\parallel \Delta s \tau}{2x_c} - \frac{n^2 s^2}{2x_c}, \tag{S131}$$

$$\approx v_\parallel(\tau - t_0) + \frac{v_\parallel^2 \Delta s}{2 s x_c}\left[\tau(\tau - t_0) - \frac{s}{\Delta s}(\tau - t_0)^2\right]. \tag{S132}$$

If $s > \Delta s$, then $s_{\text{cycle}} > s_{\text{burst}}$ for all t_0, consistent with the non-bursting optimal path we analyzed earlier. When $\Delta s > 2s$, then $s_{\text{burst}} > s_{\text{cycle}}$, so the optimal path will *only* involve acquiring bursts of specialist mutations when the environment changes. In the crossover region $2s > \Delta s > s$, s_{burst} becomes larger than s_{cycle} at an intermediate value of t_0 between 0 and $\tau/2$, indicating that the optimal path entails acquiring some number of sequential specialist mutations aligned with the current environment, then waiting to acquire further mutations (of the opposite specialist type) in a burst when the environment switches.

Focusing on the burst-dominated $\Delta s > 2s$ regime, the effective fitness boost caused by following the optimal bursting path is roughly

$$\zeta^*_{\text{burst}}(x_\perp, t_0) \approx \frac{v_\parallel \tau(\Delta s - s)}{2s} + \frac{v_\parallel x_\perp}{x_c}\left(\frac{\tau}{2} - R(t_0)\right) - \frac{v_\parallel v_\perp}{2x_c}\left(\frac{\tau}{2} - R(t_0)\right)^2 - \frac{v_\parallel v_\perp \tau^2}{8x_c}. \tag{S133}$$

When $\Delta s > 2s$, the leading constant term in Eq. (S133) is larger than the corresponding term in the $s > \Delta s$ regime, Eq. (S68), increasing the overall benefit of following the optimal mutational path. However, the overall scaling of this term is similar (changing by at most a

bioRxiv preprint doi: https://doi.org/10.64898/2026.09.01.748641; this version posted September 4, 2026. The copyright holder for this preprint (which was not certified by peer review) is the author/funder, who has granted bioRxiv a license to display the preprint in perpetuity. It is made available under a CC-BY 4.0 International license.

factor of 2), so the correction it induces on the population rate of adaptation v will be qualitatively similar. The $x_\perp$-dependent term is also identical, meaning the time-dependent advantage of specialist mutations remains the same.

Specialist advantage. An important qualitative difference between the $\Delta s > s$ bursting regime and the $s > \Delta s$ non-bursting regime is that when bursts dominate, successful specialist mutations become concentrated near the boundaries of environments, where their fitness advantage over generalists is largest. This means that after averaging over time, the overall specialist advantage $p_{\text{fix}}(\bar{s}, \Delta s)/p_{\text{fix}}(\bar{s}, 0)$ will be exponential in $\Delta s\tau$, as in the rare-specialists regime – but unlike the non-bursting regime in Eq. (S103). While we will not calculate the full time-dependent fixation probability, we roughly expect it to be between the rare-specialists and $s > \Delta s$ predictions. For comparison to simulations, we find that a reasonable approximation is to write a fixation probability identical to the rare-specialists case, with a leading correction coming from the constant shift in ζ^* from Eq. (S105):

$$p_{\text{fix}}(s, \Delta s|t_0) \approx \frac{x_c}{s + \frac{v_\parallel \Delta s\tau}{6s}} \sqrt{\frac{2v_\parallel}{\pi}} \exp\left[-\frac{1}{2v_\parallel}\left(x_c - s - \frac{v_\parallel \Delta s\tau}{6s} - \frac{\Delta s v_\parallel}{x_c}\left(\frac{\tau}{2} - R(t_0)\right)\right)^2\right], \tag{S134a}$$

$$p_{\text{fix}}(s, 0|t_0) \approx \frac{x_c}{s + \frac{v_\parallel \Delta s\tau}{6s}} \sqrt{\frac{2v_\parallel}{\pi}} \exp\left[-\frac{1}{2v_\parallel}\left(x_c - s - \frac{v_\parallel \Delta s\tau}{6s}\right)^2\right]. \tag{S134b}$$

Averaging over time yields

$$\frac{p_{\text{fix}}(s, \Delta s)}{p_{\text{fix}}(s, 0)} \approx \frac{1}{\Delta s\tau} \exp\left[\frac{\Delta s\tau}{2}\left(1 - \frac{v\Delta s\tau}{6sx_c}\right)\right], \tag{S135}$$

which we use to generate our theory curves for simulations where $\Delta s > s$. We emphasize that the time-dependent effective fitness advantage of specialists, Eq. (S129), is the same between the two regimes – so a specialist mutation that arises near the boundaries of an environmental state is exponentially more likely to fix than a generalist, even when $s > \Delta s$. The difference in overall fixation probability stems from the times that successful mutations typically arise: uniformly when $s > \Delta s$, and concentrated near environmental switches when $\Delta s > s$.

3.3 Frequent Specialists with Seasonal Drift

Finally, we will discuss the case of stochastic environmental fluctuations ($\sigma_\tau > 0$) with frequent specialist mutations ($NU_s \gg 1$). While the complexity of accounting for stochastic switching in a fully two-dimensional fitness landscape precludes us from finding a full solution in this work, we can nonetheless roughly estimate the influence of stochasticity on the population dynamics. Following our previous approach, we will track how stochastic environmental switches change the rolling fitness of a lineage, determining the population rate of adaptation and the specialist advantage.

bioRxiv preprint doi: https://doi.org/10.64898/2026.09.01.748641; this version posted September 4, 2026. The copyright holder for this preprint (which was not certified by peer review) is the author/funder, who has granted bioRxiv a license to display the preprint in perpetuity. It is made available under a CC-BY 4.0 International license.

The overall takeaway we will find is that environmental stochasticity reshapes the optimal mutational path to become aligned with upcoming environmental change. This magnifies the advantage of specialist mutations relative to the case without stochasticity. However, the scale of this additional boost is much less dramatic than we observed for rare specialist mutations (SI 2.2.1). This is because when specialist mutations are widely available, the dynamics of all lineages will be subject to random environmental fluctuations, and the relative benefit provided by a single specialist mutation decreases. Nonetheless, environmental stochasticity will still have significant impacts on the fixation probability of mutations and the population rate of adaptation.

We will continue to employ our ansatz in Eq. (S56) expressing the adaptation of the bulk population in terms of $v_{\parallel}$ and $v_{\perp}$. This is a strong approximation, because the bulk rate of specialization $v_{\perp}$ over a given environmental epoch may depend on the realized stochasticity, rather than being described by a single constant (Fig. S10A). Nonetheless, this is an initial linear approximation which reflects the fact that the bulk population will adapt more toward environments that happen to last longer.

3.3.1 Faster Environmental Cycles

In a stochastic environment, it is convenient to focus on the case of faster environmental cycling, $\tau \ll s/v_{\parallel}$, so that many environmental cycles occur in the time it takes for a lineage to found a new mutation. First, we will calculate the impact that environmental stochasticity has over $s/v_{\parallel}$ generations, the time it takes to found a single mutation. We can then extend this result over many foundings to determine how seasonal drift influences the fate of a mutation. (We will ignore the deterministic effects discussed in the previous section, assuming their contribution is small when stochastic effects dominate – this is more reasonable in the smaller-τ regime.)

Consider a lineage at initial frequency f_0, average fitness $x_{\parallel}$, and environmental preference $x_{\perp}$ in an environment aligned with the $+x_{\perp}$ direction. After t_1 generations in a single environment, its frequency will be

$$f(t_1) \approx f_0 \exp\left[(x_{\parallel} + x_{\perp})t_1 - \frac{v_{\parallel} + v_{\perp}}{2} t_1^2\right]. \tag{S136}$$

Then, suppose the environment switches states for t_2 generations. As before, we will neglect stochasticity in the total environmental cycle time and focus on the time difference between environments, assuming that $t_1 + t_2 = 2\tau$ and defining $\delta\tau \equiv t_1 - t_2$. The final frequency of the lineage after a full cycle will be

$$f(2\tau) \approx f_0 \exp\left[2x_{\parallel}\tau - 2v_{\parallel}\tau^2 + x_{\perp}\delta\tau - \frac{v_{\perp}}{2}\delta\tau^2\right]. \tag{S137}$$

Each environmental cycle will have a new stochastic value of $\delta\tau$, perturbing the frequency

bioRxiv preprint doi: https://doi.org/10.64898/2026.09.01.748641; this version posted September 4, 2026. The copyright holder for this preprint (which was not certified by peer review) is the author/funder, who has granted bioRxiv a license to display the preprint in perpetuity. It is made available under a CC-BY 4.0 International license.

of the lineage. After n such cycles, the frequency of the lineage will be

$$f(2n\tau) \approx f_0 \exp\left[2nx_{\parallel}\tau - 2v_{\parallel}(n\tau)^2 + x_{\perp}\sum_{i=1}^{n}\delta\tau_i - \frac{v_{\perp}}{2}\sum_{i=1}^{n}\delta\tau_i^2 - v_{\perp}\sum_{j=2}^{n}\sum_{i=1}^{j-1}\delta\tau_j\delta\tau_i\right]. \tag{S138}$$

A newly established lineage must undergo $n \approx s/2v_{\parallel}\tau$ such cycles in order to found a new beneficial mutation. The net effect of the stochasticity over this time will determine the lineage's rolling fitness after the n cycles are complete. We can use this to define new recursive relations where each step represents the founding of a new mutation, in terms of $\delta x \equiv x_{\parallel} - x_c$:

$$\delta x_{J+1} \approx \delta x_J\left(1 + \frac{s}{x_c}\right) + \frac{v_{\parallel}}{x_c}\left(x_{\perp}^J\Delta T_J - \frac{v_{\perp}}{2}\Delta T_J^2\right), \tag{S139}$$

$$x_{\perp}^{J+1} \approx x_{\perp}^J + \Delta s \cdot D_J - v_{\perp}\Delta T_J, \tag{S140}$$

where D_J is the environmental preference of the Jth mutation, and

$$\Delta T_J \equiv \sum_{i=1+J(s/2v_{\parallel}\tau)}^{(J+1)(s/2v_{\parallel}\tau)} \delta\tau_i \tag{S141}$$

is the net environmental stochasticity over the time period during which the focal lineage founds its $J+1$st mutation. The solution to these recursion relations is

$$\delta x_K e^{-sK/x_c} \approx \delta x_0 + \xi(\boldsymbol{D}, \boldsymbol{\delta\tau}) \tag{S142}$$

with

$$\xi(\boldsymbol{D}, \boldsymbol{\delta\tau}) \equiv \frac{v_{\parallel}}{x_c}\left[x_{\perp}^0 A_0 + \sum_{J=1}^{K-1} e^{-sJ/x_c} A_J\left(\Delta s D_{J-1} - v_{\perp}\Delta T_{J-1}\right) - \frac{v_{\perp}}{2}\sum_{J=1}^{K} e^{-sJ/x_c}\Delta T_{J-1}^2\right] \tag{S143}$$

an effective shift in the starting fitness, and

$$A_J \equiv \sum_{i=1}^{K-J} e^{-si/x_c}\Delta T_{J+i-1} \tag{S144}$$

a weighted sum of the environmental stochasticity after the time indexed by J. We observe that the optimal mutational path is

$$D_J^*(\boldsymbol{\delta\tau}) = \mathrm{sign}(A_{J+1}), \tag{S145}$$

such that optimally specializing lineages should "anticipate" the upcoming environmental bias. The survival of the focal lineage depends on whether

$$\xi^*(\boldsymbol{\delta\tau}) \equiv \lim_{K\to\infty} \xi(\boldsymbol{D}^*, \boldsymbol{\delta\tau}) \tag{S146}$$

bioRxiv preprint doi: https://doi.org/10.64898/2026.09.01.748641; this version posted September 4, 2026. The copyright holder for this preprint (which was not certified by peer review) is the author/funder, who has granted bioRxiv a license to display the preprint in perpetuity. It is made available under a CC-BY 4.0 International license.

is greater than $-\delta x_0$, meaning that the probability of survival will strongly depend on the distribution of ξ^*, and in particular its positive tail under the optimal mutational path.

Characterizing the stochasticity. The distribution of ξ^* is complicated due to the strong correlations between the A_J, so we will generally characterize it through semi-analytic approximations based on fitting to empirical samples. Note that ξ^* only depends on environmental stochasticity and not the dynamics of mutations, so its distribution can be sampled straightforwardly by drawing a set of epoch times.

To leading order in s/x_c, the mean and variance of ξ^* are

$$\langle \xi^* \rangle \approx -\frac{v_\perp \sigma_\tau^2}{2\tau} + \frac{\Delta s \sigma_\tau}{\sqrt{\pi}} \sqrt{\frac{v}{x_c \tau}} \frac{x_c}{s}, \tag{S147}$$

$$\mathrm{Var}\left(\xi^*\right) \approx \frac{\sigma_\tau^2}{x_c} \frac{v}{\tau} \left[\frac{(x_\perp^0)^2}{2} + \left(\frac{3}{8} - \frac{1}{\pi}\right) \frac{x_c^2 \Delta s^2}{s^2} - \frac{\Delta s \sigma_\tau v_\perp}{3\sqrt{\pi}} \frac{x_c}{s} \sqrt{\frac{x_c}{v\tau}} + \frac{v_\perp^2 \sigma_\tau^2 x_c}{4 v \tau} \right]. \tag{S148}$$

However, the most relevant quantity for calculating the influence of ξ^* on fixation is its survival function $S(z)$ in the positive tail, indicating the effective fitness benefit it provides given lucky environmental stochasticity. We find that a reasonable phenomenological fit to this survival function over a range of parameters is a Gaussian which approaches a cutoff at some scale $\xi_{\max}$ (Fig. S8A):

$$S(z) \equiv \mathbb{P}\left[\xi^* > z\right] \approx \frac{1}{2} \mathrm{erfc}\left(\frac{z - \mu_{\text{eff}}}{\sqrt{2} \cdot \sigma_{\text{eff}}}\right) \cdot \exp\left[-\log^2\left(1 - \frac{\max(z, 0)}{\xi_{\max}}\right)\right]. \tag{S149}$$

To reduce the number of free fitting parameters, we approximate $\mu_{\text{eff}} = \langle \xi^* \rangle$ from the analytic expression in Eq. (S147). Furthermore, Eq. (S143) shows that ξ^* is a quadratic form in the ΔT, suggesting that the cutoff can be expressed as the maximum over the ΔT:

$$\xi_{\max} \approx \frac{v_\parallel}{2 v_\perp x_c} \left(\frac{\Delta s^2}{(e^{s/x_c} - 1)^2} + e^{-s/x_c} (x_\perp^0)^2 \right) \tag{S150}$$

$$\approx \frac{v_\parallel}{2 v_\perp x_c} \left(\frac{x_c^2}{s^2} \Delta s^2 + (x_\perp^0)^2 \right) \tag{S151}$$

This leaves the Gaussian spread σ_{eff} as the only free fitting parameter for the survival function at a particular combination of variables. The analytic dependence of σ_{eff} on the evolutionary parameters and initial specialism $x_\perp^0$ does not admit an obvious form, because σ_{eff} represents not the total variance in ξ^*, but the variance in the positive tail (before the cutoff becomes relevant). Thus, we often use the fit values of σ_{eff} directly. Nonetheless, when a semianalytic form os σ_{eff} is necessary, we find that for relatively small $x_\perp^0$ it can roughly be described by a quadratic ansatz (Fig. S8B):

$$\sigma_{\text{eff}}(x_\perp^0) \approx \sigma_{\text{eff}}^0 + \frac{(x_\perp^0)^2}{\alpha}. \tag{S152}$$

The intercept σ_{eff}^0 appears approximately linear in Δs, suggesting it is dominated by the second term in the brackets in Eq. (S148):

$$\sigma_{\text{eff}}^0 \approx C_0 \cdot \sqrt{\frac{v x_c}{\tau}} \frac{\sigma_\tau \Delta s}{s}, \tag{S153}$$

bioRxiv preprint doi: https://doi.org/10.64898/2026.09.01.748641; this version posted September 4, 2026. The copyright holder for this preprint (which was not certified by peer review) is the author/funder, who has granted bioRxiv a license to display the preprint in perpetuity. It is made available under a CC-BY 4.0 International license.

with C_0 a numerical fitting constant accounting for the effective scaling of the variance in the positive tail (Fig. S8C). A quadratic dependence on $x_\perp^0$ is consistent with a Taylor expansion of $\mathrm{Std}(\xi^*)$, suggesting a scaling of

$$\alpha \approx C_\alpha \cdot \sqrt{\frac{x_c^3 \tau}{v}} \frac{\Delta s}{s\sigma_\tau}. \tag{S154}$$

We emphasize that these are rough phenomenological fits for the parameter range used in simulations, meant to broadly characterize the distribution of ξ^*. However, they are sufficient to illustrate the key qualitative upshot, which is how the tail of ξ^* depends on the initial specialism $x_\perp^0$ through σ_{eff} and $\xi_{\max}$.

Fixation probabilities. Following our previous approach in Eq. (S46), we approximate the fixation probability as a double integral over the background mean fitness $x_\parallel$ and specialism $x_\perp$:

$$\begin{aligned} p_{\text{fix}}(\bar{s}, \delta s) &\approx 2x_c \int_{-x_c+\bar{s}}^{x_c+\bar{s}} dx_\parallel \int_{-mx_c}^{mx_c} dx_\perp \frac{e^{-(x_\parallel-\bar{s})^2/2v-(x_\perp-\delta s)^2/2v_\perp}}{2\pi\sqrt{vv_\perp}} \\ &\quad \cdot \int_{-x}^{\infty} d\xi \; \rho_{x_\perp}(\xi) \cdot \min\left[e^{\left[(x_\parallel+\xi)^2-x_c^2\right]/2v},\ 1\right] \end{aligned} \tag{S155}$$

$$\begin{aligned} &= 2x_c \int_{-x_c+\bar{s}}^{x_c+\bar{s}} dx_\parallel \int_{-mx_c}^{mx_c} dx_\perp \frac{e^{-(x_\parallel-\bar{s})^2/2v-(x_\perp-\delta s)^2/2v_\perp}}{2\pi\sqrt{vv_\perp}} \\ &\quad \cdot \left[S_{x_\perp}(-x_\parallel)e^{-x_c^2/2v} + \frac{1}{v}\int_{-x_\parallel}^{x_c-x_\parallel} d\xi \; S_{x_\perp}(\xi)\;(x_\parallel+\xi)e^{\left[(x_\parallel+\xi)^2-x_c^2\right]/2v}\right] \end{aligned} \tag{S156}$$

with $S_{x_\perp}$ the survival function of ξ^* at a given $x_\perp$. Here, m is a constant setting the integration bounds of $x_\perp$. Our theory assumes that $|x_\perp| < x_c$ so that a new mutation can escape genetic drift regardless of the environmental state, but allowing the integral to extend past this bound by setting $m = 3$ allows for better agreement with simulations.

Our approximations are not sufficient to recover the precise form of p_{fix}, but they do accurately predict the specialist advantage $p_{\text{fix}}(s, \Delta s)/p_{\text{fix}}(s, 0)$ at small stochasticity (Fig. S7B). This advantage comes from the fact that if the focal mutation has a tradeoff $\Delta s > 0$, the initial lineage can attain a slightly higher value of specialism $|x_\perp|$, which widens the survival function of ξ (Fig. S8A). However, this shift in the survival function is relatively modest compared to the rare-specialists case, where the focal specialist was the *only* mutation making a lineage sensitive to environmental change.

To produce the theory curves in Fig. S7B, we numerically integrate Eq. (S156) using values of $\sigma_{\text{eff}}(x_\perp)$ inferred from the survival function of ξ^*. This approximation fails at the larger values of Δs we study in Fig. 4C, likely due to to $|x_\perp|$ becoming large enough relative to x_c. To get more intuition for the scaling at small Δs, we can assume cutoff effects are negligible. In this case, the calculation proceeds identically to the rare-specialists case until

bioRxiv preprint doi: https://doi.org/10.64898/2026.09.01.748641; this version posted September 4, 2026. The copyright holder for this preprint (which was not certified by peer review) is the author/funder, who has granted bioRxiv a license to display the preprint in perpetuity. It is made available under a CC-BY 4.0 International license.

Eq. (S49), except σ_ξ is now replaced by $\sigma_{\text{eff}}(x_\perp)$:

$$p_{\text{fix}}(\bar{s}, \delta s) \sim \int_{-mx_c}^{mx_c} dx_\perp \exp\left[-\frac{(x_\perp - \delta s)^2}{2v_\perp} - \frac{Z^2}{2v} + \frac{Z^2}{2v^2}\sigma_{\text{eff}}^2(x_\perp)\right] \tag{S157}$$

$$\sim \int_{-mx_c}^{mx_c} dx_\perp \exp\left[-\frac{(x_\perp - \delta s)^2}{2v_\perp} - \frac{Z^2}{2v} + \frac{Z^2}{2v^2}\left((\sigma_{\text{eff}}^0)^2 + \frac{2\sigma_{\text{eff}}^0 x_\perp^2}{\alpha}\right)\right] \tag{S158}$$

$$\sim \exp\left[-\frac{Z^2}{2v} + \frac{Z^2\sigma_{\text{eff}}^0\left(\sigma_{\text{eff}}^0 + 2\delta s^2/\alpha\right)}{2v^2}\right] \tag{S159}$$

$$\frac{p_{\text{fix}}(\bar{s}, \Delta s)}{p_{\text{fix}}(\bar{s}, 0)} \sim \exp\left[\frac{\Delta s^2(x_c - \bar{s} - \mu_{\text{eff}})^2\sigma_{\text{eff}}^0}{\alpha v^2}\right] \tag{S160}$$

with $Z \equiv x_c - \bar{s} - \mu_{\text{eff}}$. Plugging in our rough scaling forms for σ_{eff}^0 and α yields

$$\frac{p_{\text{fix}}(\bar{s}, \Delta s)}{p_{\text{fix}}(\bar{s}, 0)} \sim \exp\left[\frac{C_0}{C_\alpha}\frac{\sigma_\tau^2\Delta s^2(x_c - \bar{s} - \mu_{\text{eff}})^2}{v x_c \tau}\right]. \tag{S161}$$

This scaling is similar to the rare-stochasticity scaling in Eq. (S49), up to an exponential scaling prefactor of $C_0/C_\alpha \approx 0.1$ and the μ_{eff} shift in the exponent. However, these approximations assume the cutoff is negligible and that stochasticity is weak enough to be treated as a small perturbation, $v_\parallel^2 \gg v_\perp \sigma_{\text{eff}}^0 x_c^2/\alpha$. These assumptions fail above $\Delta s \gtrsim 0.015$ for our parameters, suppressing the specialist advantage (Fig. S7B).

To estimate the specialist advantage over a wider range of Δs and produce the theory curves in Fig. 4C, we use a simple ansatz for the fixation probability representing how typical values of environmental stochasticity ξ^* produce an effective fitness shift. To approximate this shift, we assume the term in $\text{Var}(\xi^*)$ corresponding to Eq. (S153) dominates the variance:

$$\xi_{\text{typ}}^* \approx \sqrt{\frac{2}{\pi}} \cdot \sqrt{\frac{3}{8} - \frac{1}{\pi}} \cdot \sqrt{\frac{v_\parallel x_c}{\tau}}\frac{\sigma_\tau \Delta s}{s}. \tag{S162}$$

This shift comes from each of the specialist mutations a lineage acquires on its way to fixation. A single specialist mutation contributes a fraction $\sim s/x_c$ of this shift. So, the fixation probability of each type of mutation will be roughly

$$p_{\text{fix}}(\bar{s}, \Delta s) \approx \frac{x_c}{\bar{s}}\sqrt{\frac{2v_\parallel}{\pi}} \exp\left[-\frac{1}{2v_\parallel}\left(x_c - \bar{s} - \xi_{\text{typ}}^* - \frac{s}{2x_c}\xi_{\text{typ}}^*\right)^2\right], \tag{S163}$$

$$p_{\text{fix}}(\bar{s}, 0) \approx \frac{x_c}{\bar{s}}\sqrt{\frac{2v_\parallel}{\pi}} \exp\left[-\frac{1}{2v_\parallel}\left(x_c - \bar{s} - \xi_{\text{typ}}^* + \frac{s}{2x_c}\xi_{\text{typ}}^*\right)^2\right], \tag{S164}$$

$$\log\left(\frac{p_{\text{fix}}(\bar{s}, \Delta s)}{p_{\text{fix}}(\bar{s}, 0)}\right) \approx \frac{s}{v_\parallel}\xi_{\text{typ}}^*. \tag{S165}$$

This result broadly agrees with the low-Δs results for our parameter values (Fig. S7B) and has better agreement with simulations for larger Δs, so we use it as a rough comparison to simulations.

bioRxiv preprint doi: https://doi.org/10.64898/2026.09.01.748641; this version posted September 4, 2026. The copyright holder for this preprint (which was not certified by peer review) is the author/funder, who has granted bioRxiv a license to display the preprint in perpetuity. It is made available under a CC-BY 4.0 International license.

Rate of adaptation. To estimate the influence of stochasticity on the rate of adaptation $v_\parallel$, we employ the simple ansatz above and solve for $v_\parallel$ self-consistently:

$$p_{\text{fix}}(\bar{s}) \approx \frac{x_c}{s}\sqrt{\frac{2v_\parallel}{\pi}} \exp\left[-\frac{1}{2v_\parallel}\left(x_c - \bar{s} - \xi^*_{\text{typ}}\right)^2\right], \tag{S166}$$

$$v_\parallel \approx N(U_g + U_s)s \cdot p_{\text{fix}}(s). \tag{S167}$$

Numerically solving Eq. (S167) yields a reasonably accurate solution for $v_\parallel$ over the entire range of Δs we study. To leading order, the correction is

$$v_\parallel \approx v^0 + \frac{2v^0}{x_c}\xi^*_{\text{typ}}, \tag{S168}$$

$$\approx v^0\left(1 + 0.38 \cdot \frac{\sigma_\tau}{\tau}\frac{\Delta s}{s}\sqrt{\frac{v^0\tau}{x^0_c}}\right) \tag{S169}$$

While this adjustment is nominally small, it can be large enough to increase v^0 by about a factor of two for the values of Δs and σ_τ we use in simulations.

3.3.2 *Slower Environmental Cycles*

When $\tau \gg s/v_\parallel$, we can take essentially the same approximate approach to characterize the population dynamics as in the faster-cycling $\tau \ll s/v_\parallel$ regime, but with rescaled effective parameters. In the slower-cycling regime, a lineage can acquire many specialist mutations in a single environmental cycle. However, we expect the optimal mutational path to include long sequences of the same type of specialist mutation, since when σ_τ is reasonably large, the optimal mutations are mostly set by future stochasticity. Under this approximation, we assume that our focal lineage acquires only one type of mutation during each environmental cycle, for a total of $2v\tau/s$ specialist mutations.

The rolling fitness update rule corresponding to Eq. (S139) is then

$$\delta x_{J+1} \approx \delta x_J\left(1 + \frac{2v_\parallel\tau}{x_c}\right) + \frac{v_\parallel}{x_c}\left(x^J_\perp\delta\tau_J - \frac{v_\perp}{2}\delta\tau_J^2\right), \tag{S170}$$

$$x^{J+1}_\perp \approx x^J_\perp + \frac{2v_\parallel\tau}{s}\Delta s D_J - v_\perp\delta\tau_J, \tag{S171}$$

where J indices represent complete environmental cycles. These update rules can be roughly mapped to Eq. (S139), but with s replaced by $2v_\parallel\tau$, and Δs rescaled by an additional factor of $2v_\parallel\tau/s$. This transformation preserves the ratio $\Delta s/s$, which allows us to apply the results from the faster-cycling regime directly.

Our results are somewhat less accurate than in the smaller-τ regime (Fig. 4C, top). One possible reason for this discrepancy is that when multiple new fitness classes arise each environmental cycle, the optimal mutational path will be a mix of the periodic-environment

bioRxiv preprint doi: https://doi.org/10.64898/2026.09.01.748641; this version posted September 4, 2026. The copyright holder for this preprint (which was not certified by peer review) is the author/funder, who has granted bioRxiv a license to display the preprint in perpetuity. It is made available under a CC-BY 4.0 International license.

and stochastic-environment contributions. Here, we have neglected these details and focused on the stochastic contribution only.

Regardless of the precise specialist advantage, the key takeaway from this section is that all the specialist mutations a lineage acquires on its way to fixation or extinction make its trajectory highly sensitive to environmental stochasticity. Successful lineages will acquire mutations which benefit from environmental stochasticity, acting like a deterministic fitness benefit that speeds up the overall rate of adaptation of the population. However, because all lineages can acquire specialist mutations, the advantage of a single specialist mutation is attenuated relative to the rare-specialists regime, where only one lineage experiences environmental stochasticity. A more precise analysis of this stochastic regime is a compelling avenue for future work.

3.3.3 *Rate of Specialization*

The last unknown parameter we have not calculated is the rate of specialization $v_\perp$, which enters in the specialist fixation probability. We can estimate this quantity using a procedure similar to the deterministic case in SI 3.2.3, by tracking how the net specialism a lineage accrues depends on environmental stochasticity (Fig. S10A).

Once again, we define $D_{\text{tot}}(n)$ as the sum of the mutations an optimally-specializing lineage has accrued just before founding its kth mutation. These mutations will depend on the stochasticity according to Eq. (S145):

$$D_{\text{tot}}(n) \equiv \sum_{i=0}^{n-2} D_i^*(n), \tag{S172}$$

$$= \sum_{i=0}^{n-2} \text{sign}\left[\sum_{j=1}^{n-i-1} e^{-sj/x_c} \Delta T_{i+j}\right]. \tag{S173}$$

The rate of specialization $v_\perp$ encodes how sensitive $D_{\text{tot}}(n)$ is to the environmental bias just before observation, ΔT_{n-1}. This environmental bias may have an influence on the optimal D_i^* up to a coalescence time ago, $n \approx x_c/s$. Under our approximation of a constant $v_\perp$, we therefore expect $D_{\text{tot}}(n)$ to be an approximately linear function of ΔT_{n-1} for typical values of ΔT. We will find this linear slope by first writing out the dependence explicitly:

$$D_{\text{tot}}(n) = \sum_{i=0}^{n-2} \text{sign}\left[e^{-s(n-i-1)/x_c} \Delta T_{n-1} + \sum_{j=1}^{n-i-2} e^{-sj/x_c} \Delta T_{i+j}\right]. \tag{S174}$$

We will treat ΔT_{n-1} as a fixed bias parameter, while marginalizing over the distributions of all the other ΔT_i. Approximating them as i.i.d. Gaussians with width $\sigma_\tau\sqrt{s/v_\parallel \tau}$, we

bioRxiv preprint doi: https://doi.org/10.64898/2026.09.01.748641; this version posted September 4, 2026. The copyright holder for this preprint (which was not certified by peer review) is the author/funder, who has granted bioRxiv a license to display the preprint in perpetuity. It is made available under a CC-BY 4.0 International license.

can write the expectation of D_{tot} in terms of the error function:

$$\langle D_{\text{tot}} | \Delta T_{n-1} \rangle \approx \text{sign}\left(\Delta T_{n-1}\right) + \sum_{i=1}^{n-2} \text{erf}\left[\frac{\Delta T_{n-1}}{\sigma_\tau}\sqrt{\frac{v_\| \tau}{2s}} \cdot \sqrt{\frac{e^{-2is/x_c}\left(1 - e^{-2s/x_c}\right)}{1 - e^{-2is/x_c}}}\right], \quad \text{(S175)}$$

re-indexing i so smaller i are closer to the present at k. Smaller i closer to the present are more likely to be influenced by the direction of ΔT_{k-1}, while ΔT_{k-1} has a relatively small influence on terms with large i further in the past.

To obtain the linearized behavior for small ΔT_{n-1} and $i \lesssim x_c/s$, we can expand the exponents for $s/x_c \ll 1$ and the error function for small arguments. Dropping the trivial sign term, we find

$$\langle D_{\text{tot}} | \Delta T_{n-1} \rangle \approx \sqrt{\frac{2v_\| \tau}{\pi s}} \frac{\Delta T_{n-1}}{\sigma_\tau} \sum_{i=1}^{x_c/s} \left(\frac{1}{\sqrt{i}} - \frac{s}{2x_c}\sqrt{i}\right) \quad \text{(S176)}$$

$$\approx \sqrt{\frac{2v_\| \tau}{\pi s}} \frac{\Delta T_{n-1}}{\sigma_\tau} \left(\frac{5}{3}\sqrt{\frac{x_c}{s}} + 1.46\right). \quad \text{(S177)}$$

The rate of specialization is simply the linear coefficient of ΔT_{n-1} times Δs:

$$v_\perp \approx \frac{\Delta s \sqrt{2v_\| \tau x_c/\pi}}{s\sigma_\tau} \left(\frac{5}{3} - 1.46\sqrt{\frac{s}{x_c}}\right) \quad \text{(S178)}$$

Notably, the rate of specialization *declines* with increasing stochasticity, and is markedly less than the periodic case (Fig. S10B). Intuitively, this is because more periodic environments allow for more standing diversity in specialization levels, while stochastic environments will lose some diversity when the environment becomes biased to one state for a period of time.

The same argument made in SI 3.3.2 holds when calculating $v_\perp$ in the slower-cycling $\tau \gg s/v_\|$ regime. Replacing s with $2v_\| \tau$ and Δs with $\Delta s \cdot 2v_\| \tau/s$ leaves Eq. (S178) unchanged to leading order, but adjusts the correction term:

$$v_\perp^{\text{slow cycling}} \approx \frac{\Delta s \sqrt{2v_\| \tau x_c/\pi}}{s\sigma_\tau} \left(\frac{5}{3} - 1.46\sqrt{\frac{2v\tau}{x_c}}\right). \quad \text{(S179)}$$

As in the periodic-cycling regime, these expressions for $v_\perp$ are not fully accurate in the regime studied in simulations, but are largely correct for small Δs and predict the correct qualitative dependence (Fig. S10B).

4 Simulated Population Dynamics

4.1 Overview

Throughout this work, we compare our theoretical predictions to results inferred from simulated Wright-Fisher population dynamics. It is convenient to represent the population

bioRxiv preprint doi: https://doi.org/10.64898/2026.09.01.748641; this version posted September 4, 2026. The copyright holder for this preprint (which was not certified by peer review) is the author/funder, who has granted bioRxiv a license to display the preprint in perpetuity. It is made available under a CC-BY 4.0 International license.

as a three-dimensional matrix (one dimension for each type of mutation), where each entry represents the number of individuals with that specific combination of mutations. The basic procedure is as follows:

1. Initialize a monoclonal population of size N with no mutations in an arbitrary environmental state. Draw the time before the next environmental switch from the distribution $\mathrm{Gamma}(\tau^2/2\Delta\tau^2, \Delta\tau^2/\tau)$.

2. Calculate the absolute fitness λ_i of each mutation class, based on the current environmental state.

3. Perform a selection step by setting $N_i = N \cdot \mathrm{Poisson}\left(N_i e^{\lambda_i} / \sum_j N_j e^{\lambda_j}\right)$. This roughly corresponds to a generation of time.

4. Sample the number of mutations of each type within each mutation class as $\mathrm{Binomial}(N_i, U)$ with $U = U_g$ (for generalists) or $U_s/2$ (for each type of generalist). Update N_i accordingly, extending the population matrix to include a new mutation class if necessary.

5. Increment the time by 1 and check if the new time is after the next environmental switching time. If it is, switch the environmental state and sample the next waiting time as in Step 1.

6. Record the simulation state and remove empty slices of the population matrix. Then, return to Step 2 and repeat until the total simulation time is complete.

Because we often simulate large population sizes of $\mathcal{O}(10^{20})$, the size of sufficiently large mutation classes are stored as floating-point numbers rather than integers. To ensure numerical stability, we use approximations to the above probability distributions when their arguments are sufficiently large. In particular, we approximate the binomial distribution as Poisson when $N_i > 10^5$ and $N_i U(1-U) < 10$, and as a normal distribution when $N_i > 10^5$ and $N_i U(1-U) > 10$. We approximate the Poisson distribution as a normal distribution when its mean is greater than 10^5.

Fixation probabilities can be inferred from this distribution by tracking the average number of each mutation type in the population over time and performing a linear fit. The total number of mutations in the population scales with $t \cdot NUp_{\mathrm{fix}}$ for long times, so dividing the slope of the fit by N and the mutation rate yields the fixation probability.

4.2 Test Lineages

While the above procedure accurately simulates the model of interest, keeping track of mutation classes rather than individual lineages obscures how fixation probabilities depend on other stochastic factors, such as the time a mutation establishes or the background it appears on in the population. Furthermore, when we consider rare specialist mutations, we would like to consider situations when only one specialist mutation is present in the

bioRxiv preprint doi: https://doi.org/10.64898/2026.09.01.748641; this version posted September 4, 2026. The copyright holder for this preprint (which was not certified by peer review) is the author/funder, who has granted bioRxiv a license to display the preprint in perpetuity. It is made available under a CC-BY 4.0 International license.

popoulation at a time. We can remedy both of these issues by introducing "test lineages" in the simulation.

Test lineages are parallel population matrices descended from the main population, which interact with each other and the main population just like any other individual, but are kept track of separately in numerics. If a test lineage dies or reaches fixation, a new test lineage is introduced, so that there is at most one test lineage in the population at any given time (in the case that the previous test lineage fixed, it becomes the new background population). When specialist mutations are rare, we can set $U_s = 0$ for the above procedure and treat the test lineages as their own "specialist" class, with the introduction of a test lineage effectively being a rare specialist mutation event. When specialist mutations are common, both the background population and the test lineage are full three-dimensional matrices free to explore mutational space.

In order to accurately predict fixation probabilities, test lineages should be introduced into the population in a manner which is statistically indistinguishable from how specialist mutations already arise. Specifically, they should start as single individuals, with a mutational background selected proportionally to its frequency in the population. However, most random mutations in the population will not reach fixation, meaning that almost all test lineages introduced this way will soon die. In order to more efficiently estimate fixation probabilities from test lineages, we implement a method of importance sampling which makes test lineages more likely to arise on rare backgrounds, and less likely to die due to genetic drift immediately after being introduced. We can then correct for this importance sampling when estimating the overall fixation probability.

The procedure for introducing test lineages is summarized as follows, which occurs directly after the mutation step above (Step 4):

1. If there are no test lineages in the population, set a time for the next one to appear.
   (a) If $\Delta\tau = 0$, choose this time uniformly over the next complete environmental cycle (starting from the next environmental switch time).
   (b) If $\Delta\tau > 0$, first calculate the probability the test lineage arises during the next cycle as $\mathcal{P}\left[\text{Expon}(\tau) < T_{\text{next}}\right]$, where T_{next} is the duration of the next environmental cycle. If it is slated to arise in the next cycle, sample the time uniformly as in (a). (This decreases the probability the test lineage arises during abnormally short cycles.)
2. If it is time for a test lineage to arise, initialize it.
   (a) Choose the mutational background it arises on *uniformly* from all possible backgrounds (i.e. nonzero entries of the population matrix). This makes all backgrounds equally likely, regardless of their frequencies in the population. Save $w \equiv n_{\text{bg}} f_i$, where n_{bg} is the number of possible backgrounds and f_i is the frequency of the chosen background in the population. This is an importance sampling reweighting factor which we will need later.

bioRxiv preprint doi: https://doi.org/10.64898/2026.09.01.748641; this version posted September 4, 2026. The copyright holder for this preprint (which was not certified by peer review) is the author/funder, who has granted bioRxiv a license to display the preprint in perpetuity. It is made available under a CC-BY 4.0 International license.

(b) The test lineage will have mutations corresponding to its chosen background, plus an additional (random) specialist mutation. Set its initial number as $N_{\text{start}} \equiv \max\left[1, (2(s+\Delta s)n_{\text{muts}})^{-1}\right]$, where n_{muts} is the total number of mutations the new test lineage has (across all types). This allows the test lineage to start at a frequency above $1/N$, but still in the regime dominated by genetic drift.

(c) Set $w = w/N_{\text{start}}$ to account for the probability a newly introduced lineage will drift to N_{start} rather than going extinct.

(d) Record the time the lineage was initialized, its background, its reweighting factor w, and any other desired information.

3. If a test lineage is present, it follows the same selection and mutation steps as any other individual. Descendants of the test lineage remain in the test lineage, so the frequency of the test lineage represents the frequency of the new specialist mutant it introduced in the population.

4. If the test lineage reaches fixation or dies, record its fate. If it reached fixation, the background population will be at zero frequency, so set the test lineage as the new background population and start from Step 1.

The true fixation probability can be estimated from a collection of M test lineages:

$$p_{\text{fix}} \approx \frac{1}{M}\sum_{j=1}^{M} w_j \mathbb{I}_j, \tag{S180}$$

where $\mathbb{I}_j = 1$ if the jth test lineage reached fixation, and 0 if it went extinct. The uncertainty of this estimate is roughly the standard error of the mean among the $w_j\mathbb{I}_j$. In practice, this estimate of p_{fix} is noisier than a linear fit across frequent mutations, but allows for reasonably accurate estimations of extremely low values of $p_{\text{fix}} \sim 10^{-15}$ from only $\sim 10^5$ test lineages.

4.3 Fixation Probability Isoclines

In Fig. 6, we plot fixation probability isoclines, curves of constant fixation probability in two-dimensional fitness effect space. To obtain the simulation points corresponding to these curves, we empirically measured the fixation probability of "test charge" mutations with fitness effects $(s-c, \Delta s)$ for a range of values of c and Δs. We then compared this probability to the fixation probability of generalist mutations with fitness effect $(s, 0)$, fitting a line to their log ratio to estimate the critical value c^* at which the fixation probabilities are equal. Repeating this procedure over a range of Δs produced the empirical fixation probability isoclines.

The theoretical predictions were derived from our previous results for $p_{\text{fix}}(s, \delta s)$, with minor modifications. For rare specialists in a stochastic environment, cutting off the stochasticity at 3 standard deviations as in Fig. S1 produced the most accurate results, and the isoclines were extended linearly past the region where our theoretical prediction for p_{fix}

bioRxiv preprint doi: https://doi.org/10.64898/2026.09.01.748641; this version posted September 4, 2026. The copyright holder for this preprint (which was not certified by peer review) is the author/funder, who has granted bioRxiv a license to display the preprint in perpetuity. It is made available under a CC-BY 4.0 International license.

broke down. For frequent specialists in a stochastic environment, the deterministic contribution to the effective fitness shift was comparable to the stochastic contribution, so we added them together to produce the stochastic-environment isoclines.
